# The evolutionary origin of the durophagous pelagic stingray ecomorph

**DOI:** 10.1111/pala.12669

**Published:** 2023-07-26

**Authors:** Giuseppe Marramà, Eduardo Villalobos-Segura, Roberto Zorzin, Jürgen Kriwet, Giorgio Carnevale

**Affiliations:** 1 Dipartimento di Scienze della Terra, Università degli Studi di Torino, Via Valperga Caluso 35, 10125 Turin, Italy; 2 Department of Palaeontology, University of Vienna, Josef-Holaubek-Platz 2, 1090 Vienna, Austria; 3 Museo Civico di Storia Naturale di Verona, Lungadige Porta Vittoria 9, 37129 Verona, Italy

**Keywords:** durophagy, ecomorph, evolution, Myliobatiformes, pelagic lifestyle, stingray

## Abstract

Studies of the origin of evolutionary novelties (novel traits, feeding modes, behaviours, ecological niches, etc.) have considered a number of taxa experimenting with new body plans, allowing them to occupy new habitats and exploit new trophic resources. In the marine realm, colonization of pelagic environments by marine fishes occurred recurrently through time. Stingrays (Myliobatiformes) are a diverse clade of batoid fishes commonly known to possess venomous tail stings. Current hypotheses suggest that stingrays experimented with a transition from a benthic to a pelagic/benthopelagic habitat coupled with a transition from a non-durophagous diet to extreme durophagy. However, there is no study detailing macroevolutionary patterns to understand how and when habitat shift and feeding specialization arose along their evolutionary history. A new exquisitely preserved fossil stingray from the Eocene Konservat-Lagerstätte of Bolca (Italy) exhibits a unique mosaic of plesiomorphic features of the rajobenthic ecomorph, and derived traits of aquilopelagic taxa, that helps to clarify the evolutionary origin of durophagy and pelagic lifestyle in stingrays. A scenario of early evolution of the aquilopelagic ecomorph is proposed based on new data, and the possible adaptive meaning of the observed evolutionary changes is discussed. The body plan of †*Dasyomyliobatis thomyorkei* gen. et sp. nov. is intermediate between the rajobenthic and more derived aquilopelagic stingrays, supporting its stem phylogenetic position and the hypothesis that the aquilopelagic body plan arose in association with the evolution of durophagy and pelagic lifestyle from a benthic, soft-prey feeder ancestor.

Batoids are a diverse group of cartilaginous fishes whose body plan (dorsoventrally compressed body, pectoral fins extending anteriorly and fused to the head forming a pectoral disc) is adapted for a benthic lifestyle (Aschliman *et al*. 2012a; Last *et al*. 2016; Villalobos-Segura *et al*. 2022). While guitarfishes (Rhinopristiformes) and electric rays (Torpediniformes) use their tail for locomotion, skates (Rajiformes) and stingrays (Myliobatiformes) use their pectoral fins for both locomotion and feeding (Mulvany & Motta 2014).

Stingrays show the highest morphological disparity (Martinez *et al*. 2016); most species possess a rounded to rhombic, soft and flexible pectoral disc supported by radials with four-chain catenated calcification that allow undulatory swimming in which multiple waves propagate along the pectoral-fin margin (Schaefer & Summers 2005). These features, coupled with a mostly negative fin-ray distribution index (FRD; Hall *et al*. 2018) and low pectoral-fin aspect ratio (AR; Martinez *et al*. 2016), are particularly efficient for swimming at low speed above the bottom (Rosenberger 2001; Schaefer & Summers 2005). These stingrays also use their pectoral disc to constrain and press prey against the substrate (tenting behaviour; Wilga *et al*. 2012; Hall *et al*. 2018). Prey are then grasped and processed through batteries of numerous, small, holaulacorhizous teeth of orthodont or osteodont histotype, forming the so called crushing-type dentition (Herman *et al*. 1998, 1999; Cappetta 2012; Underwood *et al*. 2015). This feeding apparatus is not designed for durophagy (specializations for crushing or cracking hard prey) but allows benthic stingrays to grasp, suck and chew, giving them the ability to consume a large variety of soft prey mainly including bony fishes, annelids and thin-shelled crustaceans (Summers 2000; Jacobsen & Bennett 2013; Kolmann *et al*. 2016). This body plan represents the generalized ‘rajobenthic’ ecomorphotype *sensu* Compagno (1990), making benthic soft-prey feeder stingrays among the most successful colonizers of shallow-water habitats in freshwater (Potamotrygoninae) and marine environments (Styracurinae, Dasyatidae, Urolophidae, Urotrygonidae, Plesiobatidae, Hexatrygonidae).

Another group of stingrays, including eagle rays (Myliobatidae, Aetobatidae), cownose (Rhinopteridae) and devil rays (Mobulidae), adopted a pelagic/benthopelagic lifestyle using an oscillatory swimming mode. They belong to the so called ‘aquilopelagic’ ecomorphotype *sensu* Compagno (1990), as they exhibit a different body plan, with head protruding anterior to a pectoral disc formed by laterally expanded wing-like fins resulting in high AR, stiffened by radials with crustal calcification and cross-bracing, and always having a positive FRD and often a *compagibus laminam*. This combination of features reduces drag whilst increasing lift and thrust generation, allowing active underwater flight in pelagic/benthopelagic environments (Schaefer & Summers 2005; Fontanella *et al*. 2013; Martinez *et al*. 2016; Hall *et al*. 2018). It has been suggested that stiffening pectoral fins for pelagic cruising represented a disadvantage in feeding efficiency as the fins lost the ability to manipulate and press prey against the substrate (Hall *et al*. 2018). However, aquilopelagic stingrays possess cephalic lobes, a pair of anterior appendages derived from the anteriormost propterygial segments, used to locate (with electro-receptive ampullae of Lorenzini) and manipulate prey (Mulvany & Motta 2014; Hall *et al*. 2018; Swenson *et al*. 2018). Therefore, while rajobenthic stingrays use their pectoral disc for both locomotion and feeding, aquilopelagic taxa evolved functional separation of these roles, with manipulation being assumed by cephalic lobes (Mulvany & Motta 2013; Hall *et al*. 2018; Swenson *et al*. 2018). Habitat shift co-occurred along with a diet shift, as pelagic stingrays evolved a suite of specialization for ‘extreme durophagy’ (*sensu* Underwood *et al*. 2017) being capable of consuming hard-shelled invertebrates (mainly bivalves and gastropods; Jacobsen & Bennett 2013). The general evolution of dentition in this group toward extreme durophagy can be summarized as follows: differentiation of teeth from a crushing-type toward a grinding-type dentition *(sensu* Cappetta 2012) through the achievement of enlarged polyaulacorhizous teeth of ‘modified osteodont’ histotype which are tightly interlocked to each other in a reduced number of anteroposterior files in a pavement-like arrangement; jaw cartilage strengthened by internal trabeculae, multiple layers of calcified cartilage on jaw surface; a lever-system that amplifies the adductors’ force; and jaws fused at symphyses (Summers *et al*. 1998; Herman *et al*. 2000; Summers 2000; Cappetta 2012; Underwood *et al*. 2015; Clark *et al*. 2022). A further feeding shift occurred during the Palaeogene when some durophagous stingrays evolved planktivory, with a reduction in dentition (Adnet *et al*. 2012; Underwood *et al*. 2017).

Although several studies concur to suggest that aquilopelagic stingrays are a derived clade within Myliobatiformes (McEachran & Aschliman 2004; Claeson *et al*. 2010; Aschliman *et al*. 2012a), how and when the various habitat shift and feeding specializations of this group arose remain unclear, mostly because both living ecomorphs are represented by highly-derived taxa without indisputable ‘transitional forms’ in the fossil record. Here, we report an almost complete articulated and exquisitely preserved fossil stingray from the Ypresian Pesciara site of the Bolca Konservat-Lagerstätte (northeastern Italy) that shows a mosaic of ‘rajobenthic’ and ‘aquilopelagic’ features. The inclusion of this stingray along with living and fossil taxa under phylogenetic and geometric morphometric approaches demonstrates progressive transition from the non-durophagous rajobenthic towards the durophagous aquilopelagic ecomorphotype.

## Geological Setting

**Fig. 1:**
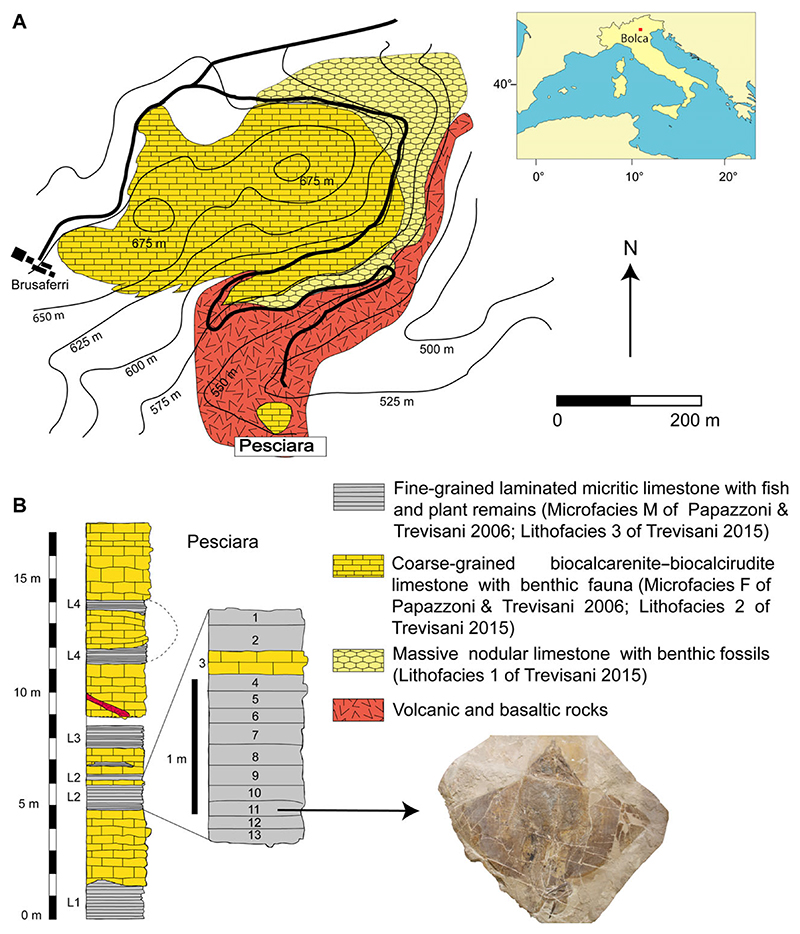
A, location and geological map of the Bolca area. B, stratigraphic section of the Pesciara sequence; the productive fossiliferous layers (2–12) investigated during the 2020–2021 controlled excavations are indicated; the new stingray herein described comes from the layer 11 of the fish-bearing level L2. Modified after Papazzoni & Trevisani (2006), and Trevisani (2015).

The specimen which is the focus of this study comes from the fossiliferous layers of the Pesciara site of the Bolca Konservat-Lagerstätte, located in the Lessini Mountains (southern Alps), about 2 km northeast of the village of Bolca, Verona Province, northeastern Italy (Fig. 1).

Several studies have referred the stratigraphic sequence of the Pesciara site to the ‘Calcari Nummulitici’, an informal unit of Eocene age widely distributed in northeastern (Italy) (Friedman & Carnevale 2018). The succession consists of a less than 20-m-thick cyclic alternation of finely laminated micritic limestones, with exquisitely preserved fishes, plants and invertebrates, and coarse-grained biocalcarenite/biocal-cirudite containing a rich benthic fauna. Its larger benthic foraminiferan content suggests that the fish-bearing limestone of the Pesciara site belongs to the *Alveolina dainelli* Zone or SBZ 11 Biozone, corresponding to the late Cuisian (late Ypresian, c. 50 Ma) (Papazzoni & Trevisani 2006; Papazzoni *et al*. 2014). Results of the quantitative palaeoecological study by Marramà *et al*. (2016a) allowed detailed definition of the palaeoecological and palaeoenvironmental setting of the Pesciara palaeobiotope, confirming that the Pesciara fish assemblage was characterized by a sharp oligarchic structure dominated by zooplanktivorous fishes, mostly clupeids (Marramà & Carnevale 2015; Marramà *et al*. 2016a). Taphonomic features and palaeoecological analyses confirm that the sediments were deposited in a shallow intraplatform basin (up to 50 m depth) in which benthic anoxic conditions and the development of a biofilm acted as promoters of the high-quality preservation of the fossils (Marramà *et al*. 2016a, 2021).

## Material and Method

### Terminology

Skeletal and tooth terminology primarily follow the recent literature (Nishida 1990; Carvalho *et al*. 2004; Aschliman *et al*. 2012b; Cappetta 2012). Fundamental terms are explained below.

#### Catenated calcification

Calcification pattern by which radials are incompletely covered by mineralized tesserae that form chains; there are two types of ‘catenated calcification’: in the ‘two-chain’ type, the tesserae form two chains on the dorsal and ventral surface of the radial; in the ‘four-chain’ type, the tesserae form four chains on the dorsal, ventral and lateral surfaces of the radial (Schaefer & Summers 2005).

#### Crushing-type dentition

Dentition formed by batteries of narrowly imbricated small teeth arranged in a high number of both anteroposterior files and mesiodistal rows in separate alternating rows often on highly-kinetic jaws unfused at the symphysis; the dental crown is usually bulging with often a transverse keel either smooth or with puckered or pitted enameloid; tooth batteries do not produce a flat surface, but rather an embossed surface (Cappetta 2012).

#### Crustal calcification

Calcification pattern by which radials are completely covered with a continuous and complete layer of mineralized tesserae (Schaefer & Summers 2005).

#### Grinding-type dentition

Dentition consisting of teeth with high crown and polygonal (tetragonal to hexagonal) outline; teeth are very narrowly imbricated forming robust dental plate with a nearly flat surface (pavement-like arrangement), useful for crushing hard prey with resistant shells (Cappetta 2012).

#### Compagibus laminam

A plate of skeletal elements on the anterior pectoral-fin edge that promotes lift during swimming (Hall *et al*. 2018).

#### Cross bracing

Interradial connections between individual radials showing lateral expansions at extremities that articulate with the surface of the adjacent radials (Schaefer & Summers 2005).

#### Crushing–grinding-type dentition

Dentition by which a moderate to marked monognathic heterodonty with some teeth files of crushing type, but the majority are of grinding type; dignathic heterodonty is marked by the grinding complex between teeth of pestles (curved upper lateral teeth) and teeth of crucibles (anterolateral lower teeth); whereas this mixing between crushing and grinding types is particularly notable in upper jaw, the lower jaw exhibits a more uniform tooth plate (Cappetta 2012; Adnet *et al*. 2019).

#### Holaulacorhizous

Teeth vascularized through a principal foramen in a median groove that divides the root in two distinct lobes (Cappetta 2012).

#### Modified osteodont histotype

Root lacks a pulp cavity and the whole crown is composed of osteodentine, which is crossed by irregularly shaped semi-parallel vascular canals that run mostly vertically (Herman *et al*. 2000).

#### Orthodont histotype

Tooth is supplied primarily by an internal pulp cavity radiating into numerous tiny canals penetrating the orthodentine layer (Herman *et al*. 1998, 1999).

#### Osteodont histotype

Tooth is supplied without any pulp cavity by scattered tiny cavities and canals penetrating the osteodentine layer of the root and the internal crown material (Herman *et al*. 1998, 1999).

#### Pectoral-fin aspect ratio (AR)

AR is an indicator of functionality whose values and variations in different taxa can be calculated to make inferences of potential swimming and lifestyle diversity and is strongly related to disc shape, swimming mode and environmental preferences. It is calculated as the maximum chord width of the pectoral-fin squared, divided by its surface area; the obtained value is therefore doubled for the single-calculated fin (Martinez *et al*. 2016).

#### Pectoral-fin-ray distribution index (FRD)

This index illustrates the evolutionary trend in the pectoral-fin-ray distribution, in which propterygial radials decrease in number with respect to the metapterygials in concomitance with a shift toward a pelagic lifestyle. The index is calculated as the number of metapterygial minus propterygial radials, divided by the total number of pectoral-fin radials (Hall *et al*. 2018).

#### Polyaulacorhizous

Teeth vascularized through several small foramina irregularly arranged at the bottom of the grooves which are separated by parallel laminae (Cappetta 2012).

### Fossil material

This study is based on a nearly complete and articulated fossil stingray specimen discovered in 2020 *in situ* at Pesciara site of the Bolca Konservat-Lagerstätte, during controlled excavations carried out by the Museo Civico di Storia Naturale di Verona (MCSNV), and then secured, collected and prepared by personnel of the same institution, where it was housed.

The 2020 palaeontological excavation campaign at the Pesciara site began on 28th December 2020, and lasted about one month. Controlled excavations involved about one cubic metre of rock from the eastern part of the tunnel penetrating the Pesciara outcrop and investigated the productive fossiliferous strata from 2 to 12 of the second fish-bearing level (L2 of Papazzoni & Trevisani 2006) (Fig. 1B). The slabs extracted were enumerated following the layers’ numbering and subsequently opened using small hammers and chisels. The fossil stingray lay on layer 11, inclined about 25°, with its body anteroposteriorly oriented in the direction 346°N. This orientation has been also observed in other fossils, albeit with small variations, which were found in layers 1B, 2T and 10. During the extraction, the slabs containing the stingray fractured into numerous blocks up to 15 cm thick. In the laboratory, the various blocks were assembled using glue, the thickest slabs were thinned, and the most fragile slabs were consolidated. At the end of the excavation campaign, a column and a beam of steel-reinforced concrete were cast in order to make the extraction area safe.

The fossil specimen, preserved in two limestone slabs as part (MCSNV VR.21.107) and counterpart (MCSNV VR.21.108), was directly investigated with hand lenses and macrophotography. Some teeth and placoid scales were extracted and examined using a Leica M80 stereomicroscope. Optical photographs were obtained using a Canon EOS 600D camera coupled with different macro lens (Canon EF 50 mm 1:2.5; Tamron AF 90 mm F/2.8). Anatomical details were also examined under ultraviolet light to better distinguish preserved skeletal elements and soft tissues. The analysis with UV-induced visible fluorescence was performed by means of preliminary direct observations and subsequent photographs. Both slabs were exposed to a Bresciani Led Spotlight 1Wave 3W (UV-A; emission peak at 365 nm).

Measurements were taken to the nearest 0.1 mm, and body proportions are calculated based on disc width (DW). The von Bertalanffy growth equation was employed to calculate the estimate age for the holotype of †*Dasyomyliobatis thomyorkei* following the methods applied in studies on living and fossil chondrichthyans, and stingrays in particular (Smith & Merriner 1987; Dubick 2000; Yeldan *et al*. 2009; Yigin & Ismen 2012; Başusta & Aslan 2018; Marramà *et al*. 2018): *t*_1_ = (1/*k*) × ln(*maxDW*/(*maxDW* − *DWt*)) + *t*_0_, where *t*_1_ is the age of the individual (in years), *k* is the growth coefficient (rate of change in length increment), *DWt* is the disc width of the individual (in cm), *maxDW* is the mean maximum disc width for the population (in cm), *t*_0_ is the hypothetical postnatal age extrapolated from a growth curve when length equals zero. We substituted the parameters *k*, *maxDW* and *t*_0_ with values taken from growth curves of multiple living stingray populations mostly composed of female individuals (see Table S1) since our fossil stingray is thought to be a female individual based on the absence of claspers (see Description). These calculations provide a range of numerical estimate (uncertainty for the estimates are provided as standard errors). For example, a dataset for females of *Myliobatis californica* (Martin & Cailliet 1988) has the following life history parameters: *k* = 0.0995, *t*_0_ = –2.06 (years), *maxDW* = 158.7 (cm). As *DWt* = 99.9 (cm) for MCSNV VR.21.107/8, the estimated age of the individual is: *t*_1_ = (1/0.0995) × ln(158.7/(158.7–99.9)) – 2.06 = 7.92 ± 1.5 years. The palaeobiological implications of the age estimates for †*Dasyomyliobatis thomyorkei* are based on the comparison of these values with the age at maturity of living stingray species to predict whether the fossil specimen is likely to represent a sexually mature or an immature individual. In the former example, assuming that †D. *thomyorkei* grew following the von Bertalanffy function of M. *californica* (therefore predicting that †D. *thomyorkei* reached maturity at 5.0 years), an estimate age 7.92 ± 1.5 years for MCSNV VR.21.107/8 indicates that it was a mature (adult) individual.

### Phylogenetic analysis

In order to estimate its phylogenetic relationships and track the evolutionary trajectories giving rise to durophagy and pelagic lifestyle in Myliobatiformes, we performed phylogenetic analyses employing parsimony, Bayesian and maximum likelihood (ML) approaches (Marramà *et al*. 2023). The morphological dataset used for our phylogenetic analyses was assembled using different sources (Carvalho *et al*. 2004; Claeson *et al*. 2010; Underwood *et al*. 2017; Marramà *et al*. 2019a, 2020) and implemented with seven additional characters described herein for the first time (for character list, see Appendix S1). The matrix was compiled in Mesquite v.3.03 (Maddison & Maddison 2008) following the logical bases underlying characters and character states in phylogenetic analyses proposed by Sereno (2008), avoiding inappropriate mixing of neomorphic (absence/presence) and transformational (qualitative or quantitative) character statements. We also modified and split some character statements originally proposed by Claeson *et al*. (2010) in order to detect the first appearance of the derived conditions in both the symphyseal and lateral teeth (see our characters 49, 50, 62–67). The matrix resulted in the most comprehensive and largest dataset on stingrays published so far, including 124 characters and 52 taxa, these latter including four outgroups and 48 living and fossil stingrays. Fossils include both holomorphic taxa (represented by nearly complete and articulated specimens) and tooth-based taxa (those represented in the fossil record by isolated teeth or dental plates only). Our comprehensive matrix also benefited from personal observations for taxa listed in Appendix S2.

To detect which traits characterized the shift toward the durophagy and pelagic lifestyle we performed two different phylogenetic analyses: the first one includes all 52 living and fossil stingrays (both holomorphic and tooth-based taxa) and is useful to detect the evolutionary trajectories toward the stingray durophagy, since the inclusion of fossil tooth-based taxa allows us to recover the evolution of dental traits along the nodes. A second analysis to detect the transition from undulatory to oscillatory swimming, and consequent habitat shift from benthic toward pelagic environments, required tooth-based taxa to be removed from the analysis, in order to detect which traits related to the pectoral-fin morphology characterize each node. Conversely, traits related to pectoral skeleton cannot be detected on several nodes if tooth-based fossil taxa were kept, because these characters are coded as unknown (?) in the data matrix for these taxa. For the latter analysis, only 40 holomorphic living and fossil taxa were kept. All 124 characters were used in both analyses.

Parsimony analyses were performed with TNT v.1.5 (Goloboff & Catalano 2016) using the heuristic search method with 1000 random addition sequences followed by branch-swapping using the tree bisection and reconnection (TBR) algorithm, saving up to ten trees per replication (best score hit 3 times out of 10; best score (TBR): 312; 1 tree retained). All characters are unordered and given equal weight. Using the same data matrix, we also performed Bayesian and ML analyses, to crosscheck and test the robustness of the results obtained from parsimony analyses. The ML analysis was carried out in PAUP v.4 (Swofford 2003) employing the Mkv model (Markov K model for discrete morphological data with only variable characters), with the TBR algorithm for branch permutations with 10 000 replications, estimating a gamma distribution across characters, using a neighbour joining tree as starting point. The Bayesian analysis was performed in MrBayes v.3.2.7a (Ronquist *et al*. 2012) under a Mkv model with gamma-distributed rates. This analysis combined the results of two independent runs, each employing four chains, and the following search options where used: ngen = 195 500 000, samplefreq = 500 000, printfr = 10 000, diagnfreq = 500 000, nruns = 2, checkfreq = 500 000, nchain = 4, temp = 0.03, stopval = 0.01, stoprule = yes, nperts = 2, savebrlens = yes, with a burnin fraction (discarded trees) of 25%. The Bayesian analysis ran over 195 500 000 generations reaching an average standard deviation of split frequencies of 0.017620 with a potential scale reduction factor of between 0.9 and 1.2, and an average estimated sample size <100 suggesting that convergence between the chains occurred (Hall 2011). The effective sample size was higher than 200 and was checked in Tracer v.1.7.1 (Rambaut *et al*. 2018).

### Time scaling

The tree topologies were time-scaled using an *a posteriori* approach using the equal method, following, amongst others, Ruta *et al*. (2006) and Brusatte *et al*. (2008). This approach takes a basic scaled tree earlier and shifts root age by some constant (rlen = 10) and then equally reapportions the duration of non–zero length internode edges along successive zero-length child edges. The time scaling of the phylogenetic trees was carried out using the function ‘StratPhyloCongruence’ of the R package Strap v.1.6-0 (Bell & Lloyd 2015); 1000 random trees were estimated from the initial tree with the ages for the taxa being sampled form the known fossil record of the species using the first appearance datum (FAD) and last appearance datum (LAD) (Table S2). The relative completeness index (RCI) (Benton & Storrs 1994) was used to evaluate the trees and the tree with the best RCI was used as indicates the least incomplete fossil record.

### Morphospace analysis

As theoretical and experimental evidence suggests an unquestionable relationship between pectoral-disc shape, functionality and lifestyle in batoids (Fontanella *et al*. 2013), and since †*Dasyomyliobatis* has a unique structure of its pectoral disc which is not present in any other stingray, we used geometric morphometrics to analyse the morphospace occupied by living and fossil holomorphic stingrays and to infer lifestyle and swimming mode.

#### Taxon sampling

To analyse the morphospace occupation of fossil and Recent stingrays, the genus (not species) was used as main standard unit, being considered as a reliable taxonomic rank for analysing biological and morphological diversity (Forey *et al*. 2004; Marramà *et al*. 2016b, 2016c). Morphospace analyses was performed at the genus level using a single species as representative of the body plan since stingrays usually show a low degree of morphological variation within the genus (Last *et al*. 2016). Exceptions were *Myliobatis* and *Mobula*, as we used four and two different species for each genus, respectively. Images of 35 species (28 extant and 7 holomorphic fossils) were obtained from different sources, including photos taken from museum specimens, literature and online picture repositories (see Appendix S2).

#### Geometric morphometrics protocols

Body shape and occupation of the different ecomorphotypes were studied through landmark-based geometric morphometric method (Zelditch *et al*. 2004). Following the scheme applied in the most reliable study about the pectoral-disc shape variation in batoid fishes (Martinez *et al*. 2016) a total of 33 sliding semilandmarks and two fixed landmarks (one on each end point of the sliding-landmark curve) were placed along the outer margin of the left pectoral fin using the software package TPSdig v.2.05 (Rohlf 2005). The landmark coordinates were translated, rotated and scaled at unit centroid size by applying a generalized Procrustes analysis (GPA) to minimize the variation caused by size, orientation, location and rotation (Zelditch *et al*. 2004). The new landmark coordinates (Procrustes coordinates) represent new shape variables, which are decomposed into uniform and partial warp components. Principal component analysis (PCA) was then performed on Procrustes coordinates to obtain the relative warp (RW). GPA and PCA were performed using the TPSrelw software package (Rohlf 2003). RWs (= PCs) are vectors describing the maximum variation of specimen shape compared to the consensus configuration (mean shape). The two-dimensional morphospace of each ecomorphotype was defined using the area inside the convex hull (i.e. the minimum convex polygon enclosing all specimens) built on PC axes explaining over 5% of the variance (Zelditch *et al*. 2004). In our case, only the first two PCs explain more than 5% of total variance. Changes in shape along these axes are visualized through deformation grid plots.

#### Patterns of morphospace occupation

To assess significant differences in morphospace occupation among stingray ecomorphotypes we used two non-parametric tests. The non-parametric multivariate analysis of variance (PERMANOVA; Anderson 2008) and the analysis of similarities (ANOSIM; Clarke 1993) were performed to assess significant differences in morphospace occupation between groups. PERMANOVA was applied to detect significant differences in group centroid position, whereas ANOSIM was employed to test for significant overlap between convex hulls. Statistical significance was calculated using all PCs with 9999 random permutations. Euclidean distances were chosen as distance measure and the Bonferroni correction was applied for both tests. PERMANOVA and ANOSIM were performed using PAST v.4.10 (Hammer *et al*. 2001).

### Functional implications of pectoral shape

As AR is an indicator of functionality (its values and variations in different taxa can be calculated to make inferences of potential swimming and lifestyle diversity; Martinez *et al*. 2016), we also investigated the relationship between pectoral-fin shape and AR through linear regression in order to detect functional implications of pectoral shape of †*Dasyomyliobatis*. AR is calculated as the maximum chord width of the pectoral-fin squared, divided by its surface area; the obtained value is therefore doubled for the single-calculated fin to get the estimated AR value for both fins (Martinez *et al*. 2016). In our study, maximum chord and surface area for the living and holomorphic fossil taxa were obtained using the software package ImageJ on the images used for geometric morphometrics (see Table S3). AR is strongly related to disc shape, swimming mode and environmental preferences: low AR (<1.0) is related to species with rounded pectoral-fin outlines, their bodies being suited for fine-scale manoeuvrability within their riverine and marine environments (e.g. Potamotrygonidae); benthic demersal stingrays with subcircular to subrhombic pectoral-disc shape have mid–low ratio (1.0 < AR < 2.0), whereas stingrays with a pelagic or benthopelagic lifestyle and crescent-shaped pectoral-fin with a convex leading edge and concave trailing edge are characterized by very high AR (>3.0). An intermediate ratio (2.0 < AR < 3.0) is typical of taxa like *Gymnura*, a peculiar stingray closely related to some benthic stingrays (Hexatrygonidae, Urolophidae, Plesiobatidae) but sharing some myliobatoid features (e.g. wing-like fins stiffened by crustal-calcified radials and cross-braces).

As the aspect ratio strongly related to disc shape, swimming mode and environmental preferences, AR is used to discriminate the two main stingray ecomorphotypes (Martinez *et al*. 2016): the rajobenthic ecomorph (rajomorph of Martinez *et al*. 2016) is represented by stingrays with AR < 2.0; the aquilopelagic ecomorph (pelagomorph of Martinez *et al*. 2016) is represented by stingrays having AR > 3.0. We introduce hereafter the ‘aquilobenthic ecomorph’ for those stingrays characterized by intermediate values (2.0 < AR < 3.0) and hybrid body traits and lifestyles (see below).

## Results

**Fig. 2:**
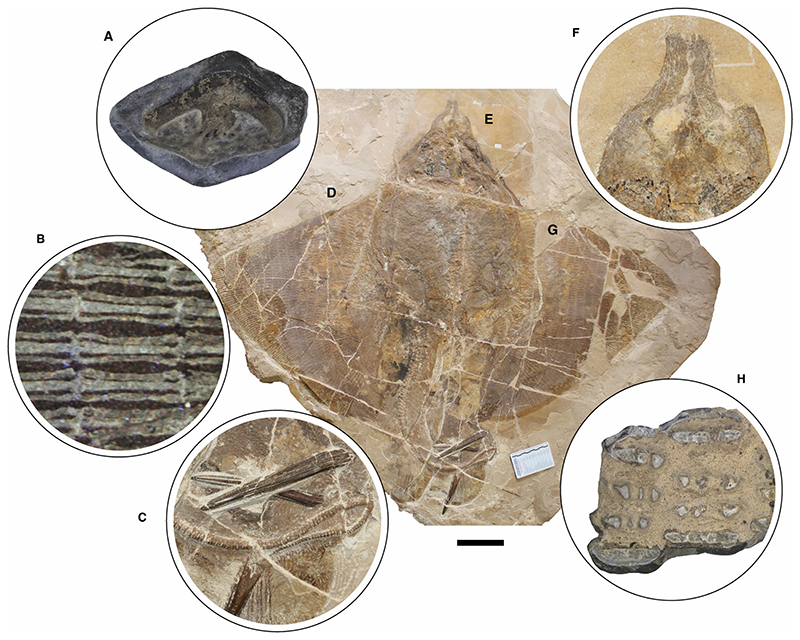
†*Dasyomyliobatis thomyorkei* gen. et sp. nov. (MCSNV VR.21.107, holotype) showing its peculiar combination of rajobenthic (A–D) and aquilopelagic (E–H) traits: A, small holaulacorhizous lateral teeth arranged in alternating rows; B, pectoral-fin radials with catenated calcification and no cross-bracing; C, free tail vertebrae without cartilaginous rod and caudal fin reduced to a ventral fold; D, soft, flexible pectoral disc with convex anterior and posterior margins; E, head protruding from pectoral disc; F, cephalic lobes; G, wing-like pectoral disc with positive FRD; H, enlarged hexagonal symphyseal/parasymphyseal polyaulacorhizous teeth in pavementlike arrangement. Scale bar represents 100 mm.

The morphological analysis of this exquisitely preserved and articulated fossil specimen has revealed the existence of a new genus and species of stingray in the Eocene Konservat-Lagerstätte of Bolca, namely †*Dasyomyliobatis thomyorkei*, which represents a new stingray family, the †Dasyomyliobatidae (Fig. 2). Although numerous characters clearly support the inclusion of †*Dasyomyliobatis* within the Myliobatiformes (e.g. inconspicuous rostral cartilage, venomous tail stings and thoracolumbar synarcual; Compagno 1973; Lovejoy 1996; Carvalho *et al*. 2004; McEachran & Aschliman 2004), †*Dasyomyliobatis* possesses a unique mosaic of plesiomorphic traits typical of benthic soft-prey feeder stingrays (the rajobenthic ecomorph) and derived characters typical of durophagous pelagic stingrays (the aquilopelagic ecomorph), which have never been found in any fossil or living stingray (Fig. 2). Like rajobenthic stingrays, †*Dasyomyliobatis* has a soft and flexible pectoral disc supported by catenated radials with no cross-bracing that would have allowed undulatory swimming, numerous batteries of labiolingually directed files of small holaulacorhizous lateral teeth arranged in alternating rows, tail formed by free vertebrae not stiffened by a cartilaginous rod, and a caudal fin reduced to a ventral fold. At the same time, like the aquilopelagic stingrays, †*Dasyomyliobatis* has a head protruding anterior to a wing-like pectoral disc with positive FRD, cephalic lobes, hexagonal and polyaulacorhizous symphyseal and parasymphyseal teeth in pavement-like arrangement with bulbous/irregular interlocking mechanism.

The peculiar combination of two types of teeth is characteristics of the crushing–grinding type, an intermediate dentition characterized by moderate to marked monognathic heterodonty with some teeth files of crushing type, and the majority of grinding type (Cappetta 2012; Adnet *et al*. 2019). Dignathic heterodonty is marked by the grinding complex between teeth of pestles (curved upper lateral teeth) and teeth of crucibles (anterolateral lower teeth). Crushing–grinding dentition has also been observed in fossil and living *Pastinachus* species, and Late Cretaceous to early Palaeogene taxa with holaulacorhizous teeth only, or with both holaulacorhizous and polyaulacorhizous teeth, including †*Hypolophites*, †*Brachyrhizodus*, †*Meridiania*, †*Hypolophodon*, †*Myliodasyatis* (Cappetta 2012; Adnet *et al*. 2019).

### Detecting the origin of durophagy

**Fig. 3:**
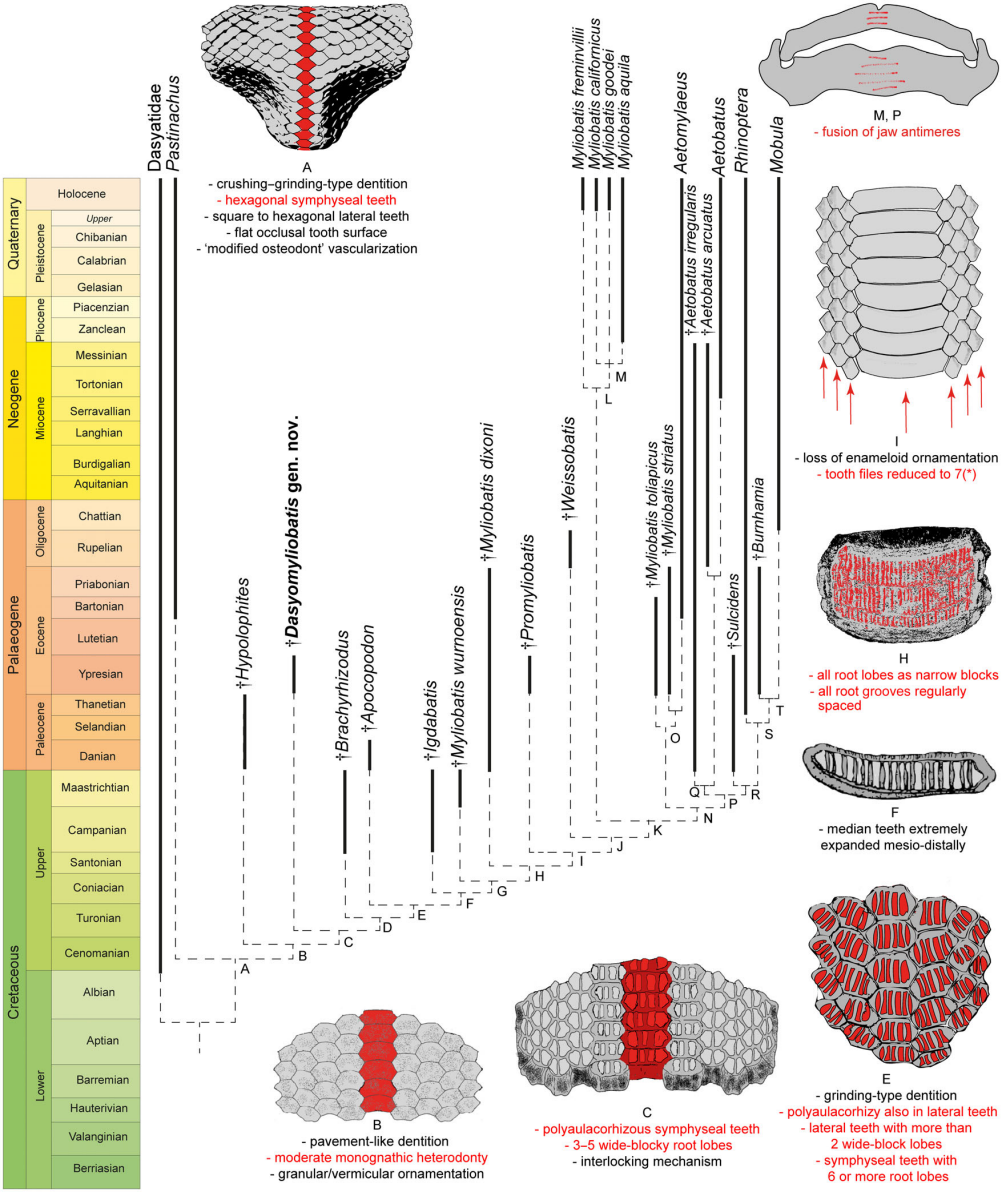
The simplified single time-calibrated tree recovered from parsimony analysis of 124 characters and 52 taxa and the unambiguously optimized character states that support the nodes (see also Figs S1, S4). The other dental/jaw characters that support nodes are: D: domed crown (61[1]); G: interlocking tongue from bulbous to short shelf (58[0 > 1]); J: non-dental characters; K: loss of upper tooth curvature (55[1 > 0]); L: direction of tooth curvature flat/horizontal (56[1]); N: non-dental characters; O: crown shape deep in anterior or posterior view (61[2]); P: root lobes in symphyseal teeth as fine edges (66[3]); Q: symphyseal teeth rectangular with posteriorly deflected lateral margins (49[2]); lateral teeth absent (51[1]); strongly curved lower teeth (54[2]); interlocking tongue as long shelf (58[2]); low crown (59[1]): roots long and strongly inclined (69[2]); single file of teeth (118[3]); R: root grooves wider than laminae (68[1]); S: some lateral teeth very expanded (53[1]); interlocking tongue bulbous/irregular (58[0]); 19–8 tooth files on jaws (117 [1]); T: tooth curvature flat/horizontal (56[1]); reduction in jaw trabeculation and surface mineralization (105[1]; 108[1]). * detected on node C of Figure 4. Red text corresponds to the illustrated red-coloured characters.

Parsimony analysis including †*Dasyomyliobatis* along with living and fossil (holomorphic and tooth-based) taxa produced a single tree (312 steps, CI 0.538, RI 0.847) simplified in Figure 3 (see also Fig. S1). The resulted topology shows that durophagy in stingrays is likely to have arisen in a group of benthic soft-prey feeders of the family Dasyatidae, the Hypolophinae (here represented by *Pastinachus* and †*Hypolophites*) that already possess some specializations for crushing or cracking hard prey (Adnet *et al*. 2019), including hexagonal symphyseal teeth (49 [1]); lateral teeth square to hexagonal, depending on tooth position (50[1]), and flat occlusal tooth surfaces (60[1]) forming a crushing–grinding type dentition (124 [1]); lower jaw profile strongly expanded linguolabially (106[1]); and tooth histotype shifting from orthodont to ‘modified osteodont’ histotype (78[0 > 2]) (node A). Taxa on node B furtherly achieve pavement-like tooth arrangement (48[1]), moderate monognathic heterodonty (52[1]), and granular to vermicular enameloid ornamentation (120[1]). *Pastinachus* and †*Hypolophites* are successive sister taxa to †*Dasyomyliobatis* plus the remaining stingrays (excluding Dasyatidae). Four characters support this grouping (node C): presence of tooth interlocking mechanism (57[0 > 1]); polyaulacorhizous symphyseal teeth (62[0 > 1]) with 3–5 root lobes (64[0 > 1]) being wide-block in basal view (66[0 > 1]). Taxa on node D achieve a domed crown shape (61[1]), which is also present in the earliest myliobatids such as †*Apocopodon*, †*Igdabatis* and †*Myliobatis wurnoensis* (Claeson *et al*. 2010; Santana *et al*. 2011; Cappetta 2012; Blanco 2019). Complete polyaulacorhizy is achieved on node E when lateral teeth also become polyaulacorhizous (63[0 > 1]); root-lobes increase in number both in symphyseal (six or more; 64[1 > 2]); and lateral teeth (65[0 > 1]), form wide blocks (67[0 > 1]), becoming grinding-type (123 [1 > 2]). †*Apocopodon* and †*Igdabatis* are successive sister taxa to the remaining, more derived, stingrays. The next steps toward extreme durophagy are related to the appearance of strong monognathic heterodonty (52 [1 > 2]; node F) as median teeth become extremely mesiodistally enlarged; the shape of the tongue passes from bulbous or irregular to short-shelf (58[0 > 1]; node G); root lobes change from wide-block-like to narrow-block-like in both symphyseal and lateral teeth (66 [1 > 2]; 67[1 > 2]); and root grooves pass from irregularly to regularly spaced (70[1 > 0]) (node H); taxa on node I (†*Promyliobatis* plus remaining stingrays) show a reduction of anteroposterior files to seven (118[2]) and loss of ornamentation (119[0]).

Bayesian and ML analyses recovered the same topologies of the evolutionary steps giving rise to aquilopelagic stingrays since *Pastinachus*, †*Hypolophites*, †*Dasyomyliobatis*, †*Brachyrhizodus*, †*Apocopodon*, †*Igdabatis*, †*Myliobatis wurnoensis*, †*M*. *dixoni*, †*Promyliobatis* and †*Weissobatis* are successive sister taxa to all remaining aquilopelagic stingrays (Fig. S3) suggesting that the characters employed in the analyses are robust, that the resulting systematic arrangement is very stable, and that the hypothesis of gradual achievement of traits supporting the evolutionary origin of the durophagous pelagic ecomorph appears reliable. Differences between tree topologies concern the different position of some taxa, making Dasyatidae, Potamotrygonidae, Urolophidae and *Myliobatis* paraphyletic in Bayesian and/or ML analyses. Details on tree topologies are presented in Appendix S3.

### Detecting the origin of the pelagic lifestyle

**Fig. 4:**
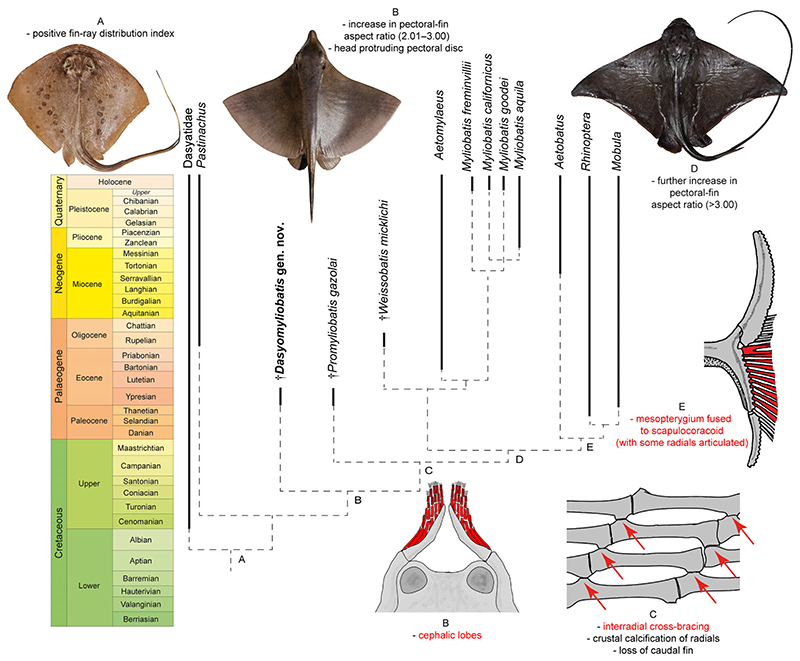
The simplified time-calibrated 50% majority rule tree recovered from parsimony analysis of 124 characters and 40 taxa (holomorphic only) and the unambiguously optimized character states that support the nodes (see also Figs S2, S5). All the other nodes without capital letters are supported by dental characters. Red text corresponds to the illustrated red-coloured characters. Artwork of †*Dasyomyliobatis* by Fabrizio Lavezzi.

The parsimony analysis including living and holomorphic fossil taxa (i.e. excluding those based on teeth only) produced three equally parsimonious trees that were used to build the 50% majority rule tree (286 steps, CI 0.570, RI 0.831) simplified in Figure 4 (see also Fig. S2). The tree reflects a similar topology to that depicted in Figure 3 showing a gradual achievement of traits towards a complete pelagic/benthopelagic lifestyle. Taxa on node A show positive FRD suggesting that dasyatids already presented a reduction tendency in the number of propterygial radials (further reduced in aquilopelagic taxa). Node B, including †*Dasyomyliobatis* as sister to all aquilopelagic stingrays, is supported by the appearance of cephalic lobes (45[1]) and an increase in AR, with an intermediate value between those of rajobenthic and aquilopelagic stingrays (115[1 > 2]). Although the head already extends anterior to the pectoral disc in †*Dasyomyliobatis*, this state (116 [0]) is not recovered at the node, probably because it is not present in †*Promyliobatis*, in which the head rests inside the pectoral disc, possibly representing a reversal. The following step (node C, including †*Promyliobatis* as sister to remaining stingrays) defines achievement of the crustal calcification (110[1]) coupled with cross-bracing (30[1]), and loss of the caudal fin (36[1]). All remaining stingrays (node D) achieve the final and definitive crescent pectoral-disc shape, highlighted by an increase in AR (>3.0; 115[2 > 3]).

### Body shape analysis

**Fig. 5:**
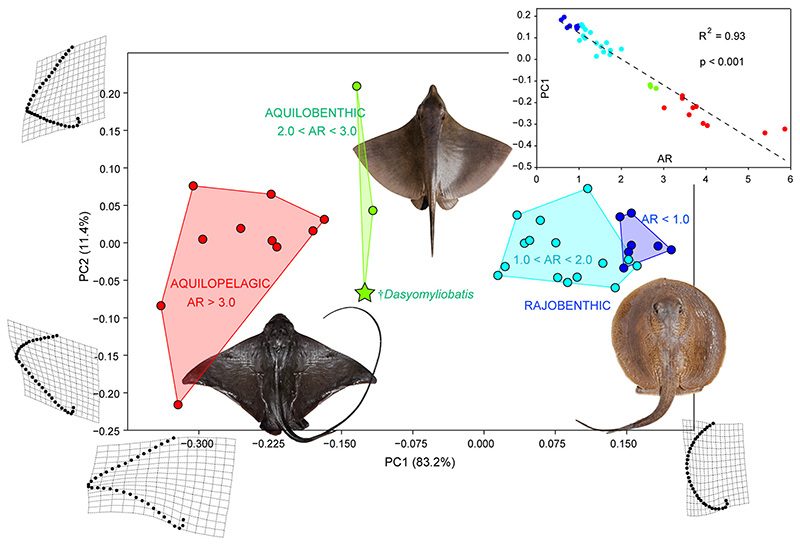
Morphospace of recent and fossil holomorphic stingrays grouped based on their pectoral-fin aspect ratio, showing significant separation of the main three ecomorphs (rajobenthic, aquilobenthic, aquilopelagic) based on their habitat preferences and swimming mode (p < 0.001; see Table S4). Changes in shape along the axes are visualized through deformation grids. The upper right quadrant illustrates the regression of PC1 on aspect ratio (AR) showing significant relationship between the two proxies, suggesting significant relationship between shape and swimming/habitat preferences (p < 0.001). Artwork of †*Dasyomyliobatis* by Fabrizio Lavezzi.

The geometric morphometrics performed on pectoral-fin shape detected 34 principal components (PCs). The first two PCs account for 94.58% of total variation (Fig. 5). PC1 (83.19%) describes the variation from wing-like shapes with concave posterior margin on negative scores (e.g. Mobulidae) to perfectly rounded shapes on positive values (e.g. Potamotrygonidae), while PC2 (11.39%) is associated with variation in the location of the pectoral-fin’s lateral apex dividing the fin into anterior and posterior regions. Taxa near the consensus shapes are represented by stingrays with roughly equilateral triangular fins with convex anterior and posterior margins (e.g. majority of Dasyatidae).

The rajobenthic ecomorphotype (AR < 2.0) lies entirely on positive scores but substantial variation can be detected within this group suggesting significant diversity in locomotion, activity level, and lifestyle within this group alone. Species with AR < 1.0 and rounded bodies (Potamotrygonidae) have pectoral fins suited for fine-scale manoeuvrability within their riverine or marine environments, whereas stingrays like dasyatids with rhombic-shaped fins (1.0 < AR < 2.0) are more vagile, undergoing seasonal migrations (Last *et al*. 2016; Martinez *et al*. 2016). The aquilopelagic ecomorphotype (AR > 3.0) lies on negative scores: extreme negative values are occupied by pelagic mobulids, whereas benthopelagic taxa like *Myliobatis* lie on higher values, reflecting different shapes and habitat preferences. The convex hull including taxa with 2.0 < AR < 3.0 (*Gymnura*, †*Dasyomyliobatis*, †*Promyliobatis*) lies in an intermediate position, reflecting hybrid shape, swimming and habitat preferences (Rosenberger 2001; Martinez *et al*. 2016). We therefore propose a new ecomorphotype, aquilobenthic, to better reflect this hybrid ecomorph for those stingrays characterized by intermediate values (2.0 < AR < 3.0), hybrid body traits, swimming and/or lifestyles. Regression of PC1 on AR (Fig. 5) shows highly significant relationship between shape and swimming/habitat preferences (R^2^ = 0.93; p < 0.001).

Stingrays are clustered in three distinct groups along PC1 based on their AR, and therefore based on their habitat preferences and swimming mode. The PERMANOVA performed on all PC axes rejects the null hypothesis of similarity of group centroids between different ecomorphs (p = 0.0001) whereas the ANOSIM rejects the null hypothesis of equal median and range values for within-group ranked dissimilarities among the ecomorphotypes’ convex hulls (p = 0.0001; Table S4) therefore supporting the hypothesis that these groups occupy different morphospaces and, possibly, ecological niches.

## Discussion

### Origin of durophagy

It is widely accepted that the evolution of the durophagy involved differentiation of teeth from crushing-type toward grinding-type through tooth broadening, progressive reduction of the enameloid, reduction in anteroposterior file number, predominance of hexagonal symphyseal teeth, and achievement of polyaulacorhizy (Summers 2000; Cappetta 2012).

Our analysis (Fig. 3) shows that the first step in the evolution of durophagy was the shift from a crushing-type dentition (rounded to rhombic cusped crown having orthodont or osteodont histotype) to an intermediate crushing–grinding type dentition (teeth with high and flat crown, rhombic to hexagonal outline, and ‘modified osteodont’ histotype). In crushing–grinding dentitions, teeth are still minute (up to 10 mm) or medium sized (10–25 mm in †*Brachyrhizodus*), narrowly imbricated but forming robust lower dental plate with a nearly flat surface (Cappetta 2012). Crushing–grinding type dentitions are typical of taxa with full holaulacorhizy (e.g. *Pastinachus*, †*Hypolophites*) and stingrays with polyaulacorhizy on median teeth only (e.g. †*Dasyomyliobatis, †Brachyrhizodus)*. In these latter, dentition is characterized by moderate monognathic heterodonty and polyaulacorhizous teeth with 3–5 irregularly spaced and wide-blocky root laminae. Crushing–grinding dentition allowed these taxa to feed not only soft-prey (bony fishes, worms, thin-shelled crustaceans) but also to crush hard-shelled molluscs (Underwood *et al*. 2017; Maisch 2019), as in living *Pastinachus* (Devadoss 1978; Jacobsen & Bennett 2013; Rastgoo *et al*. 2018). As these stingrays still do not possess the whole mosaic of specialization for ‘extreme durophagy’, we can define a ‘moderate’ degree of durophagy (or moderate durophagy) to distinguish them from those stingrays achieving complete polyaulacorhizy, full grinding-type dentition with strong monognathic heterodonty, reduction of anteroposterior files to seven, internal trabeculae, multilayered calcified cartilage on jaw surface, lever-system that amplifies the adductors’ force, and symphysis fusion (e.g. Summers *et al*. 1998; Herman *et al*. 2000; Summers 2000; Cappetta 2012).

In †*Apocopodon*, †*Igdabatis* and †*Myliobatis wurnoensis*, there are still wide-block laminae but their lobes become progressively more regularly spaced and numerous (six or more); teeth are always medium sized (10–25 mm), symphyseal teeth become wider and the dentition achieves stronger monognathic heterodonty and complete polyaulacorhizy (fully grinding-type). In more derived taxa, symphyseal teeth become larger (>100 mm), the number of files decreases progressively (up to seven in †*Promyliobatis*, †*Weissobatis*, *Myliobatis* and *Aetomylaeus*; one in *Aetobatus*) and enameloid ornamentation on the tooth surface is lost resulting in the achievement of extreme durophagy *(sensu* Underwood *et al*. 2017).

Our tree topology, with gradual achievement of traits towards extreme durophagy, fits well with the fossil record, as stingrays with complete or incomplete polyaulacorhizy, like †*Brachyrhizodus* and †*Igdabatis*, traditionally considered to be the oldest myliobatids, are Campanian in age, followed by the Maastrichtian †*Myliobatis wurnoensis*, and then by the early Palaeogene †*Apocopodon* (Claeson *et al*. 2010; Cappetta 2012; Blanco 2019).

Our time-calibrated phylogeny (Fig. 3; Fig. S4) corroborates the hypothesis that the most recent common ancestor of extant durophagous stingrays was a Late Cretaceous oscillator-swimmer with grinding dentition (Aschliman 2014) but also that the gradual achievement of traits started at least in the early Late Cretaceous, with the appearance of taxa with moderate degree of durophagy (crushing–grinding dentition, small–medium tooth size, high crown, incomplete polyaulacorhizy, low-moderate monognathic heterodonty). The earliest fossil genera showing a moderate degree of durophagy known to have both holaulacorhizous and polyaulacorhizous teeth (†*Dasyomyliobatis*, †*Brachyrhizodus*, †*Coupatezia*, †*Myliodasyatis*, †*Hypolophodon*, †*Heterobatis*, †*Meridiania*, †*Aturobatis*, †*Garabatis*, †*Eomobula;* Cappetta 2012) or polyaulacorhizous teeth only (e.g. †*Igdabatis*, †*Apocopodon*) are known from the Campanian to Eocene, co-existing (from Maastrichtian on) with stingrays with extreme durophagy (e.g. extinct ‘*Myliobatis*’ species). At the end of the Palaeogene, moderate durophagous taxa (except *Pastinachus*) were completely replaced by sting-rays with extreme durophagy.

Although Cappetta (2012) suggested that Myliobatidae is likely to have originated from Dasyatidae, he stated that *Pastinachus* or †*Hypolophites* are not direct ancestors of durophagous stingrays. Conversely, the present study suggests that Dasyatidae is sister to a clade that includes *Pastinachus*, †*Hypolophites*, †*Dasyomyliobatis* and †*Brachyrhizodus* as successive sister taxa to all remaining durophagous stingrays. This hypothesis is not so unlikely since living dasyatids like *Pastinachus* and *Urogymnus* often show aberrant polyaulacorhizous teeth with more than two root lobes (Herman *et al*. 1998, 2000; Adnet *et al*. 2019) resembling the condition of †*Dasyomyliobatis* and †*Brachyrhizodus*.

The generalized dasyatoid jaw and ‘intermediate’ tooth morphology of †*Dasyomyliobatis*, suggest that it was a stingray with moderate durophagy capable of feeding on a wide range of prey items, from small fishes and benthic soft-bodied invertebrates to hard-shelled molluscs and crustaceans that lived in the Bolca palaeobiotope (Carnevale & Pietsch 2009; Bannikov & Carnevale 2010; Carnevale *et al*. 2014; Dominici 2014; Giusberti *et al*. 2014; Pasini *et al*. 2019).

### Origin of the pelagic lifestyle

The inclusion of †*Dasyomyliobatis* in a phylogenetic framework suggests that the steps allowing the shift toward pelagic environment were: (1) modification of anteroposterior disc symmetry, with more fin rays distributed posteriorly on fins; (2) change in disc shape, with increase in AR, head protruding disc, and appearance of cephalic lobes; (3) achievement of crustal calcification and cross-bracing on pectoral fin-radials; (4) further increase in AR (>3.0) with consequent final configuration of the wing-like shape (Fig. 4).

**Fig. 6:**
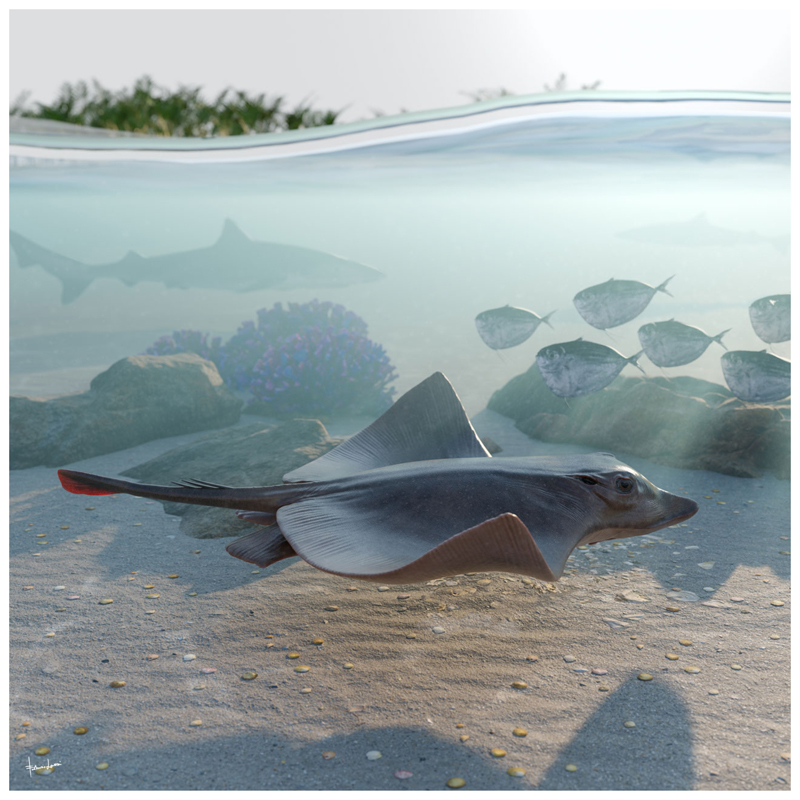
Life reconstruction of †*Dasyomyliobatis thomyorkei* gen. et sp. nov. swimming in the marine tropical shallow waters of the western Tethys about 50 million years ago. Artwork by Fabrizio Lavezzi.

The presence of catenated calcification with neither interradial cross-braces nor *compagibus laminam* suggest that †*Dasyomyliobatis* had a flexible pectoral disc and probably a more benthic lifestyle with undulatory swimming near the seafloor (Fig. 6). However, the positive FRD, moderate lateral expansion of fins, and head protruding from disc are consistent with oscillatory swimming. Interestingly, its AR (2.69) is intermediate between that of rajobenthic (<2.0) and aquilopelagic ecomorphotypes (>3.0), similar to that of *Gymnura*, a peculiar sting-ray closely related to hexatrygonids, urolophids and plesiobatids (Aschliman *et al*. 2012a; Last *et al*. 2016) with wing-like pectoral fins stiffened by radials with crustal calcification and cross-braces but without cephalic lobes or head protruding from disc. These features allow *Gymnura* to shift between undulatory and oscillatory swimming, giving this taxon unique hybrid swimming capabilities (Rosenberger 2001; Martinez *et al*. 2016). With a combination of rajobenthic and aquilopelagic traits and intermediate AR, †*Dasyomyliobatis* might have had similar swimming capabilities, allowing this stingray to exploit a wide range of habitats, from the heterogeneous shallow marine context characterized by lagoons, sand bottoms, seagrass beds, coral reefs to the open sea (Landini & Sorbini 1996; Carnevale *et al*. 2014; Marramà *et al*. 2016a).

Our time-calibrated phylogeny (Fig. 4; Fig. S5) confirms the hypothesis that the most recent common ancestor of extant aquilopelagic stingrays was a Late Cretaceous oscillator-swimmer and that the gradual achievement of their traits started at least in the early Late Cretaceous in those stingray taxa that started at the same time transition toward durophagy.

### Origin and role of cephalic lobes

It has been suggested that by adopting key functions associated with prey manipulation, the cephalic lobes may have released the pectoral fins from constraints associated with feeding, facilitating the evolution of oscillatory swimming in myliobatids and subsequent invasion of the pelagic environment (Mulvany & Motta 2013; Swenson *et al*. 2018). In this perspective, the presence of cephalic lobes in a fossil stingray without crustal calcification, cross-braces or *compagibus laminam* might suggest two alternative hypotheses:

1. †*Dasyomyliobatis* used its pectoral disc both for swimming and manipulating prey (still no functional separation of roles). Being unstiffened, its pectoral disc might have been still soft and flexible enough to allow prey manipulation, whereas its cephalic lobes were just useful to detect/locate prey.
2. †*Dasyomyliobatis* used its pectoral disc just for swimming, whereas cephalic lobes were already capable of manipulating prey (functional separation of roles). If so, stiffening of the pectoral disc cannot be considered to be a disadvantage in feeding efficiency that caused functional separation of these roles as suggested by Hall *et al*. (2018) since †*Dasyomyliobatis* has neither crustal calcification nor cross-bracing or *compagibus laminam*. Rather, it might have been the protrusion of the head from the pectoral disc and/or its shape change with increase in AR that caused a disadvantage in feeding efficiency, making evolution of cephalic lobes necessary to replace the role of pectoral disc for prey manipulation.

In any case, the presence of cephalic lobes in †*Dasyomyliobatis* suggests that they evolved before the appearance of crustal calcification and interradial joints, therefore before the shift to full oscillatory swimming *(contra* Mulvany & Motta 2013).

### Ontogeny and phylogeny

The study of major dental transformations in fossil and living stingrays provides another example of integration of embryological with palaeontological data, as some evolutionary steps related to the achievement of extreme durophagy can be also detected through ontogenetic development of myliobatid stingrays. Underwood *et al*. (2015) used embryos/neonate individuals of *Myliobatis* to describe the ontogenetic development of the myliobatid dentition. Symphyseal teeth of adult *Myliobatis* have up to 30 root lobes, a tight interlocking mechanism, and are seven times mesiodistally wider than labiolingually deep (Hovestadt & Hovestadt-Euler 2013). However, early stages of development show that symphyseal teeth are subrhombic to subhexagonal, lack interlocking mechanism, and the roots bear 1–2 poorly developed grooves separating 2–3 lobes. Neonate specimens of *Myliobatis* have symphyseal teeth with subhexagonal crown, incomplete interlocking mechanism, moderate mesiodistal enlargement, and up to four grooves separating five wide-block irregularly spaced root lobes (Underwood *et al*. 2015), suggesting that the mesiodistal elongation is coupled with the increase in number of root lobes through ontogeny. Intriguingly, dental features of neonate *Myliobatis* closely resemble those of the adult †*Dasyomyliobatis*. The timing of ontogenetic transformations of these features related to the achievement of complete durophagy in *Myliobatis* is congruent with the phylogenetic transformation of the same features based on our tree topology, corroborating the hypothesis of a gradual achievement of dental traits from a benthic soft-prey feeder ancestor.

### Implications for locomotion and lifestyle

The remarkable relationship between PC1 scores and AR suggests that a considerable component of pectoral-shape variation in stingrays is associated with AR, and therefore, related to function. This relationship corroborates the evidence that pectoral-shape variation is a critical component for the evolution of swimming mode in stingrays, showing that AR is related to swimming mode (oscillatory/undulatory) and environment (benthic/pelagic) (Fontanella *et al*. 2013; Martinez *et al*. 2016). Martinez *et al*. (2016) provided evidence showing that the large AR range of stingrays reflects their total range of pectoral forms, which is the largest among batoid orders (also including Rajiformes, Torpediniformes, Rhinopristiformes) and contributes to their highest morphological disparity. Intermediate AR values (2.0 < AR < 3.0) do not usually characterize living stingrays (except *Gymnura)* but rather skates (Rajiformes). However, our study shows that this AR range was also occupied by extinct stingrays, including †*Dasyomyliobatis* and †*Promyliobatis*, allowing us to make some inferences related to the evolutionary origin of the aquilopelagic stingray ecomorph. In this perspective, †*Dasyomyliobatis* supports the hypothesis of Martinez *et al*. (2016) that the ancestor of all aquilopelagic stingrays had an AR that was intermediate compared to extant stingrays (Martinez *et al*. 2016), and that the pelagic radiation of stingrays might have started in one of the Cretaceous pulses of diversification across elasmobranchs (Guinot *et al*. 2012) but well before the radiation of benthic Cretaceous–Palaeogene survivors into vacated ecospace (Bertozzi *et al*. 2016).

## Conclusions

**Fig. 7:**
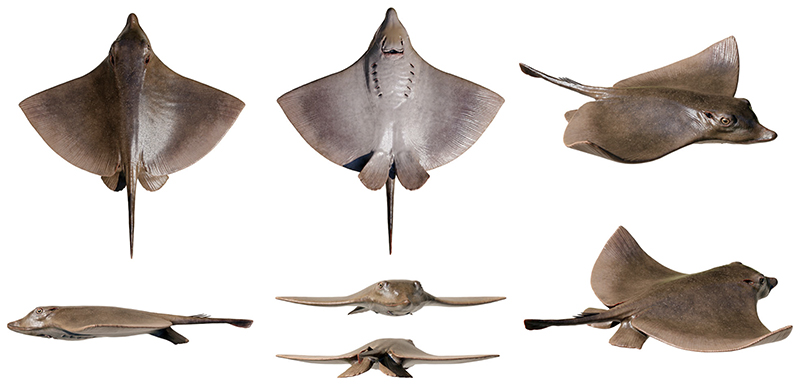
Hypothetical reconstruction of †*Dasyomyliobatis thomyorkei* gen. et sp. nov. in different views. Artwork by Fabrizio Lavezzi.

The unique body plan of †*Dasyomyliobatis* (Figs 6, 7) suggests that:

1. †*Dasyomyliobatis* is a representative of a new stingray family with unique hybrid dentition and pectoral-fin morphology that allowed the shift from undulatory to oscillatory swimming, and to exploit a variety of prey (from soft-bodied to hard-shelled organisms).
2. The evolutionary origin of durophagy and pelagic lifestyle was achieved through gradual transformation of morphological traits at least since the early Late Cretaceous.
3. Cephalic lobes were already present in a stingray without crustal calcification, cross-bracing, or *compagibus laminam* corroborating the hypothesis that they originated before the shift toward exclusive oscillatory locomotion and occupation of pelagic environments.
4. The phylogenetic analyses highlight that evolutionary modifications of the dentition related to a shift toward extreme durophagy seem to covariate with the evolution of traits of pectoral skeleton related to a shift toward a pelagic lifestyle, possibly reflecting high level of integration. One can speculate that although invasion of new habitats allowed initially stingrays to exploit wider ranges of food items, competition with other benthic batoids like skates, known to occupy today the same niche as benthic stingrays in deeper and cooler waters (Ebert & Compagno 2007) but also warm and shallow waters during the Late Cretaceous (Cappetta 1980), pushed pelagic/benthopelagic stingrays to specialize for a less exploited food resource, like hard-shelled invertebrates, although this hypothesis needs to be tested.

## Systematic Palaeontology

Class CHONDRICHTHYES Huxley, 1880

Superorder BATOIDEA Compagno, 1973

Order MYLIOBATIFORMES Compagno, 1973

Family †DASYOMYLIOBATIDAE nov.

*LSID*. https://zoobank.org/NomenclaturalActs/9AA3BB0E-9D18-4108-B0F0-D1069BB8ACA5

### Genus †DASYOMYLIOBATIS nov

#### LSID

https://zoobank.org/NomenclaturalActs/5106152A-3688-41EB-BD94-24DF635CB4DD

#### Type species

†*Dasyomyliobatis thomyorkei* sp. nov.

#### Derivation of names

Genus and family names refer to the peculiar mosaic of dasyatoid and myliobatoid traits.

#### Diagnosis

As for type and only species.

### †*Dasyomyliobatis thomyorkei* sp. nov Figures 2, 8–19

#### LSID

https://zoobank.org/NomenclaturalActs/A9A82AC2-FFD1-420F-A74A-9769D46BD96C

#### Derivation of name

After the British composer and musician Thom Yorke.

#### Holotype

MCSNV VR.21.107/8 well preserved, complete, and articulated skeleton in part and counterpart, 99.9 cm disc width.

#### Type locality & horizon

Pesciara site, Bolca Konservat-Lagerstätte, Italy; early Eocene, late Ypresian, middle Cuisian, SBZ-11, †*Alveolina dainelli* Zone, *c*. 50 Ma (Papazzoni *et al*. 2014).

#### Diagnosis

Stingray unique in having a peculiar combination of dasyatoid and myliobatoid traits. Like dasyatoids, †*Dasyomyliobatis* has a soft and flexible pectoral disc with convex anterior and posterior fin margins supported by catenated radials with no crossbracing; numerous (*c*. 40) labiolingually directed files of holaulacorhizous lateral teeth arranged in alternating rows; tail formed by free vertebrae not stiffened by a cartilaginous rod; caudal fin reduced to a ventral fold. Like myliobatoids, †*Dasyomyliobatis* shows a head protruding anterior to a wing-like pectoral disc; cephalic lobes contacting along their mesial edge forming a single, shovel-like structure; cephalic-lobe radials discontinuous with pectoral-fin radials; moderately enlarged hexagonal symphyseal/parasymphyseal polyaulacorhizous teeth in pavement-like arrangement, with bulbous/irregular interlocking mechanism, 3–4 wide-block and irregularly spaced root lobes. In addition, †*Dasyomyliobatis* has a pectoral-fin AR between 2.0 and 3.0, and positive FRD.

#### Differential diagnosis

†*Dasyomyliobatis* differs from families Dasyatidae, Potamotrygonidae, Urolophidae, Urotrygonidae, Plesiobatidae, Hexatrygonidae, Gymnuridae, and genera †*Heliobatis*, †*Asterotrygon* and †*Lessiniabatis*, in having head protruding a wing-like pectoral disc, cephalic lobes, and rows of moderately enlarged polyaulacorhizous symphyseal and parasymphyseal teeth (vs head resting inside a rounded to rhombic pectoral disc, cephalic lobes absent, and holaulacorhizous symphyseal and parasymphyseal teeth). †*Dasyomyliobatis* also differs from families Myliobatidae, Aetobatidae, Rhinopteridae, Mobulidae, and genera †*Promyliobatis* and †*Weissobatis*, in the presence of a soft and flexible pectoral disc supported by radials having catenated calcification and no cross braces, a tail with caudal fin reduced to single ventral fold, and in having small holaulacorhizous lateral teeth arranged in multiple alternating rows (vs pectoral disc stiffened by radials having crustal calcification and cross braces, no caudal fin, and few rows of enlarged polyaulacorhizous teeth in pavement-like arrangement).

### Description

**Fig. 8:**
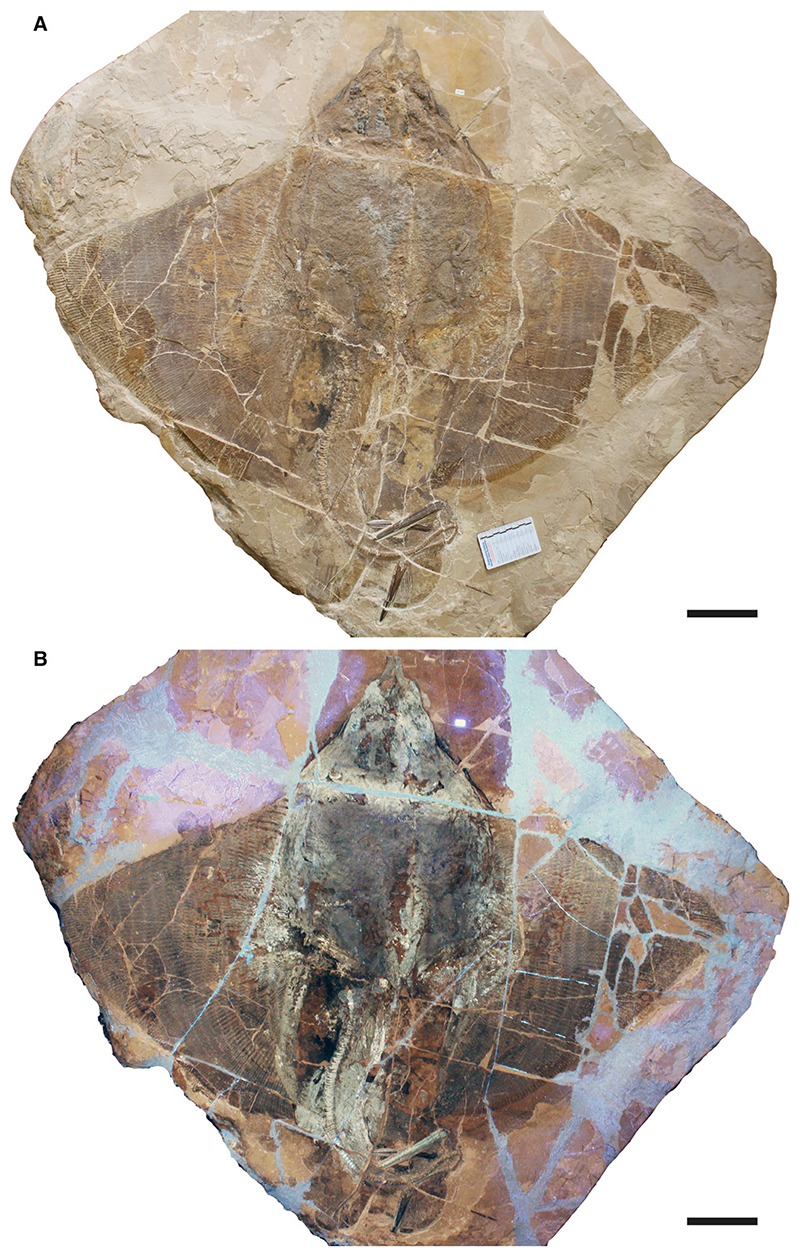
†*Dasyomyliobatis thomyorkei* gen. et sp. nov. from the Eocene of Monte Bolca (Italy). A, MCSNV VR.21.107, holotype, dorsoventral view natural normal light. B, the specimen under UV light. Both scale bars represent 100 mm.

**Fig. 9:**
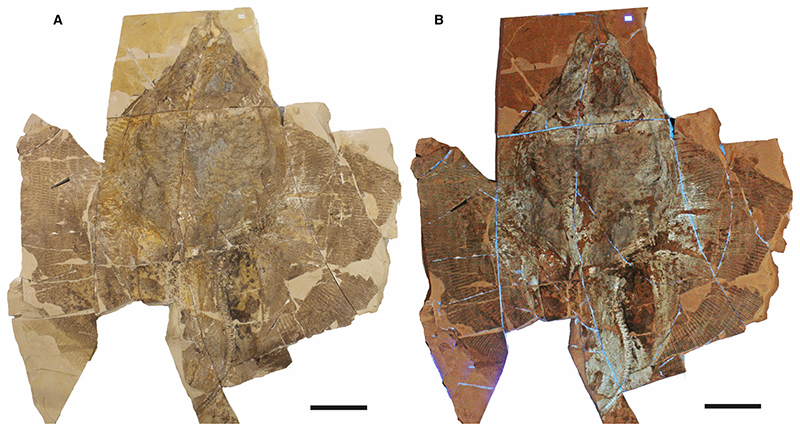
†*Dasyomyliobatis thomyorkei* gen. et sp. nov. from the Eocene Bolca Konservat-Lagerstätte (Italy). A, MCSNV VR.21.108 (counterpart), holotype, dorsoventral view under natural light. B, the specimen under UV light. Both scale bars represent 100 mm.

†*Dasyomyliobatis* is represented by a single, nearly complete and articulated skeleton preserved in two limestone slabs as part and counterpart (Figs 8, 9). Its good preservation allowed the recognition and description of several skeletal and dental characters, which are useful to distinguish and separate the taxon from any other known living and fossil stingray. The specimen examined represents an adult female characterized by disc width of 99.9 cm and a total length (from the anteriormost tip of cephalic lobes to the tip of tail) of 106.1 cm. Counts and measurements for †*D*. *thomyorkei* are listed in Table 1.

The head is large and protrudes anterior to pectoral disc. The disc of †*Dasyomyliobatis* is pseudorhombic or roughly wing-like (although not so expanded like in living aquilopelagic stingrays), with straight or slightly convex anterior edge and convex posterior margin. The pectoral disc reaches its maximum width in the anterior third of disc length. The disc width is about two times the disc length, and slightly less than the total length. The pectoral-fin aspect ratio (AR) calculated is 2.69. Contrary to most stingrays, the tail is short, about 38% of the disc width and 36% of total body length. It is unclear if †*Dasyomyliobatis thomyorkei* had dorsal fins. The skeleton is highly calcified and most of the skeletal elements show the typical prismatic calcification of elasmobranchs (Dean & Summers 2006).

#### Neurocranium

**Fig. 10:**
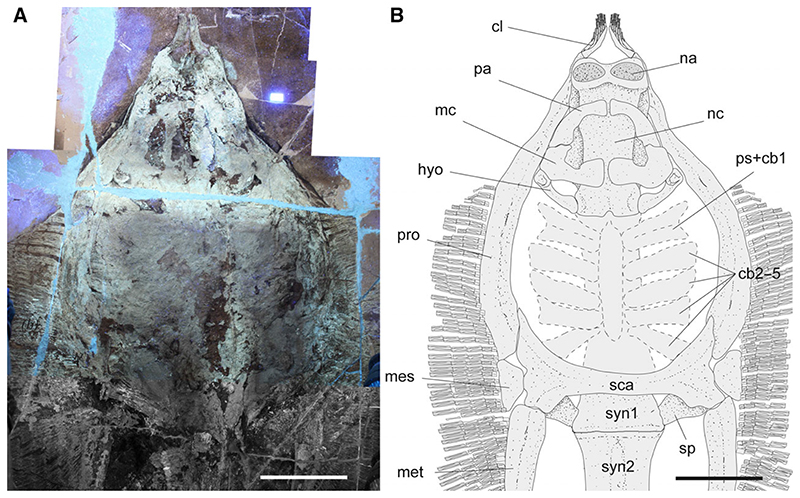
†*Dasyomyliobatis thomyorkei* gen. et sp. nov. from the Eocene Bolca Konservat-Lagerstätte (Italy). A, cranial region and pectoral girdle of MCSNV VR.21.107 under UV light; B, interpretative reconstruction (teeth are omitted). *Abbreviations*: cb, ceratobranchials; cl, cephalic lobes; hyo, hyomandibula; mc, Meckel’s cartilage; mes, mesopterygium; met, metapterygium; na, nasal capsules; nc, neurocranium; pa, palatoquadrate; pro, propterygium; ps, pseudohyoid; sca, scapulocoracoid; sp, scapular process of scapulocoracoid; syn1, cervicothoracic synarcual; syn2, thoracolumbar synarcual. Both scale bars represent 100 mm.

The neurocranium is anteroposteriorly elongate, longer than wide (Fig. 10). The rostral cartilage is inconspicuous as in all adult stingrays (Compagno 1977; Miyake *et al*. 1992). The nasal capsules are ovoid in shape and their anterior margin is rounded and biconvex with an anterior median indentation. There is no anterior process of the neurocranium typical of *Rhinoptera* and *Mobula*. Preorbital, postorbital and supraorbital processes are difficult to detect due to jaws and the large dental batteries that overlap most of head region. The antorbital cartilages are difficult to detect but it is likely that they were thin, unbranched, subtriangular, tapering distally and articulating with the propterygia as in all stingrays. The orbital region appears as longer than wide. The robust otic capsules provide articulation for the proximal portion of the hyomandibulae.

#### Jaws

Upper and lower jaws extend laterally and occupy almost the entire space between the propterygia. The jaws are displaced in an open gap therefore showing the large upper and lower dental batteries that overlap most of head region. As can be detected by the large area occupied by the lower dental battery, the lower jaw profile is strongly linguolabially expanded, as is typical for *Pastinachus* and all aquilopelagic stingrays (Underwood *et al*. 2017). Jaw antimeres appear slender and separated at the symphysis.

#### Hyoid and gill arches

The hyomandibulae are stout and robust, slightly arched and with a concave inner margin, laterally compressed and narrow at about their mid-length (Fig. 10). The hyomandibulae project anterolaterally, reaching the mesial wall of the propterygia just posterior to the posteroventral corner of the lower jaw. It is difficult to detect how the hyomandibulae articulate with lower jaw but likely that they articulated through a strong, stout ligament (the hyomandibular–Meckelian ligament) at distal tip, as in most stingrays (Carvalho *et al*. 2004). Its distal portion is strong and stout, whereas their proximal portion at the articulation with the otic region of the neurocranium is enlarged and stouter than its mesial part. It is not possible to detect the angular cartilages typical of potamotrygonids or the secondary hyomandibular cartilages characteristic of *Urolophus* and pelagic stingrays (Lovejoy 1996; Carvalho *et al*. 2004; Claeson *et al*. 2010). The ventral gill arches of †*Dasyomyliobatis* are only partially preserved and their overall morphology is poorly defined. The outline of the central medial plate, which results from the fusion of the basibranchial copula and the basibranchial components (Miyake *et al*. 1992; Carvalho *et al*. 2004), is difficult to discern but the median anterior projection of the basibranchial medial plate is weakly visible. There are possibly five pairs of ceratobranchials articulating with the lateral margin of the medial plate. The last two ceratobranchials appear ankylosed to each other but not fused in their proximal portion, whereas the fifth ceratobranchial pair articulates with the anterior margin of the scapulocoracoid. Filamentous branchial rays associated with the ceratobranchials can be recognized, although their number on each ceratobranchial cannot be detected.

#### Synarcuals and vertebral column

**Fig. 11:**
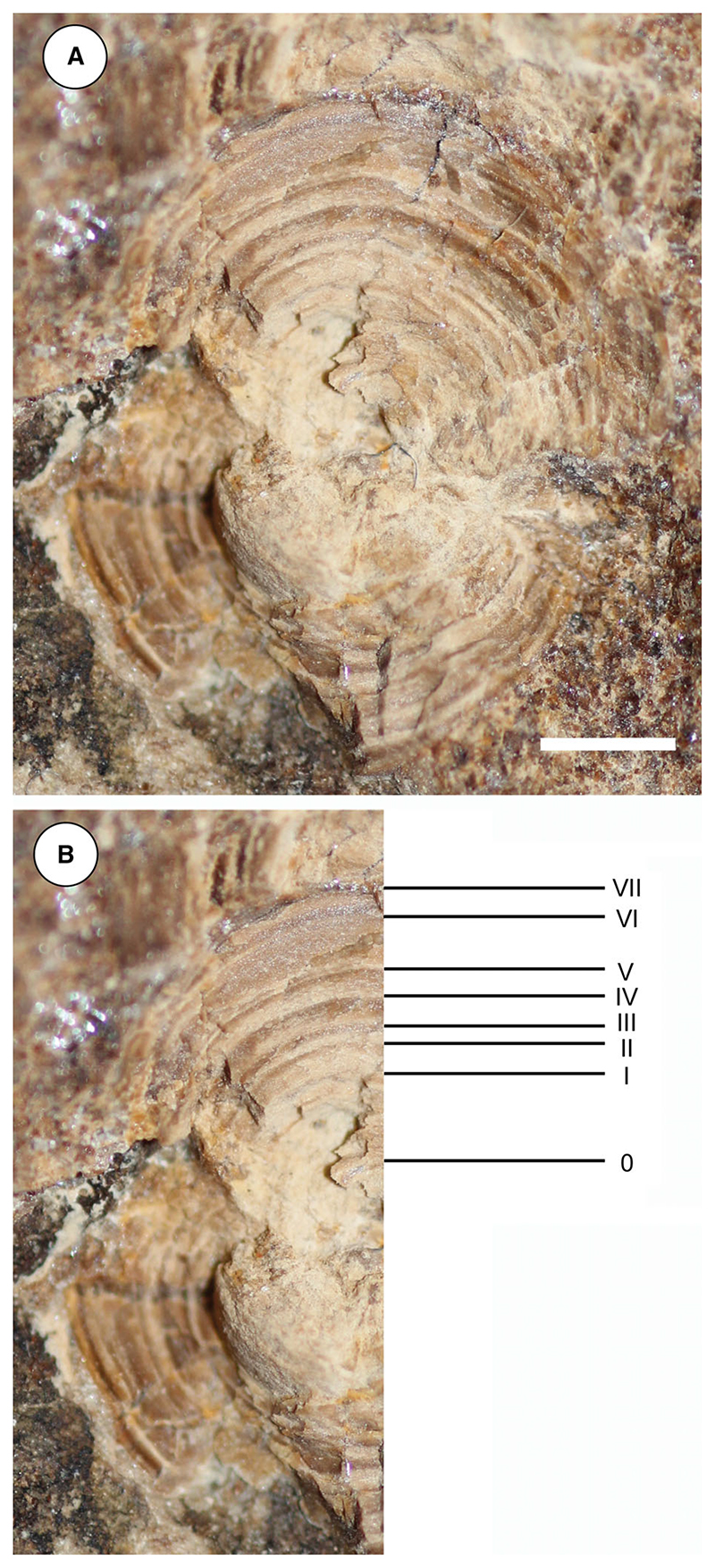
A, vertebral centrum of 7-year-old †*Dasyomyliobatis thomyorkei* gen. et sp. nov. (99.9 cm DW). B, the same centrum interpreted for age assignment; the external margin of the birth ring is indicated with 0; subsequent annual rings are marked I–VII. Scale bar represents 2 mm.

The general outline of the anterior (cervicothoracic) and posterior (thoracolumbar) synarcual cartilages can be easily recognized, although most of their calcified prismatic tesserae are taphonomically displaced (Figs 8–10). The cervicothoracic synarcual is strongly calcified and articulates with the occipital condyles of the chondrocranium. The odontoid process is not discernible. The lateral stays are not visible due to the presence of the scapulocoracoid overlapping them. The posterior (thoracolumbar) synarcual, which is uniquely present in stingrays among batoids, articulates anteriorly with the cervicothoracic synarcual but, contrary to this latter, it has a simpler structure, being triangular and tapering posteriorly. Although poorly preserved, the thoracolumbar synarcual is clearly shorter than the cervicothoracic synarcual, ending posterior to the scapulocoracoid. Individual vertebral centra can be recognized throughout the vertebral column length from the posterior tip of second synarcual to the tip of tail. The vertebral column of †*Dasyomyliobatis* consists of about 120 free vertebral centra (excluding those fused forming the synarcuals). The vertebral centra are small (diameter is up to 15 mm), anteroposteriorly short, subrectangular in lateral view, and subcircular in anterior or posterior view. An isolated and displaced vertebra shows at least seven growth rings, externally to the birth ring (Fig. 11). If annual couplets are assumed, the centrum would refer to a stingray individual with an estimated minimum age of seven years. The von Bertalanffy growth curves of the closest living relatives of †*Dasyomyliobatis* corroborate this hypothesis, as they indicate an age range of 5.3–12.2 years for the individual (standard errors range from ±0.2 to ±2.9 years; Table S1), based on disc width, whereas comparison of the age at maturity of most of living taxa with the age of MCSNV VR.21.107/8 (see Table S1) suggests that this latter represents a sexually mature individual. Neural arches are visible in the abdominal cavity posteriorly to the scapulocoracoid bar up to the level of the stings; neural spines are long, laterally compressed, and postero-obliquely oriented in relation to the centra. Haemal arches, being much smaller in comparison to the neural arches (Carvalho *et al*. 2004) are difficult to distinguish. The distal portion of the vertebral column posterior to the caudal stings is not stiffened by any cartilaginous rod, which is typically present in dasyatids, potamotrygonids and pelagic stingrays (Carvalho *et al*. 2004). Conversely, the distal portion of vertebral column is composed of free distinguishable vertebrae that become posteriorly smaller and smaller, up to the distal tip of tail. Ribs are absent, like in all myliobatiforms (McEachran *et al*. 1996).

#### Pectoral fins and girdle

**Fig. 12:**
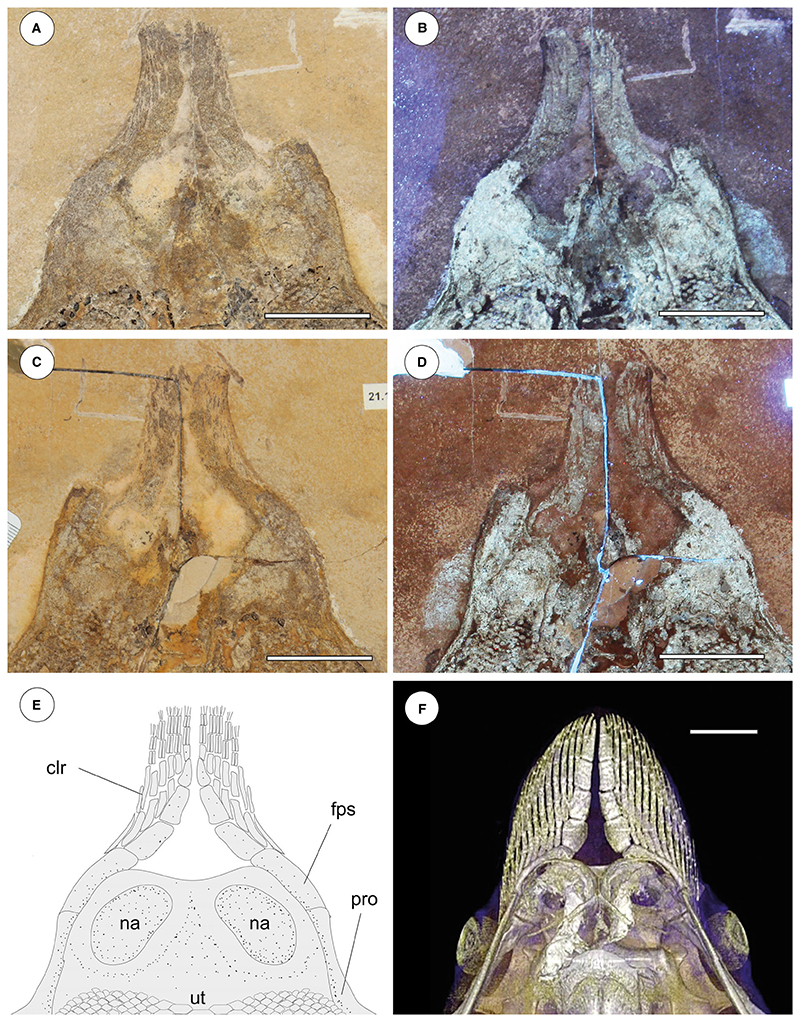
Cephalic lobes of †*Dasyomyliobatis thomyorkei* gen. et sp. nov. A, detail of MCSNV VR.21.107. B, the same region under UV light. C, detail of the counterpart MCSNV VR.21.108. D, the same region under UV light. E, interpretative reconstruction. F, 3D volume rendering technique (VRT) skull reconstruction from CT scans of extant spotted eagle ray (*Aetobatus narinari*) in ventral view for comparison; image courtesy of Prof. Frank Fish (West Chester University). *Abbreviations*: clr, cephalic lobe radials; fps, first propterygial segment; na, nasal capsules; pro, propterygium; ut, upper teeth. All scale bars represent 30 mm.

As in all stingrays, the scapulocoracoid of †*Dasyomyliobatis* is composed of a transverse ventral coracoid bar in the middle of the disc and lateral scapulae with dorsally projecting scapular processes (Fig. 10). The coracoid bar is a single, straight and robust transverse bar, located at the level of cervicothoracic synarcual, just anterior to its articulation with the thoracolumbar synarcual. The scapular processes are barely recognizable and appear as large subtriangular structures, obliquely oriented in relation to the synarcual cartilage. The scapular fossa (or foramen) cannot be recognized. Fusion of the suprascapulae with each posterodorsal region of the synarcual to create a well-defined bridge, which is unique to stingrays (Lovejoy 1996; Aschliman *et al*. 2012b), is difficult to detect. Laterally, the scapulocoracoid articulates with the internal skeleton of the pterygia. The propterygia are long, arched, tapering distally and extending beyond the anterior disc margin. The proximal portion of the propterygium is large, articulating with the anterior portion of the lateral margin of the scapula, and with the anteromedial margin of the mesopterygium. The propterygia are distally segmented with the first segment adjacent to the anterior margin of the nasal capsules and resembling the condition of aquilopelagic stingrays (Lovejoy 1996; Rosenberger 2001; Aschliman *et al*. 2012b). The cephalic lobes (Fig. 12A–E) are formed by 6-7 rostral radials each and that are separated from the other propterygial radials by a long gap, resembling the condition seen in *Aetomylaeus* and *Aetobatus*, rather than that of *Myliobatis* since this genus is characterized by rostral radials continuous with the other propterygial radials (White 2014). Cephalic lobes contact each other along their mesial margin, making their anterior margin continuous and forming a shovel-like structure in life, similar to the condition of *Aetobatus* (Fig. 12F), *Aetomylaeus* and *Myliobatis* (Mulvany & Motta 2013; White 2014; Swenson *et al*. 2018). Cephalic-lobe radials exhibit crustal calcification, have no cross-bracing, and bifurcate at least once at about mid-length.

**Fig. 13:**
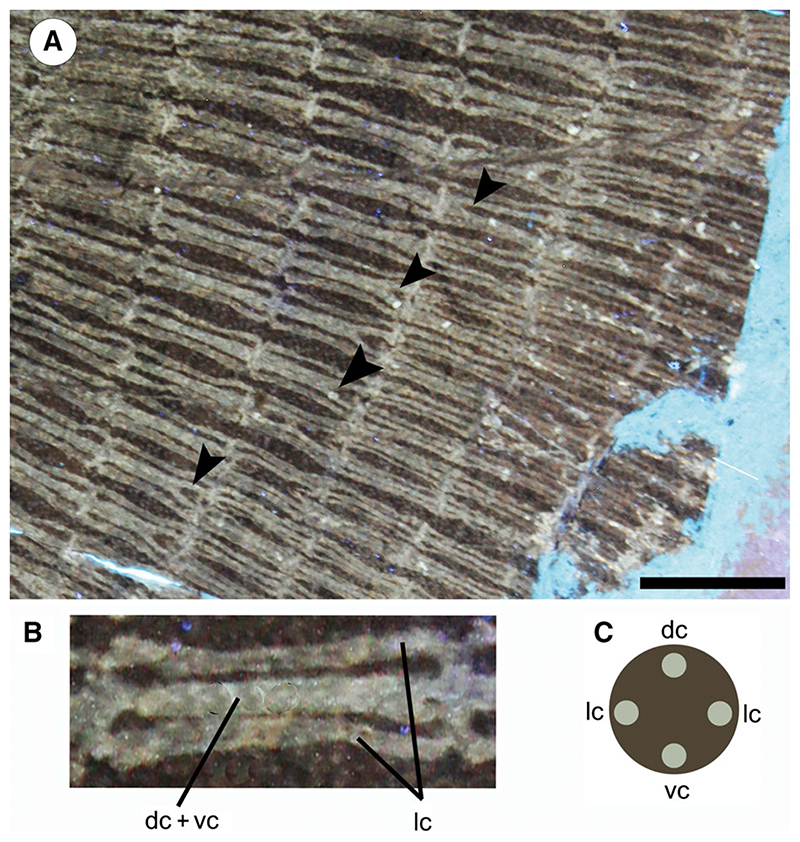
A, detail of pectoral radials of †*Dasyomyliobatis thomyorkei* gen. et sp. nov. (MCSNV VR.21.107) under UV light showing the four-chained catenated calcification; arrowheads indicate the bifurcation occurring at about 2/3 of radial length. B, close up of a single radial segment in which it is possible to recognize the two lateral chains and the overlapped dorsal and ventral chains. C, schematic reconstruction of the transversal section of the radial segment in B. *Abbreviations*: dc, dorsal chain; lc, lateral chains; vc, ventral chain. Scale bar represents 20 mm.

The mesopterygia are single and subtriangular; their external margins are more or less straight and not fused to the radials. The metapterygia are shorter than the propterygia, arched, and tapering posteriorly without reaching the anterior margin of the pelvic fins. There are 77–80 pectoral-fin radials of which 29–30 are propterygials (including those of cephalic lobes), 10 mesopterygials, and 38–40 metapterygials. The asymmetric distribution of radials on pterygia (metapterygials are more numerous than propterygials) makes positive the index of fin-ray distribution of †*Dasyomyliobatis* (FRD = 0.13), consistent with that of most dasyatids and all oscillatory swimmers. Contrary to *Gymnura* and aquilopelagic stingrays, †*Dasyomyliobatis* has no cross-braces and pectoral radials are calcified in a four-chain-like pattern (Fig. 13), forming the so-called ‘catenated’ calcification typical of batoids with undulatory swimming (all rajobenthic stingrays, except *Plesiobatis*; Schaefer & Summers 2005). Each radial is composed of at least 17–18 segments. Pectoral-fin radials bifurcate twice: the first bifurcation occurs distally at about 2/3 of the fin, whereas the last bifurcation occur near the fin margin. The *compagibus laminam* typical of aquilopelagic stingrays (except some *Myliobatis* species) is absent.

#### Pelvic girdle and fins

**Fig. 14:**
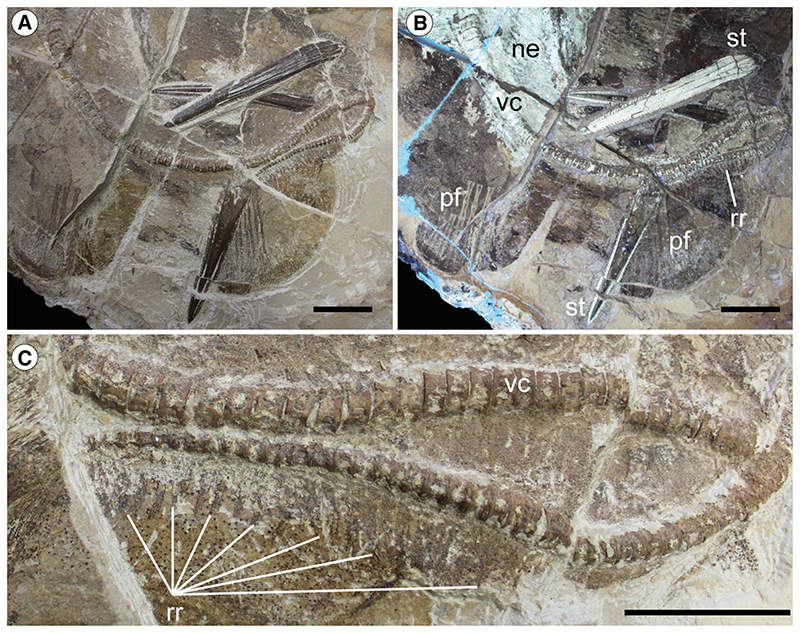
A, detail of pelvic-fin and caudal region of †*Dasyomyliobatis thomyorkei* gen. et sp. nov. (MCSNV VR.21.107) under natural light. B, the same region under UV light. C, close-up of the last portion of the tail showing the rudimentary radials supporting a ventral fold. *Abbreviations*: ne, neural arches; pf, pelvic fins; rr, rudimentary radials supporting the ventral fold; st, stings; vc, vertebral centra. Scale bars represent: 50 mm (A, B); 20 mm (C).

The puboischiadic bar and basipterygia are not preserved. However, the outline of the pelvic fins is easily recognizable although it is difficult to count the original number of pelvic-fin radials supporting them. The pelvic fins are single-lobed, protruding beyond the disc, and have convex posterior margins (Fig. 14A, B). Their length is about 18% of disc width. The pelvic fins are not overlapped by the posterior margin of pectoral fins, but this is probably due to taphonomic displacement. A few preserved radials show that they bifurcate distally once. Pelvic fins are devoid of claspers, therefore suggesting that the fossil represents a female individual.

#### Dorsal and caudal fins

The dorsal fin appears to be absent although we do not exclude the possibility that this is due to taphonomic loss. However, as most dasyatids (including the closely related *Pastinachus*) are devoid of dorsal fins, we might consider the condition of †*Dasyomyliobatis* to be a genuine feature. The caudal fin of †*Dasyomyliobatis* is reduced to a single ventral tail fold. In extant dasyatids, the tail region posterior to the caudal stings can exhibit small dorsal and ventral elements (rudimentary radials of Nishida 1990) supporting dorsal and ventral folds. Some dasyatid species exhibit only ventral elements that support a single ventral fold (Nishida 1990; Last *et al*. 2016). In our fossil, a single row of about 24 rudimentary radials can be recognized in the last portion of the vertebral column (Fig. 14C). Being rudimentary radials absent on dorsal aspect of the caudal vertebrae, it is likely that those present supported only a single ventral fold. The condition of the caudal fin of †*Dasyomyliobatis* therefore resembles that of *Pastinachus*, *Pteroplatytrygon* and *Neotrygon*, among stingrays (Nelson *et al*. 2016).

#### Dentition

**Fig. 15:**
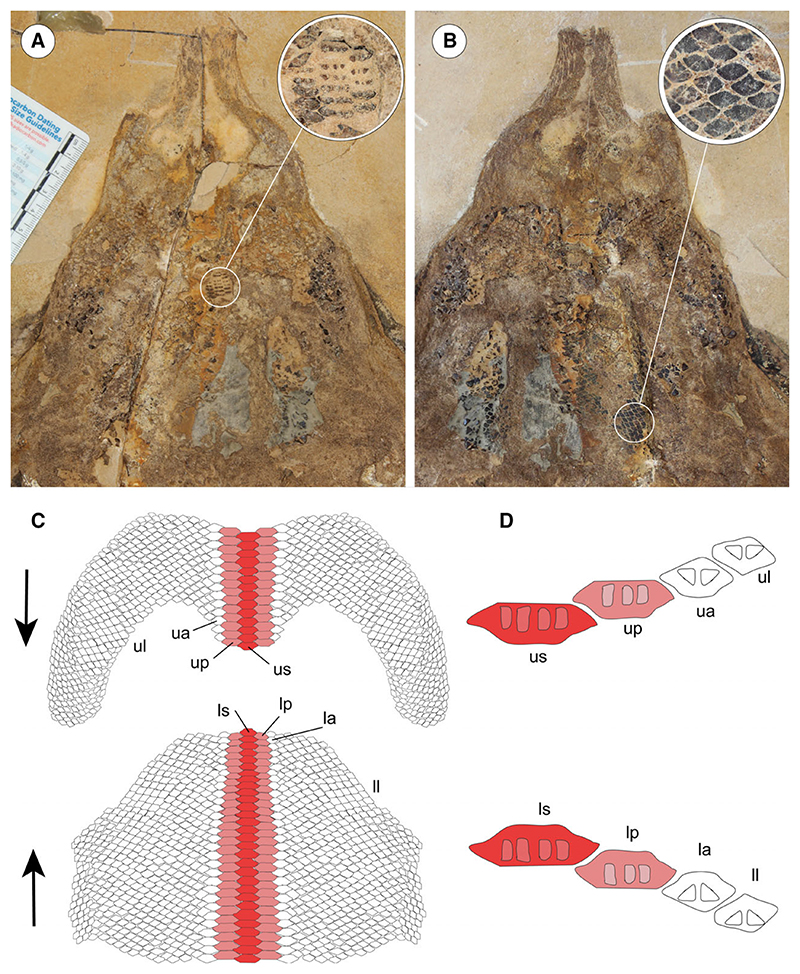
Dentition of †*Dasyomyliobatis thomyorkei* gen. et sp. nov. A, the oral region in MCSNV VR.21.108 with magnification of the area where the lower medial teeth are exposed. B, the oral region in MCSNV VR.21.107 with magnification of the area where the lower lateral teeth are exposed showing quincuncial disposition. C, interpretative reconstruction of the upper and lower dentition based on MCSNV VR.21.107 and VR.21.108. D, schematic outlines of crown and root lobes of teeth of †*D*. *thomyorkei* based on jaw position. Arrows indicate the labial direction. *Abbreviations*: la, lower anterolateral teeth; ll, lower lateral teeth; lp, lower parasymphyseal teeth; ls, lower symphyseal teeth; ua, upper anterolateral teeth; ul, upper lateral teeth; up, upper parasymphyseal teeth; us, upper symphyseal teeth.

The dentition of †*Dasyomyliobatis* is of crushing–grinding type (Fig. 15) as it has only moderate monognathic heterodonty with some teeth files of crushing type and others of grinding type, resembling the condition of *Pastinachus* and some Late Cretaceous to early Palaeogene taxa including †*Hypolophites*, †*Brachyrhizodus*, †*Hypolophodon* and †*Myliodasyatis* (Claeson *et al*. 2010; Cappetta 2012). Despite the evident taphonomic/lithostatic compression, we can recognize that the upper jaw dentition consists of two hemispheric parts showing small teeth narrowly imbricated and possibly separated by a deep and narrow symphyseal groove, like in *Pastinachus* (Adnet *et al*. 2019). The lower jaw dentition appears rather flat on the whole and seems to exhibit a more uniform and flatter ‘tooth plate’. Teeth on lower jaw are arranged in about 30 mesiodistally (laterally) directed rows and 40-45 labiolingually (anteroposteriorly) directed files, whereas teeth on upper jaw are arranged in about 15 mesiodistally directed rows and 40 labiolingually directed files. The dentition has moderate gradient monognathic heterodonty, as polyaulacorhizous medial (symphyseal and parasymphyseal) teeth have a different shape and are slightly more mesiodistally expanded than the adjacent holaulacorhizous lateral teeth. The presence of both polyaulacorhizous and holaulacorhizous teeth in the same individual makes †*Dasyomyliobatis* among the few fossil stingrays characterized by this pattern (Cappetta 2012) whereas this condition is unknown in living stingrays (excluding those cases in which *Pastinachus* and *Urogymnus* show some aberrant polyaulacorhizous teeth; Herman *et al*. 1998, 1999; Adnet *et al*. 2019).

**Fig. 16:**
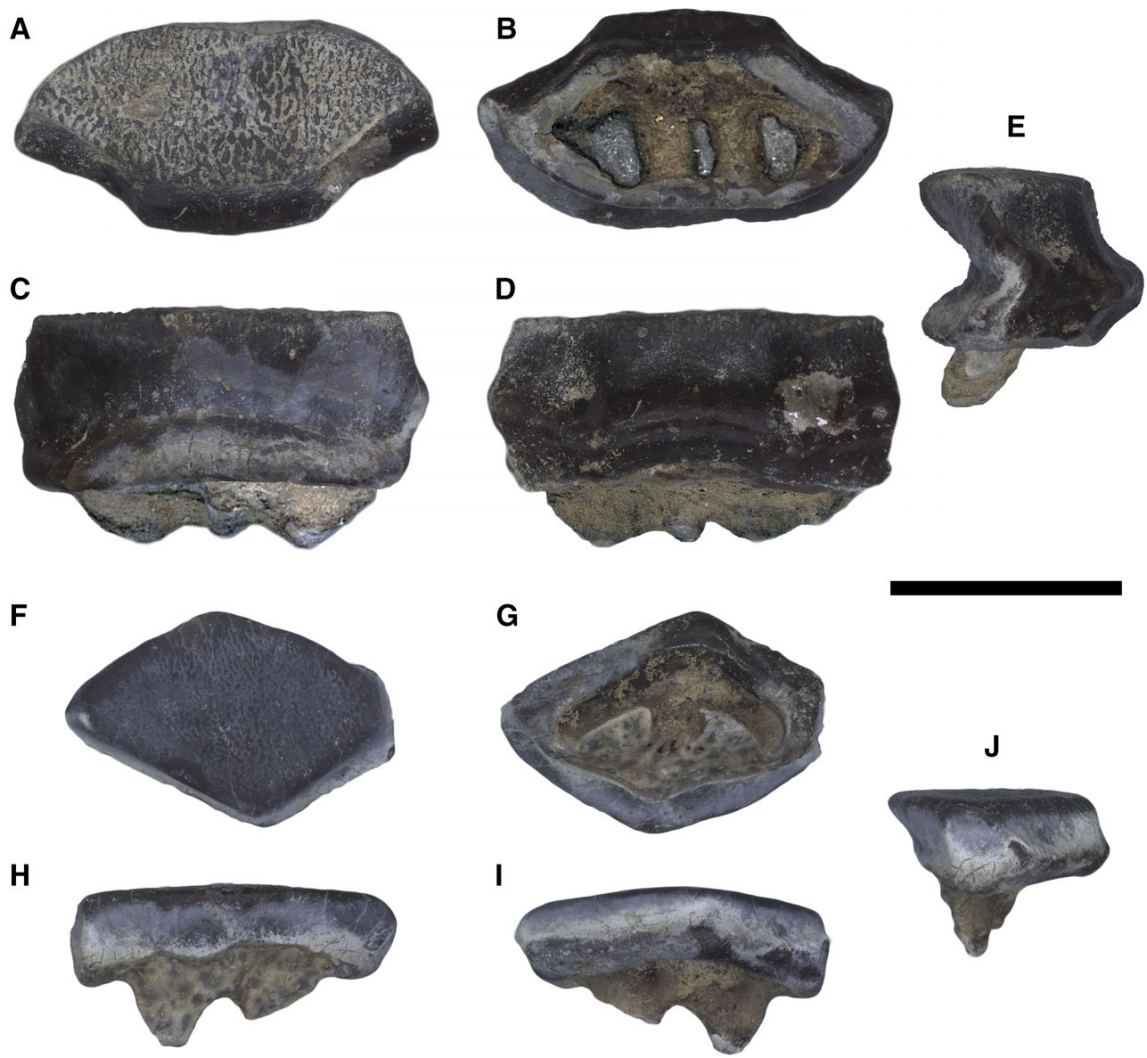
Isolated teeth of †*Dasyomyliobatis thomyorkei* gen. et sp. nov. A-E, polyaulacorhizous lower symphyseal tooth in: A, occlusal; B, basal; C, lingual; D, labial; E, profile view. F–J, holaulacorhizous lower lateral tooth in: F, occlusal; G, basal; H, lingual; I, labial; J, profile view. Scale bar represents 2 mm.

**Fig. 17:**
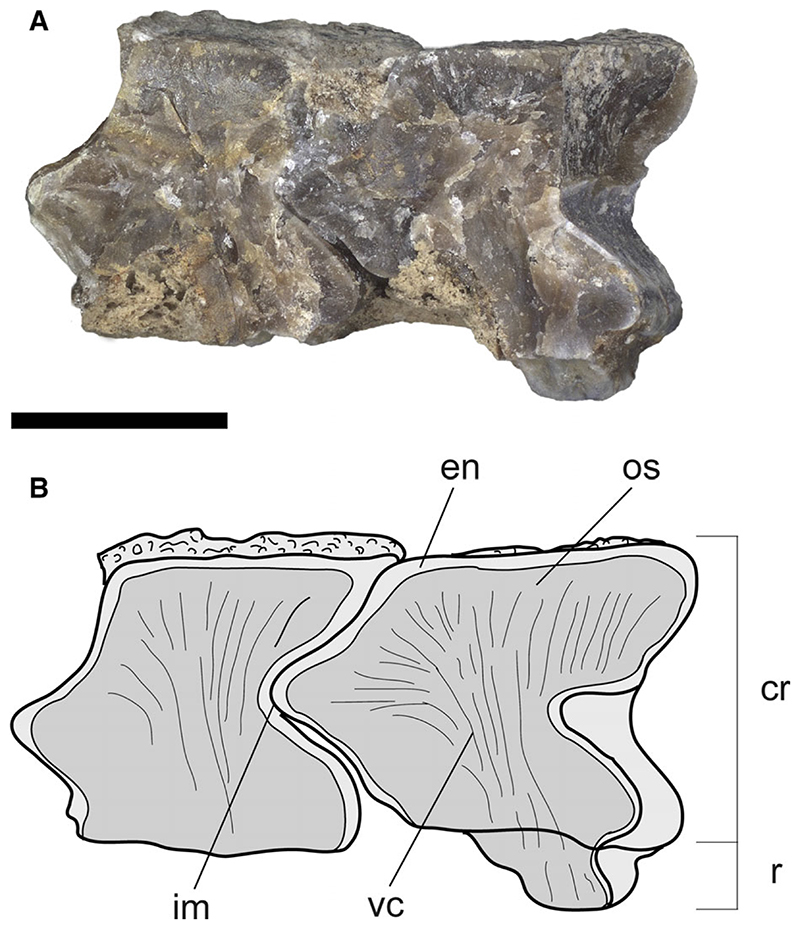
A, two symphyseal teeth of †*Dasyomyliobatis thomyorkei* gen. et sp. nov. in lateral view, still interlocked and in cross section. B, interpretative schematic drawing; note the absence of pulp cavity. Labial side on the left. *Abbreviations*: cr, crown; en, enameloid; im, interlocking mechanism (bulbous/irregular); r, root; os, osteodentine; vc, vascular canals. Scale bar represents 1 mm.

Individual teeth of †*Dasyomyliobatis* are small, with a crown width up to 4 mm (Fig. 16). The teeth have high and flat crown subparallel to the basal face of the root; the enameloid of the occlusal face bears small irregular vermiculi; the labial visor is very salient with an oblique lower face in profile view, and bears two transverse hollows at the base of the marginolabial faces; the transverse keel is sharp and parallel to the edge of the lingual visor; in profile view, the lingual face is very concave medially; the marginolingual faces are slightly convex then concave above the lingual bulge, which is usually very salient; the roots are less high than the crown in profile; tooth crown in all teeth overhangs completely the root in occlusal view. The tooth histotype resembles the ‘modified osteodont’ histotype reminiscent of that *Pastinachus* and all aquilopelagic stingrays (Herman *et al*. 1998, 2000) since the root lacks a pulp cavity and the whole crown is composed of osteodentine, which is crossed by irregularly shaped semi-parallel vascular canals that run mostly vertically (Fig. 17). Depending on jaw position, two main types of teeth can be recognized in both upper and lower jaws:

1. Median (symphyseal and parasymphyseal) teeth (Fig. 16A–E) exhibit pavement-like arrangement; their crown is moderately elongate (their width is two to three times greater than their labiolingual length) and with hexagonal or sub-hexagonal outline in occlusal view; the interlocking mechanism is bulbous/irregular (Fig. 17); roots of median teeth are of polyaulacorhizous type, with three or four lobes, which are wide-block in basal view and irregularly separated by 2–3 furrows. Several small foramina open in the root lobes.
2. Lateral teeth (Fig. 16F–J) are narrowly imbricated, exhibit quincuncial disposition and have no interlocking mechanism; they are slightly smaller than median teeth; in occlusal view, their crown is flat and sub-pentagonal to lozenge in occlusal view, with teeth becoming smaller toward posterior; roots are of holaulacorhizous type, with two lobes which are triangular in basal view and separated by a distinct furrow where opens a small central foramen.

#### Squamation and stings

**Fig. 18:**
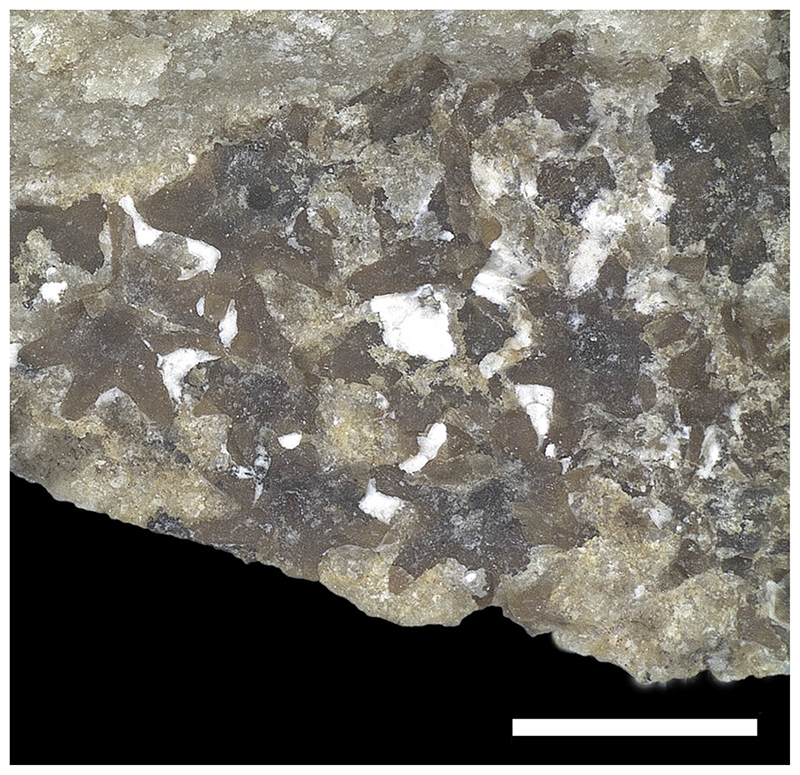
Star-shaped dermal denticles of †*Dasyomyliobatis thomyorkei* gen. et sp. nov. Scale bar represents 500 μm.

**Fig. 19:**
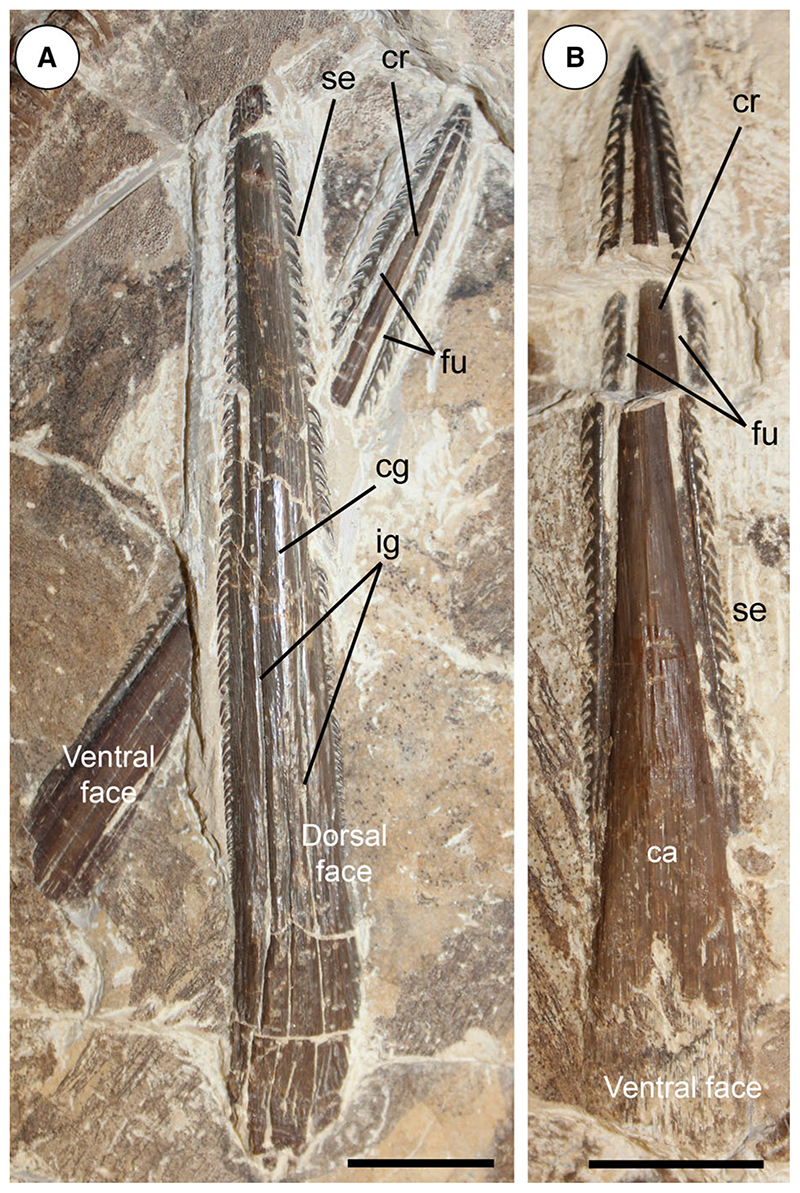
A, close-up of two overlapping stings. B, close-up of a third sting. *Abbreviations*: ca, cuneiform area; cg, central groove; cr, central ridge; fu, ventral furrows; ig, irregularly shaped grooves; se, serrations; st, stings. Scale bars represent 20 mm.

Sparse and scattered dermal denticles appear to cover the whole body of the specimen (Fig. 18). The denticles are very small (300–400 μm), star-shaped and equally spaced from each other. The local accumulation of some denticles in some body regions appears to be due to taphonomic bias. Thorns are absent. There are three serrated caudal stings with the largest one being 121.7 mm long (about 12% of disc width; Fig. 19). In rajobenthic stingrays the caudal stings are set on dorsal aspect of tail always posterior to pelvic fins and, depending on taxa, they can lie near the tail base, at about midlength, or even in its posterior half (Carvalho *et al*. 2004; Last *et al*. 2016). Conversely, in living aquilopelagic stingrays, the caudal stings are placed always very proximally on the tail, at the rear of a dorsal fin, which is located near the tail base, sometimes above the pelvic fins (Last *et al*. 2016). Due to taphonomic displacement, it is difficult to detect the exact position of each sting on tail of †*Dasyomyliobatis*. The stings are elongated, dorsoventrally flattened and taper toward the apex. One of the stings displays its dorsal side, whereas the other two show their ventral sides. The dorsal surface is gently convex and possesses a narrow but rather deeply incised central groove that originates near the spine base and runs for about two-thirds of the total spine length. Several shallower and irregularly shaped grooves run parallel and laterally to the main central groove.

The number and extent of these grooves is reminiscent of the later-stage caudal spines of adult extant stingray individuals (Halstead & Modglin 1950; Hovestadt & Hovestadt-Euler 2013), corroborating the hypothesis detected by the vertebral ring count and the von Bertalanffy growth curves. The whole dorsal surface of the sting reacts to UV light (Fig. 14B), due to the presence of a thin layer of enamel that covers the underlying dentine (forming the main body of the sting) as in living sting-rays (Halstead & Modglin 1950). The ventral sting surface (Fig. 19) shows a shallow, elongated, V-shaped depression in its proximal half (called cuneiform area) with a spongy appearance, and corresponding to the base of the sting arising from a thin cartilaginous matrix which is intimately associated with the neural spines of the caudal vertebrae (Halstead & Modglin 1950). The remaining distal part of the ventral sting surface is slightly convex. The distal two-thirds of the ventral sting surface features two parasagittal furrows that run along the sides of the spine and are separated from each other by a weak and broad central ridge. As in living stingrays, in life these two furrows accommodated the elongated venom-producing glandular epithelium (Halstead & Modglin 1950; Carvalho *et al*. 2004). Contrary to the dorsal surface, the ventral sting surface does not react to UV light, due to the absence of enamel that does not cover the underlying dentine (Fig. 14B). About 60 serrations are present along both sides of the longest and best-preserved sting. The serrations are small, hook shaped, directed toward the sting base, and transversely to the main axis of the caudal sting, forming with the latter an acute angle of about 30°. They decrease in size toward the apex and disappear before reaching it. There are no particular diagnostic features useful to discriminate the stings of †*Dasyomyliobatis* from those of other myliobatiforms, since the number and characters of the stings of rajobenthic and aquilopelagic taxa are uninformative from a taxonomic point of view (Hovestadt & Hovestadt-Euler 2013; Last *et al*. 2016).

#### Soft tissues

The specimen preserves traces of brown-coloured carbon films in the abdominal cavity (Figs 8, 9). In the area posterior to the scapulocoracoid and between the metapterygia, a large semi oval or crescent-shape brownish area is interpreted as representing the residue of the liver, occupying the same anatomical position of this organ in extant and extinct stingrays (Marramà *et al*. 2019b). The preservation of traces of the liver can be explained by its rich iron content, which provides a reddish or brownish colour to the limestone matrix. There is no abdominal gut content or traces of the eye pigmentation.

### Comparisons with extant and fossil stingrays

The morphological analysis of this new articulated fossil specimen has revealed the existence of a new stingray taxon in the Eocene Bolca Konservat-Lagerstaätte, whose body plan is unique among Myliobatiformes. The analysis has revealed the presence of a number of characters that clearly support the inclusion of †*Dasyomyliobatis* within the Myliobatiformes, including an inconspicuous rostral cartilage, the presence of venomous stings and thoracolumbar synarcual (Compagno 1973; Carvalho *et al*. 2004; McEachran & Aschliman 2004). However, its unique mosaic of plesiomorphic traits typical of benthic soft-feeder stingrays (the rajobenthic ecomorph) and derived characters typical of the durophagous pelagic stingrays (the aquilopelagic ecomorph), has never been found in any fossil or living stingray (Table 2), therefore supporting its inclusion in a new stingray family, the †Dasyomyliobatidae. This peculiar combination of characters argues against the placement of †*Dasyomyliobatis* within any living stingray families. In particular, the presence of a soft and flexible pectoral disc supported by poorly mineralized radials in catenated chain-like pattern, the multiple rows of holaulacorhizous small lateral teeth arranged in separate alternating rows with bilobed roots, a short tail with caudal fin reduced to single ventral fold excludes its assignment to the Myliobatidae, Aetobatidae, Rhinopteridae, Mobulidae, or its close relationship with the myliobatid-like †*Promyliobatis* and †*Weissobatis* (Hovestadt & Hovestadt-Euler 1999; Carvalho *et al*. 2004; Marramà *et al*. 2019c). At the same time, its head protruding a wing-like pectoral disc, the presence of cephalic lobes and polyaulacorhizous teeth, rules out its placement within Dasyatidae, Potamotrygonidae, Urolophidae, Urotrygonidae, Plesiobatidae, Hexatrygonidae, Gymnuridae, or its close relationships with other dasyatoid fossil stingrays such as †*Heliobatis*, †*Asterotrygon* and †*Lessiniabatis* (Carvalho *et al*. 2004; Marramà *et al*. 2019a).

The peculiar combination of dental characters of †*Dasyomyliobatis*, particularly the coexisting presence of both holaulacorhizous and polyaulacorhizous teeth, although unique among living stingray families, was recognized among some extinct fossil taxa erected based on dentition only, including †*Brachyrhizodus*, †*Heterobatis* and †*Meridiania*. †*Brachyrhizodus* is only known from isolated teeth from Campanian–Maastrichtian of North America, western Africa, and Madagascar (Cappetta 2012; Cicimurri *et al*. 2014). As in †*Dasyomyliobatis*, the tooth crown is thick, high, being asymmetrical and higher on its mesial than on its distal edge in lateral files. There is no lingual bulge. The crown of symphyseal teeth is generally hexagonal and broader than long. The root has 2–5 deep furrows separated by broad laminae irregularly spaced, whereas lateral teeth have bilobate roots (Cappetta 2012). However, contrary to †*Dasyomyliobatis*, teeth of †*Brachyrhizodus* have a smooth enameloid or ornamented by pits/hollow (vs vermiculi), larger size up to 25 mm (vs 4 mm), and a domed crown in anterior or posterior view (vs nearly flat). The Paleocene †*Heterobatis* from North Africa, described as having small teeth up to 3 mm which include both holaulacorhizous and polyaulacorhizous forms (Noubhani & Cappetta 1997; Cappetta 2012), can be distinguished from †*Dasyomyliobatis* by its strong monognathic heterodonty (vs moderate), and reticulate enameloid (vs vermicular). †*Meridiania* from Ypresian of North America has distinctly heterodont dentition with some hexagonal teeth, and others which are asymmetrical with an angular side and a more or less convex opposite side (Cappetta 2012). However, contrary to †*Dasyomyliobatis*, its teeth can be very large (up to 20 mm vs 4 mm), and the crown shows a kind of basal plateau, from which it rises into a high and thick transverse protuberance with a cutting crest which does not reach the lateral angles (Cappetta 2012) (a condition absent in †*Dasyomyliobatis*). Nevertheless, pending the discovery of more complete fossils including also cranial and postcranial skeletons, tooth-based stingray taxa characterized by the presence of both holaulacorhizous and polyaulacorhizous teeth like †*Brachyrhizodus*, †*Meridiana* and †*Heterobatis* might be tentatively included within the family †Dasyomyliobatidae.

Other taxa that were described based on teeth only that might resemble those of †*Dasyomyliobatis* are †*Aturobatis* and †*Myliodasyatis*. However, †*Aturobatis* teeth have a higher and less elongated crown size of less than 2 mm, and a clear dasyatoid pattern, with no polyaulacorhizous roots (Adnet 2006), whereas despite its crushing–grinding dentition, teeth of †*Myliodasyatis* have a domed crown in anterior or posterior view and alveolate ornamentation (Cappetta 2012). Finally, the polyaulacorhizous teeth of †*Amamriabatis* and †*Eomobula*, which might resemble the median teeth of †*Dasyomyliobatis*, have a more myliobatid or rhinopterid-like morphology, with larger convex occlusal face and wider crown, and consequent higher number of root lobes compared to †*Dasyomyliobatis*.

## Supplementary Material

Additional Supporting Information can be found online (https://doi.org/10.1111/pala.12669):

Report file, .tps and .nts files, and relative warp scores for geometric morphometrics

S1-S3, Fig.S1-S5, Tab.S1-S4

## Figures and Tables

**Table 1:** Main morphometrics and counts for †*Dasyomyliobatis thomyorkei* gen. et sp. nov.

Measurements	mm	% DW	% TL
Total body length (TL)	1061.0	106.1	100.0
Disc length	468.1	46.8	44.1
Disc width (DW)	999.9	100.0	94.2
Tail length	384.2	38.4	36.2
Scapulocoracoid width	238.7	23.9	22.5
Max sting length	121.7	12.2	11.5
Pelvics-tip of tail length	197.9	19.8	18.7
Neurocranial length	173.2	17.3	16.3
Neurocranial width (at nasal-capsule level)	67.8	6.8	6.4
Pre-sting length	743.9	74.4	70.1
Distance from tip of disc to max width disc	176.2	17.6	16.6
Prepelvic distance	676.8	67.7	63.8
Prescapular distance (head length)	439.6	44.0	41.4
Pelvic-fin length	179.7	18.0	16.9
Snout tip to the level of the greatest disc width	389.7	39.0	36.7
Pectoral-fin insertion to first sting	53.5	5.3	5.0
Meristics			
Propterygial radials	29–30 (including 6–7 rostral radials)		
Mesopterygial radials	10		
Metapterygial radials	38–40		
Total pectoral-fin radials	77–80		
Pelvic-fin radials	?		
Total free vertebrae	120		
Serrated tail stings	3		
Max sting serrations per side	60		
Other data			
Pectoral-fin aspect ratio (AR)	2.69		
Mean anteroposterior pectoralfin ray distribution (FRD)	0.13		

**Table 2:** Summary of selected body and dental features used to discriminate the stingray ecomorphotypes (data are taken from literature cited).

	†*Dasyomyliobatis* gen. nov.	*Gymnura*	Rajobenthic ecomorph	Aquilopelagic ecomorph
Body features				
Cephalic lobes	Present	Absent	Absent	Present
Pectoral-fin aspect ratio (AR)	2.69	2.0–3.0	<2.0	>3.0
Radial calcification	Catenated	Crustal	Catenated^1^	Crustal
Pectoral fin-ray distribution index (FRD)	Positive	Positive	Negative (positive in some dasyatids)	Positive
Posterior pectoral-fin border	Convex	Convex	Convex	Concave
*Compagibus laminam*	Absent	Absent	Absent	Present^2^
Cross-bracing	Absent	Present	Absent^3^	Present
Caudal fin	Reduced to tail fold	Absent	Present or reduced to tail fold	Absent
Head	Protruding the pectoral disc	Not protruding the pectoral disc	Not protruding the pectoral disc	Protruding the pectoral disc
Dental features^4^				
Symphyseal teeth (shape)	Hexagonal	Rounded/rhombic	Rounded/rhombic	Hexagonal
Lateral teeth (shape)	Rounded/rhombic	Rounded/rhombic	Rounded/rhombic	Hexagonal
Number of tooth files	20 or more	20 or more	20 or more	9–1
Holaulacorhizy	Present	Present	Present	Absent
Polyaulacorhizy	Present	Absent	Absent	Present
Symphyseal teeth (mesiodistal elongation)	Low/moderate	Absent	Absent	Strong
Tooth arrangement	Alternating diagonal row + pavement-like	Alternating diagonal row	Alternating diagonal row	Pavement-like
Dentition type	Crushing–grinding	Crushing	Crushing^5^	Grinding
Tooth histology	Modified osteodont	Orthodont	Orthodont or osteodont^6^	Modified osteodont
Tooth size	Small	Small	Small	Large
Monognathic heterodonty	Moderate	Low/gradual	Low/gradual	Strong

1 Except *Plesiobatis* (crustal).

2 Except *Myliobatis freminvillii* (absent).

3 Except *Urotrygon* (present).

4 Tooth reversal in *Mobula*.

5 Except *Pastinachus* (crushing–grinding).

6 Except *Pastinachus* (modified osteodont).

## Data Availability

This published work and the nomenclatural acts it contains, have been registered in ZooBank: https://zoobank.org/References/57E1240F-8625-47A2-896B-5CE670EEFDC0. Data for this study (including character list, detailed phylogenetic analyses; scripts and data matrices for phylogenetic analyses; report file, .tps and .nts files, and relative warp scores for geometric morphometrics) are available in MorphoBank: http://morphobank.org/permalink/?P4432.
