# Supplementary material for: The evolutionary origin of the durophagous pelagic stingray ecomorph
Source: Palaeontology. 2023 Jul 26;66(4):e12669. doi: 10.1111/pala.12669 (PMC7614867; doi:10.1111/pala.12669)
Supplement: Supplementary file 1 — Appendix S1. Character list. Appendix S2. Institutional abbreviations, comparative material, details of images used for geometric morphometrics and aspect ratio (AR) calculation. Appendix S3. Detailed results of the phylogenetic analyses. Figure S1. Single parsimonious tree retrieved based on 124 morphological characters and all 52 taxa. Figure S2. 50% majority rule tree based on 124 morphological characters and 40 holomorphic living and fossil taxa. Figure S3. Comparison of Bayesian and maximum likelihood tree topologies. Figure S4. Time‐calibrated tree based on 124 morphological characters and 52 taxa. Figure S5. Time‐calibrated tree based on 124 morphological characters and 40 holomorphic living and fossil taxa. Table S1. Age estimates for MCSNV VR.21.107/8 (†Dasyomyliobatis thomyorkei). Table S2. FAD and LAD data used for time‐calibrations of phylogenetic analyses. Table S3. Pectoral fin aspect ratios calculated for 35 taxa. Table S4. Results of PERMANOVA and ANOSIM shown as post‐hoc tests. [file PALA-66-0-s001.pdf]

SUPPORTING INFORMATION

The evolutionary origin of the durophagous pelagic stingray ecomorph

by GIUSEPPE MARRAMÀ[1]*, EDUARDO VILLALOBOS-SEGURA[2], ROBERTO ZORZIN[3], JÜRGEN KRIWET[2] *and* GIORGIO CARNEVALE[1]

[1]Dipartimento di Scienze della Terra, Università degli Studi di Torino, Via Valperga Caluso 35, 10125 Turin, Italy; giuseppe.marrama@unito.it; giorgio.carnevale@unito.it

[2]Department of Palaeontology, University of Vienna, Josef-Holaubek-Platz 2, 1090 Vienna, Austria; eduardo.villalobos.segura@univie.ac.at; juergen.kriwet@univie.ac.at

[3]Museo Civico di Storia Naturale di Verona, Lungadige Porta Vittoria 9, 37129 Verona, Italy; roberto.zorzin@comune.verona.it

*Corresponding author

APPENDIX S1

Character list

Most of characters and states are taken from literature (see *Abbreviations*). As previous matrices were often biased by discrepancy or misconstructions in coding of character states, the states were re-coded ex-novo by re-checking the descriptions and original character statements in literature. The new coding also benefited from personal observations for taxa listed in *Comparative Material*. ***Abbreviations***. BLA = Blanco (2019); CMG = De Carvalho *et al.* (2004); COR = Claeson *et al.* (2010); UKW = Underwood *et al.* (2017); ACM = Aschliman *et al.* (2012); MKDK = Marramà *et al.* (2018); MCNK = Marramà *et al.* (2019*a*); LLCL = Lim *et al.* (2015); VSM = Villalobos-Segura *et al.* (2022).

1. Tubules of subpleural components of hyomandibular lateral line canals (CMG1; COR1): (0) not branched at extremities; (1) extremities dichotomously branched.
2. Subpleural components of the hyomandibular lateral line canals (CMG2; COR3): (0) posterior branch extends caudally more or less parallel to longitudinal body axis; (1) posterior branch inflects toward midline to form a lateral hook; (2) posterior branch inflects to continue anteriorly almost parallel to anterior branch, forming a large indentation.
3. Suborbital components of infraorbital lateral line canals (CMG3; COR3): (0) projecting posteriorly lateral to mouth; (1) projecting posteriorly lateral to mouth and anteriorly lateral to nasal openings; (2) forming a complex web-like pattern on lateral aspects of the anteroventral disc region.
4. Scapular loops formed by scapular components of trunk lateral line canals (CMG4; COR4): (0) absent; (1) present.
5. Anterior process of neurocranium (CMG5; COR5): (0) absent; (1) present.
6. Preorbital process (CMG6; COR6): (0) present; (1) absent.

7. Preorbital canal for passage of superficial ophthalmic nerve (CMG7; COR7): (0) dorsally located; (1) anteriorly located.
8. Foramen for the optic (II) nerve (CMG8; COR8): (0) moderately sized; (1) very enlarged
9. Postorbital process of neurocranium (CMG9; COR9): (0) infraorbital lateral line canal separates postorbital process from small, anterior triangular outgrowth (supraorbital process) of the supraorbital crest; (1) postorbital process with small foramen for passage of infraorbital lateral line canal.
10. Extent of orbital region (CMG10; COR10): (0) orbital region of neurocranium long; (1) shortened orbital region with more anteriorly placed supraorbital and postorbital process.
11. Postorbital process (CMG11; COR11): (0) without ventrolateral projection; (1) continuing ventrolaterally to form a cylindrical projection.
12. Ventrolateral expansion of nasal capsules (CMG12; COR12): (0) nasal capsules laterally expanded; (1) nasal capsules ventrolaterally expanded.
13. Articulation between hyomandibula and Meckel's cartilage (CMG13; COR13): (0) hyomandibulae directly attached to lower jaws; (1) hyomandibulae articulating with lower jaws through strong, stout ligament (hyomandibular−Meckelian ligament) at distal tip.
14. Angular cartilages (CMG14; COR14): (0) absent; (1) present.
15. Secondary hyomandibular cartilages (CMG15; COR15): (0) absent; (1) present.
16. Symphysial fusion of upper and lower jaws (CMG16; COR16): (0) antimeres separate at symphysis; (1) both antimeres of jaws symphysially fused.
17. Mandibular width at symphysis (CMG17; COR17): (0) lower jaws slender at symphysis; (1) lower jaws symphysially thickened.
18. Lateral projections of lower jaws (wing-like processes) (CMG18; COR18): (0) absent; (1) present.
19. Basihyal cartilage (CMG20; COR19): (0) present; (1) absent.

20. Basihyal cartilage (if present) (CMG20; COR19): (0) laterally elongated, fused to first hypobranchialis; (1) basihyal a single element, but separate from first hypobranchials; (2) basihyal separate from first hypobranchials but fragmented into more than one component.
21. Fusion of ventral pseudohyoid and first ceratobranchial (CMG21; COR20): (0) absent; (1) present.
22. Arrangement of posterior ceratobranchials (CMG22; COR21): (0) separate from each other; (1) ankylosis between fourth and fifth ceratobranchials; (2) fourth and fifth ceratobranchials fused to each other.
23. Median projection of the basibranchial medial plate (CMG23; COR22): (0) absent; (1) present.
24. Articulation between fifth epi– and ceratobranchial elements to scapulocoracoid (CMG24; COR23): (0) close together; (1) widely separated.
25. Lateral stay of synarcual (CMG25; COR24): (0) originates ventral to spinal nerve foramina; (1) originates dorsal to spinal nerve foramina; (2) contacting synarcual both dorsally and ventrally to foramina.
26. Fossa on dorsal scapular region (CMG26; COR25): (0) absent; (1) present.
27. Contact between pro- and mesopterygium in the pectoral fin (CMG27; COR26): (0) present; (1) absent.
28. Mesopterygium (CMG28; COR27): (0) present; (1) absent/fused to scapulocoracoid.
29. Mesopterygium (when present) as (CMG28; COR27): (0) single element; (1) fragmented.
30. Lateral expansion of radials in pectoral region ('cross-bracing' of Schaefer & Summers 2005) (CMG29; COR28): (0) absent; (1) present. **Remarks**: Nishida (1990) and De Carvalho *et al.* (2004) initially detected this character only in *Gymnura*, *Myliobatis*, and *Aetobatus*. However, the most comprehensive and detailed analysis of Schaefer & Summers (2005) detected interradial cross-braces also in *Urotrygon*, *Rhinoptera*, and *Mobula*. Cross-braces are absent in †*Dasyiomyliobatis*.

31. External margin of mesopterygium (CMG30; COR29): (0) more or less straight, not fused to radials; (1) undulated, not fused to radials; (2) highly sinuous, appearing to be fused with articulating radial elements; (3) more or less straight, with fused radials.
32. Median prepelvic process (CMG31; COR30): (0) absent or weakly developed; (1) very elongated.
33. Pelvic girdle shape (CMG32; COR31): (0) not arched or only moderately so; (1) greatly arched
34. Dorsal fin (CMG33; COR32): (0) present; (1) absent.
35. Cartilaginous rod in tail (CMG34; COR33): (0) absent; (1) present.
36. Caudal fin (CMG35; COR34): (0) present; (1) absent.
37. Caudal fin (when present) (CMG35; COR34): (0) completely developed; (1) reduced to tail-folds.
38. Posteromedial extension of adductor mandibulae complex (CMG36; COR35): (0) absent; (1) present.
39. Spiracularis muscle (CMG37; COR36): (0) projecting ventrally to insert on either palatoquadrate, Meckel's cartilage, and or hyomandibula; (1) projecting ventrally and posteriorly beyond hyomandibulae and both sets of jaws to insert dorsal to coracomandibularis; (2) projecting ventrally and posteriorly beyond hyomandibulae and both sets of jaws to insert ventral to coracomandibularis.
40. Depressor mandibularis muscle (CMG38; COR37): (0) present; (1) absent.
41. Coracohyoideus muscle (CMG39; COR38): (0) not connected at midline; (1) connected at midline.
42. Urea retention (CMG40; COR39): (0) urea retained in blood; (1) urea excreted in urine.
43. Rectal gland (CMG41; COR40): (0) present; (1) reduced.
44. Spiracular tentacle (CMG42; COR41): (0) absent; (1) present.
45. Cephalic lobes (CMG43; COR42): (0) absent; (1) present.
46. Cephalic lobes (if present) (CMG43, COR42, modified): (0) single, fused at midline in a shovel-like structure with rounded apex (rostral lobe); (1) paired, fused at midline but having anterior indentation; (2) paired, widely separated as cephalic fins. **Remarks**: After splitting the character

following the logical bases underlying characters statements in phylogenetic analyses (Sereno 2008), we modified the original coding of previous publications (De Carvalho *et al.* 2004; Claeson *et al.* 2010). These publications erroneously coded *Aetobatus* as having anterior indentation, whereas it clearly shows the same condition of *Myliobatis* and *Aetomylaeus* in having cephalic lobes single, fused at midline in a shovel-like structure with rounded apex (rostral lobe) (state 0) used to unearth benthic prey (Mulvany & Motta 2013; Swenson *et al.* 2018). In the same way, previous publications coded *Rhinoptera* as having paired cephalic fins as mobulids (2), whereas its cephalic lobes are paired, fused at midline but having anterior indentation (state 1) (Mulvany & Motta 2013; Swenson *et al.* 2018). †*Dasyomyliobatis* shows the basalmost condition (0).

47. Nasal curtain (CMG44; COR43): (0) not reaching mouth region; (1) extending posteriorly as far as mouth opening.
48. Tooth arrangement (COR45): (0) arranged in separate alternating rows; (1) pavement−like arrangement.
49. Shape of symphyseal teeth (COR46, modified): (0) square to rounded; (1) hexagonal, six distinct sides; (2) rectangular with posteriorly deflected lateral margins. **Remarks**: since †*Dasyomyliobatis* and other fossil taxa present both squared/rounded and hexagonal teeth depending on position, we modified and split the original character statement of Claeson *et al.* (2010) in order to detect the first appearance of the derived conditions in both the symphyseal and lateral teeth (see ch. 50).
50. Shape of lateral teeth (COR46, modified): (0) all square to rounded; (1) square to hexagonal depending on tooth position; (2) all hexagonal.
51. Lateral teeth (COR47; UKW47): (0) present; (1) absent
52. Differentiation of symphyseal teeth from lateral teeth (monognathic heterodonty) (COR44,48; UKW48, modified): (0) median and lateral teeth are similar (low monognathic heterodonty); (1) median teeth relatively expanded mesio-distally (moderate monognathic heterodonty); (2) median teeth extremely expanded mesio-distally (strong monognathic heterodonty). **Remarks**: Most of

benthic stingrays and outgroups have symphyseal and lateral teeth which are similar in shape decreasing gradually in size toward posterior, therefore possessing low gradient monognathic heterodonty (0). The width of the median teeth is two to three times greater than their labiolingual length in †*Hypolophites*, †*Dasyomyliobatis*, †*Apocopodon,* †*Brachyrhizodus*, and *Mobula hypostoma*, making them having moderate monognathic heterodonty (1). Finally, *Aetomylaeus*, †*Burnhamia*, †*Igdabatis*, *Myliobatis* (including extinct species), †*Promyliobatis*, *Rhinoptera*, and †*Weissobatis* are characterized by strong monognathic heterodonty (2), as the width of the median teeth can be four up to 18 times greater than their labiolingual length with respect to lateral teeth (Cappetta 2012; Hovestadt & Hovestadt-Euler 2013).

53. Differentiation among lateral teeth (COR49; UKW49): (0) all lateral teeth unexpanded or only slightly expanded; (1) some lateral teeth very expanded.
54. Relative amount of curvature in expanded lower teeth (COR50; UKW50): (0) straight and uncurved; (1) moderately curved; (2) strongly curved.
55. Upper tooth curvature (COR51; UKW51): (0) uncurved; (1) curved.
56. Direction of tooth curvature (COR52; BLA53): (0) concave; (1) flat/horizontal; (2) convex; (3) sigmoidal.
57. Tooth interlocking mechanism (COR53; UKW45): (0) absent, (1) present.
58. Shape of interlocking tongue (COR55): (0) bulbous o irregular; (1) short shelf; (2) long shelf.
59. Crown height (COR56): (0) high, the crown height exceeds root depth on unworn teeth; (1) low crown.
60. Occlusal surface of teeth (COR57): (0) cusped; (1) flat; (2) depressed.
61. Crown shape in anterior or posterior view (COR58): (0) straight; (1) domed; (2) deep.
62. Root type in symphyseal teeth (COR60): (0) holaulacorhizous; (1) polyaulacorhizous. **Remarks**: since †*Dasyomyliobatis* and †*Brachyrhizodus* present both type of roots depending on tooth position, we modified and split the original character statement of Claeson *et al.* (2010) in order

to detect the first appearance of the derived conditions in both the symphyseal and lateral teeth (see also ch. 63).

63. Root type in lateral teeth (COR60): (0) holaulacorhizous; (1) polyaulacorhizous.

64. Number of root lobes in median teeth (COR61): (0) two; (1) three to five; (2) six or more. **Remarks**: since some fossil taxa like †*Dasyomyliobatis* and †*Brachyrhizodus* can present different states when median (symphyseal/parasymphyseal) or lateral teeth are considered, we modified the original character statement of Claeson *et al.* (2010) in order to detect the first appearance of the derived conditions in both medial and lateral teeth (see also ch. 65).

65. Number of root lobes in lateral teeth (COR61): (0) two; (1) usually more than two.

66. Root lobes in basal view in median teeth (COR62): (0) triangles; (1) wide blocks; (2) narrow blocks; (3) fine edges. **Remarks**: since †*Dasyomyliobatis* and †*Brachyrhizodus* show different states when symphyseal/parasymphyseal or lateral teeth are considered (also within the same taxon), we modified the original character statement of Claeson *et al.* (2010) in order to detect the first appearance of the derived conditions in both symphyseal and lateral teeth (see also ch. 67).

67. Root lobes in basal view in lateral teeth (COR62): (0) triangles; (1) wide blocks; (2) narrow blocks; (3) fine edges.

68. Distance between root lobes (COR63): (0) narrower than root laminae; (1) broad, groove wider than root laminae.

69. Inclination of roots (COR64): (0) roots perpendicular to the crown (no inclination); (1) offset and step−like; (2) long and strongly inclined.

70. Root groove position (COR65): (0) regularly spaced between laminae; (1) irregularly spaced between laminae.

71. Levator and depressor rostri muscles (ACM9): (0) absent; (1) present.

72. Serrated tail stings (ACM14): (0) absent; (1) present.

73. Placoid scales (ACM15): (0) present; (1) absent.

74. Placoid scales (ACM15): (0) uniformly present; (1) scattered.

75. Thorns (ACM16): (0) present; (1) absent.

76. Pulp cavities in tooth roots (ACM18): (0) present; (1) absent.

77. Pulp cavities in tooth roots (ACM18): (0) large; (1) broad and elongated; (2) small.

78. Tooth histotype (ACM19): (0) orthodont; (1) osteodont; (2) modified osteodont.

79. Infraorbital loop of suborbital and infraorbital canals (ACM21): (0) absent; (1) present.

80. Infraorbital loop of suborbital and infraorbital canals (ACM21): (0) forming a simple posterolaterally directed loop; (1) forming a complex reticular pattern or number of loops; (2) the loop is directed to the anterior.

81. Rostral cartilage (ACM26): (0) conspicuous/noticeable; (1) inconspicuous.

82. Postorbital process (ACM36): (0) narrow; (1) very broad and shelf-like.

83. Jugal arch (ACM39): (0) present; (1) absent.

84. Basihyal and first hypobranchial (ACM48): (0) both present and unsegmented; (1) basihyal is segmented; (2) basihyal is absent; (3) basihyal and first hypobranchial cartilages absent.

85. Suprascapulae (ACM50): (0) articulates with vertebral column; (1) fused medially to synarcual (= pectoral arch); (2) fused medially and laterally to synarcual.

86. Ball and socket articulation between scapular process and synarcual (ACM53): (0) absent; (1) present.

87. Second (thoracolumbar) synarcual (ACM54): (0) absent; (1) present.

88. Ribs (ACM55): (0) present; (1) absent.

89. Segmentation of propterygium (ACM63): (0) posterior to mouth; (1) proximal segment of propterygium of pectoral girdle is between mouth and antorbital cartilage; (2) the first segment is adjacent to the nasal capsule; (3) the first segment is adjacent to anterior margin of antorbital cartilage or anterior to margin of nasal capsule.

90. Pseudosiphon (ACM74): (0) present; (1) absent.

91. Dorsal marginal clasper cartilage (ACM75): (0) lacks medial flange; (1) possesses medial flange.

92. Dorsal terminal cartilage (ACM76): (0) smooth margin; (1) crenate margin.

93. Cartilage forming component claw (ACM77): (0) present; (1) absent.
94. Cartilage forming component claw (when present) (ACM77): (0) cartilage embedded in integument and is not visible externally; (1) cartilage lines the inner ventral margin of the clasper glans and often forms the component shield.
95. Ventral terminal cartilage (accessory terminal 1 cartilage in rajids) (ACM78): (0) simple; (1) free distally and forms component sentinel or is fused with ventral marginal cartilage and forms component projection; (2) folded ventrally along its long axis to form a convex flange.
96. Ventral terminal cartilage (accessory terminal 1 cartilage in rajids) (ACM79): (0) attached over length to axial cartilage; (1) free of axial cartilage.
97. Spiracularis (ACM85): (0) undivided; (1) splits into lateral and medial bundles, with the medial bundle inserting onto the posterior surface of Meckel's cartilage and the lateral bundle inserting onto the dorsal edge of the hyomandibula; (2) extends beyond the hyomandibula and Meckel's cartilage; (3) subdivided proximally and inserts separately onto the palatoquadrate and the hyomandibula.
98. Sexual heterodonty (UKW52): (0) absent; (1) present.
99. Medial symphyseal processes of the Meckel's cartilage (UKW63): (0) absent; (1) present.
100. Lateral processes of the palatoquadrate extending far anteriorly (UKW68): (0) absent; (1) present.
101. Anterior processes of the Meckel's cartilage (UKW69): (0) absent; (1) present.
102. Anterior processes of the Meckel's cartilage (UKW69): (0) not extending anterior past jaw joint; (1) extending anterior past jaw joint.
103. Lateral oral diastema alt (UKW71): (0) diastema width greater than occlusal width; (1) occlusal width greater than diastema width.
104. Upper jaw profile (UKW72): (0) oval in cross-section; (1) flat top, convex occlusal surface; (2) strongly flattened.
105. Upper jaw mineralization (UKW73): (0) all surfaces mineralized; (1) lingual face partly unmineralized.

106. Lower jaw profile (UKW74): (0) oval in cross-section; (1) strongly linguo-labially expanded.

107. Upper and lower jaw trabeculae (UKW75): (0) absent; (1) present.

108. Upper and lower jaw trabeculae (UKW75): (0) well developed; (1) weakly developed.

109. Second transverse keel (MKDK99): (0) absent; (1) present.

110. Calcification pattern of radials (MKDK100): (0) catenated; (1) crustal.

111. Mid-dorsal surface of disc covered by heart-shaped denticles arranged in an antero-posteriorly directed patch having sharply defined outlines (MKDK102): (0) absent; (1) present.

112. File of enlarged 'caniniform' teeth in the upper jaw (MCNK103): (0) absent; (1) present.

113. *Compagibus laminam* (new): (0) absent; (1) present. **Remarks**: This structure consists of a set of condensed propterygial radials that have interradial fin-ray joints and no terminal branching present in some pelagic stingrays (Hall *et al.* 2018). It is found only in the anterior portion of the pectoral fins of *Aetomylaeus*, *Aetobatus*, *Rhinoptera*, and *Mobula* (Hall *et al.* 2018), whereas it is lacking in *Myliobatis californica* and *M. goodei*. As the authors reported *Myliobatis freminvillei* as showing an intermediate morphology, we prefer to code it as unknown/dubious (?) pending further analyses. Among fossil holomorphic taxa, this feature is lacking (0) in †*Dasyomyliobatis* and †*Promyliobatis* (Marramà *et al.* 2019*b*), whereas its presence is unclear in †*Weissobatis* (?).

114. Index of fin-ray distribution, FRD (new): (0) negative; (1) positive. **Remarks**: Hall *et al.* (2018) proposed this index in order to illustrate evolutionary trend in the distribution of pectoral-fin rays, in which propterygial radials decrease in number with respect to the metapterygial rays in concomitance with shift toward the pelagic lifestyle. The index is calculated as the number of metapterygial- minus propterygial radials, divided by the total number of pectoral-fin radials. The authors found that negative index usually characterizes benthic stingrays with undulatory swimming mode (0) with the exceptions of *Dasyatis* and *Taeniura* (whose species can show both positive or negative index (0&1), whereas *Neotrygon* and *Pastinachus* show positive index (1). †*Dasyomyliobatis*, *Gymnura* and all aquilopelagic stingrays show positive index (1).

115. Pectoral-fin aspect ratio (new): (0) AR<1.0; (1) 1.0<AR<2.0, (2) 2.0<AR<3.0, (3) AR>3.0. **Remarks**: Pectoral-fin aspect ratio (AR) is an indicator of functionality whose values and variations in different taxa can be calculated to make inferences of potential swimming and lifestyle diversity (Martinez *et al.* 2016). It is calculated as the maximum chord width of the pectoral-fin squared, divided by its surface area; the obtained value is therefore doubled for the single-calculated fin to get the estimated AR value for both fins (Martinez *et al.* 2016). AR is strongly related to disc shape, swimming mode and environmental preferences (Martinez *et al.* 2016): species with very low AR (<1.0) have rounded pectoral disc and fins suited for fine-scale manoeuvrability within their riverine (*Heliotrygon*, *Potamotrygon*, *Paratrygon*, *Plesiotrygon*, †*Heliobatis*, †*Asterotrygon*) or marine (†*Lessiniabatis*) environments; outgroups and most of marine benthic demersal stingrays with 1.0<AR<2.0 have subcircular to rhombic pectoral-disc shape (1), whereas †*Weissobatis*, *Aetomylaeus*, *Myliobatis*, *Aetobatus*, *Rhinoptera* and *Mobula* (all aquilopelagic stingrays having a pelagic lifestyle and crescent-shaped pectoral-fin with a convex leading edge and concave trailing edge) have very high AR (>3.0). Interestingly, †*Promyliobatis* and †*Dasyomyliobatis* have intermediate ratio (2.0<AR<3.0) similar to *Gymnura*, a peculiar stingray closely related to benthic stingrays but sharing some myliobatoid features (e.g. wing-like disc stiffened by crustal-calcified radials and cross-braces). *Gymnura* is peculiar also in its lifestyle as it can shift between more undulatory and oscillatory swimming when in benthic and pelagic environments, giving this taxon unique hybrid swimming capabilities (Martinez *et al.* 2016). Having similar AR, as well as a mix of dasyatoid and myliobatoid trats, we predict that †*Dasyomyliobatis* could have had similar hybrid swimming capabilities and environmental preferences. The calculated AR values for all holomorphic taxa are shown in Table S3.
116. Head position (LLCL3): (0) head protruding from pectoral disc; (1) head not protruding from pectoral disc but resting inside it.
117. Tail folds (new): (0) both dorsal and ventral folds present; (1) only ventral fold present. **Remarks** (see also ch. 37): In those taxa in which caudal fin is not completely developed but reduced to tail

folds, it is possible to detect variation in the presence/absence of the dorsal fold. †*Asterotrygon*, *Dasyatis*, *Potamotrygon*, †*Tethytrygon*, and *Neotrygon* have small radials sustaining both dorsal and ventral folds (0) (De Carvalho *et al.* 2004; Last *et al.* 2016; Marramà *et al.* 2019*a*). Conversely, in *Pteroplatytrygon*, *Styracura*, *Plesiotrygon*, *Heliotrygon*, *Taeniura*, *Pastinachus*, and †*Dasyomyliobatis* only the ventral fold is present (1).

118. Number of labio-lingually directed tooth files on jaws (new): (0) 20 or more; (1) 19-8; (2) 7-5; (3) one. **Remarks**: outgroups and most of benthic stingrays usually have a mid-high number of tooth files (antero-posterior rows), between 20 to 60 in both upper and lower jaws (0) (Nishida 1990; Cappetta 2012). This plesiomorphic condition is also characteristics of †*Dasyomyliobatis.* Stingrays went toward reduction in number of files concomitant with the shift toward durophagy (Cappetta 2012). As detected by rare fossil tooth plates, a first possible reduction to 19-8 tooth files (1) seems to have occurred in the earliest fossil taxa with complete polyaulacorhizy like †*Apocopodon* (de Santana *et al.* 2011). In †*Hypolophites*, †*Igdabatis* and †*Brachyrhizodus*, being represented by isolated teeth only or incomplete dental plates (Cappetta 2012), the condition is difficult to determine (?). Further reduction to 7-5 teeth (one large symphysial and six or four small lateral) is typical of living *Myliobatis* and *Aetomylaeus*, and fossil taxa †*Promyliobatis* (Marramà *et al.* 2019*b*), †*Weissobatis* (Hovestadt & Hovestadt-Euler 1999), †*Myliobatis toliapicus*, †*M. dixoni*, †*M. striatus*, and †*M. toliapicus* (Hovestadt & Hovestadt-Euler 2013). Extreme reduction occurs in *Aetobatus*, which possess only a single file of symphyseal teeth and no lateral (3). It has been demonstrated that in durophagous stingrays, a first reversal, consisting in increase in number of tooth files, occurred in *Rhinoptera* and *Bunrhamia* (1) that will give origin during the Paleogene to planktivorous taxa (Mobulidae) (Underwood *et al.* 2017; Adnet *et al.* 2019) characterized by further extreme increase with more than 100 tooth files (0).
119. Ornamentation of the occlusal tooth surface (BLA66): (0) absent; (1) present.
120. Ornamentation of the occlusal tooth surface (BLA66, modified): (0) puckered or pitted; (1) granular to vermiculate.

121. Rostral cartilage (VSM4): (0) well-developed rostral plate with variation degrees of contribution from the lamina orbitonasalis; (1) reaches the tip of the snout (pectoral fin carry); (2) reaches the tip of the snout (growth of lamina orbitonasalis to support pectoral fins).

122. Well-developed anterior ventro-lateral process of Meckel's cartilage: (0) absent; (1) present. **Remarks**: According to Stepanek & Kriwet (2015) *Paratrygon* and *Potamotrygon* possess a well-developed anterior ventro-lateral process of Meckel's cartilage which, along with the anterior ventro-medial process, forms the joint socket of the Meckel's cartilage, which encloses the head of the palatoquadrate. This character, although less pronounced, appears to be present also in *Styracura*, *Heliotrygon*, and *Plesiotrygon* (De Carvalho & Lovejoy 2011; Stepanek & Kriwet 2015; De Carvalho *et al.* 2016), corroborating the hypothesis of the monophyly of Potamotrygonidae. Interestingly, a well-developed anterior ventro-lateral process of Meckel's cartilage is also present in †*Lessiniabatis* (Marramà *et al.* 2019*c*, fig. 3) suggesting close relationship with members of this family, as evidenced by similar body plan (Kolmann *et al.* 2022).

123. Joint staggering (new): (0) absent; (1) present. **Remarks**: according to Schaefer & Summers (2005) in some undulatory-swimming stingrays, the joints between the radials in the most distal aspects of the wing are arranged in a lattice-like network such that any joint would be opposed on either side by the middle of a radial. This pattern, called 'joint staggering', was detected at the edge of the wing after the first radial bifurcation in all *Dasyatis* species, *Himantura*, *Styracura*, and *Taeniura* (Schaefer & Summers 2005). Joint staggering is absent in all other stingrays, including †*Dasyomyliobatis*.

124. Type of dentition (sensu Cappetta 2012) (new): (0) crushing type; (1) crushing-grinding type; (2) grinding type. **Remarks**: crushing-type dentition is typical of batoids with batteries of narrowly imbricated small teeth arranged in a high number of both antero-posterior files and functional mesio-distal rows. The dental crown is usually bulging with often a transverse keel either smooth (e.g. Rajidae) or more often with puckered or pitted enameloid (e.g. Dasyatidae). Tooth batteries

do not produce a flat surface, but rather an embossed surface. Crushing-type dentition is typical of benthic demersal batoids (teeth of male individuals being characterized by high cusp can be also considered of clutching-type), including *Raja*, *Rhinobatos*, and most of rajobenthic stingrays (0). Grinding-type dentition (2) consists of teeth with high crown and polygonal (tetra- to hexagonal) outline. Teeth are very narrowly imbricated forming robust dental plate with a nearly flat surface, useful to crush hard prey with resistant shells like bivalves, gastropods, and crustaceans. Grinding-type dentition occurs in Recent and fossil forms with polyaulacorhizous roots (2), except for planktivorous stingrays, which possess teeth of clutching (*Mobula birostris*) or crushing (*Mobula hypostoma*) type (0) (Cappetta 2012). An intermediate stage called crushing-grinding type (1) has been proposed by Cappetta (2012) to describe an peculiar dentition in which a moderate to marked monognathic heterodonty with some teeth files of crushing type, but the majority are of grinding type (Cappetta 2012; Adnet *et al.* 2019). Dignathic heterodonty is marked by the grinding complex between teeth of pestles (curved upper lateral teeth) and teeth of crucibles (antero-lateral lower teeth). Whereas this mixing between crushing and grinding types is particularly notable in upper jaw, the lower jaw exhibits a more uniform tooth plate. Crushing-grinding dentition has been observed in fossil and living *Pastinachus* species, and Late Cretaceous to early Paleogene taxa with holaulacorhizous teeth only, or with both holaulacorhizous and polyaulacorhizous teeth, including †*Hypolophites*, †*Meridiania*, †*Hypolophodon*, †*Myliodasyatis* (Cappetta 2012; Adnet *et al.* 2019). In this perspective, we consider also †*Dasyomyliobatis* and †*Brachyrhizodus* as having this kind of dentition (1).

APPENDIX S2

Institutional abbreviations

CAS, California Academy of Sciences (https://www.calacademy.org/); **CMNH**, Carnegie Museum, Pittsburgh; **CSIRO**, Australian National Fish Collection (https://www.csiro.au/); **CToL**, CT-scans from the Chondrichthyan Tree of Life Project (http://www.sharksrays.org); **FishBase** (http://www.fishbase.org/); **FMNH**, Zoological Collection of the Filed Museum (https://collections-zoology.fieldmuseum.org/); **IUWP**, Department of Palaeontology of the University of Vienna; **MB.F**, Museum für Naturkunde, Berlin; MGGC, Museo Geologico Giovanni Capellini, Università degli Studi di Bologna; **MGP-PD**, Museo di Geologia e Paleontologia dell'Università degli Studi di Padova; **MCSNV**, Museo Civico di Storia Naturale di Verona; **MCZ**, Museum of Comparative Zoology, Harvard University; **MFSN** Museo Friulano di Storia Naturale in Udine; **MNHN**, Ichthyological collection of Museum National d'Histoire Naturelle, Paris; **MSNFI,** Istituto di Geologia del Museo di Storia Naturale dell'Università di Firenze; **MSNP**, Museo di Storia Naturale dell'Università degli Studi di Pavia; **NHMUK,** Natural History Museum London (https://www.nhm.ac.uk/); **NHMW**, Naturhistorisches Museum Wien, Wien, Austria; **RGM**, Rijksmuseum van Natuurlijke Historie (Netherlands Centre for Biodiversity Naturalis); **USNM**, Smithsonian National Museum of Natural History (https://collections.nmnh.si.edu/).

Comparative material

Aetobatus narinari: MNHN-IC-A-7948; 3D Volume Rendering Technique (VRT) skull reconstruction from CT scans (no catalogue number) by Prof. Frank Fish (West Chester University); *Aetomylaeus nicholfi*: (Jabado *et al.* 2022); †*Arechia crassicaudata*: MCSNV IG.VR.27607, MCSNV VII.B.82/83, MGP-PD 8875C/76C, MCSNV T.317/318, MCSNV VII.B.84/85, NHMW 1853.0027.0005 (Marramà *et al.* 2020); *Dasyatis americana* and *D. zugei*: CT-scans from CToL; *Dasyatis pastinaca*: FishBase radiographs; †*Dasyomyliobatis thomyorkei*: MCSNV VR.21.107/8 (this paper); *Fluvitrygon signifier*: IPUW Chond-T-23; *Gymnura altavela*: CT-scan from CToL; *Gymnura marmorata*: (CAS) SU11587; *Hexatrygon bickelli*: CAS233779; *Himantura uarnak*: (NMNH) RAD110908-001; †*Lessiniabatis aenigmatica*: MNHN F.Bol.566, MFSN GP.864, MSNFI

IGF103555; *Mobula munkiana*: CT-scan from CToL; *Myliobatis goodei*: USNM9524; *Neotrygon kuhlii*: IPUW 7355, NHMUK 2015.1.25.6 (CT scan renders provided by C. Underwood); CT-scans from CToL; *Paratrygon aiereba*: FMNH 84092; *Pastinachus sephen*: MNHN IC-A-7942; *Platyrhina sinensis*: MNHN IC.0000.1307, USNM 51295, USNM 86920, USNM 192562; *Plesiobatis daviesi*: CSIRO CA4238; †*Plesiozanobatus egertoni*: MGP-PD 154Z, MCSNV IG.43347, MB.f 1608.1/2, MCSNV IG.142530, MCSNV VII.B.80/81, MCSNV VII.B.88/89; *Potamotrygon humboldti*: NMNH 168602; *Potamotrygon motoro*: CT-scan from CToL; *Potamotrygon tigrina*: IUWP 7361; †*Promyliobatis gazolai*: MCSNV VII.B.90/91; MSNPV 14620 (Marramà *et al.* 2019*b*); †*Protohimantura vorstmani*: RGM 624420; *Pteroplatytrygon violacea*: (Last *et al.* 2016); *Rhinoptera bonasus*: CT-scan from CToL; *Rhinoptera* sp., IUWP uncatalogued, dissected specimen; *Taeniura lymma*: USANM 170359, IUWP, uncatalogued specimen; †*Tethytrygon muricatus*: MNHN F.Bol.564, MGP-PD 159/160, MGP-PD 150Z/151Z, CMNH 4521, MCSNV IG.23194, MCSNV IG.186653, MCSNV T.1020/1, MCSNV VII.B.92/3, MCZ 13183, MGGC 7456, MNHN F.Bol.568, MNHN F.Bol.584; *Trygonoptera testacea*: (CAS) SU 9169; *Urobatis halleri*: (CAS) SU 2948; *Urolophus aurantiacus*: CT-scan from CToL; *Urolophus kaianus*: NHMUK 1879.5.14.424; *Urotrygon chilensis*: CT-scan from CToL; *Urotrygon rogersi*: (CAS) SU 11700; †*Weissobatis micklichi*: (Hovestadt & Hovestadt-Euler 1999); *Zanobatus schoenleinii*: CT-scan from CToL.

Images used for geometric morphometrics and aspect ratio (AR) calculation

Aetobatus narinari: MNHN-IC-A-7948; *Aetomylaeus nicholfi*: image from Jabado *et al.* (2022); †*Arechia crassicaudata*: image from Marramà *et al.* (2020); †*Asterotrygon maloneyi*: image from De Carvalho *et al.* (2004); †*Dasyomyliobatis thomyorkei*: MCSNV VR.21.107/8; *Dasyatis helleri*: image from FishBase; *Gymnura marmorata*: (CAS) SU 11587; †*Heliobatis radians*: image from De Carvalho *et al.* (2004); *Heliotrygon gomesi*: image from De Carvalho & Lovejoy (2011); *Hexatrygon bickelli*: CAS 233779; *Himantura uarnak*: USNM RAD110908-001; †*Lessiniabatis aenigmatica*: image from Marramà *et al.* (2019*c*); *Mobula birostris* and *Mobula hypostoma*: images from Last *et*

al. (2016); *Myliobatis aquila*, *M. californicus*, and *M. freminvillei*: images from Last *et al.* (2016); *Myliobatis goodei*: USNM 9524; *Neotrygon kuhlii*: CT-scan image from CtoL; *Paratrygon aiereba*: FMNH 84092; *Pastinachus sephen*: MNHN IC-A-7942; *Plesiobatis daviesi*: CSIRO CA4238; *Plesiotrygon nana*: image from De Carvalho & Ragno (2011); *Potamotrygon humboldti*: NMNH 168602; †*Promyliobatis gazolai*: image from Marramà *et al.* (2019*b*); *Pteroplatytrygon violacea*: image from Last *et al.* (2016); *Rhinoptera bonasus*: image from Last *et al.* (2016); *Styracura schmardae*: image from De Carvalho *et al.* (2016); *Taeniura lymma*: NMNH 170359; †*Tethytrygon muricatus*: image from Marramà *et al.* (2019*a*); *Trygonoptera halleri* (CAS) SU 9169; *Urobatis halleri*: (CAS) SU 2948; *Urolophus bucculentus*: images from Last *et al.* (2016); *Urotrygon rogersi*: (CAS) SU 11700; †*Weissobatis micklichi*: image from Hovestadt & Hovestadt-Euler (1999).

APPENDIX S3

Detailed results of the phylogenetic analyses

Parsimony analyses. The Parsimony analysis performed on the whole dataset (124 traits coded for 52 living and both fossil holomorphic and tooth-based taxa) produced a single parsimonious tree having length of 311 steps, CI of 0.538, and RI of 0.847 (Fig. S1). The monophyly of the Myliobatiformes (sensu Villalobos-Segura *et al.* 2022) (including *Zanobatus* and †*Plesiozanobatus*) is moderately supported (Bremer value 2) by five synapomorphies, including hyomandibulae articulating with lower jaws through strong, stout ligament (hyomandibular-Meckelian ligament) at distal tip (13[1]), basihyal a single element, but separate from first hypobranchials (20[1]), rostral cartilage inconspicuous (81[1]), ball and socket articulation between scapular process and synarcual (86[1]), and dorsal marginal clasper cartilage having medial flange (91[1]). Conversely, the monophyly of Myliobatiformes sensu stricto (the stingrays, excluding *Zanobatus* and †*Plesiozanobatus*) is more strongly supported (Bremer value 5) by nine synapomorphies: scapular loops formed by scapular components of trunk lateral line canals (4[1]), nasal capsules ventrolaterally expanded (12[1]), dorsal fin absent (34[1]), presence of serrated tail stings (72[1]), infraorbital loop

of suborbital and infraorbital canals (79[1]), rostral cartilage inconspicuous (82[1]), jugal arch absent (83[1]), presence of a thoracolumbar synarcual (87[1]), spiracularis splits into lateral and medial bundles, with the medial bundle inserting onto the posterior surface of Meckel's cartilage and the lateral bundle inserting onto the dorsal edge of the hyomandibula (97[1]).

The tree topology does not show the dichotomous nature of remaining myliobatiforms as determined by Marramà *et al.* papers (Marramà *et al.* 2019*a*, *c*, 2020), but is rather more congruent with the consensus trees recovered by De Carvalho *et al.* (2004) and Claeson *et al.* (2010) in detecting the aquilopelagic stingrays as a very derived clade. *Plesiobatis* is recovered as the sister to all the other stingrays, contrary to the hypotheses inferred by most analyses based on morphological data (De Carvalho *et al.* 2004; Claeson *et al.* 2010; Aschliman *et al.* 2012; Marramà *et al.* 2019*a*, 2020) in which *Hexatrygon* is usually recovered as the basalmost stingray. Conversely, this latter is sister to *Gymnura* and this pair is closely related to urolophids, more similarly to some recent molecular phylogenies (Naylor *et al.* 2012; Bertozzi *et al.* 2016). The clade (Urolophidae+(*Gymnura*+*Hexatrygon*)) is supported by three characters: absence of placoid scales and thorns (73[1], 75[1]), and rostral cartilage that reaches the tip of the snout (pectoral fin carry) (121[1]). This clade forms a polytomy with (*Asterotrygon*+*Heliobatis*) and all the remaining stingrays. Urotrygonids, potamotrygonids and dasyatids are successive sister groups to *Pastinachus* and the lineage that will give origin to durophagous pelagic stingrays (already described in detail in the main manuscript). The monophyly of the family Urotrygonidae (*Urotrygon*+*Urobatis*) is weakly supported (Bremer value 1) by tubules of subpleural components of hyomandibular lateral line canals dichotomously branched at extremities (1[1]) and by the presence of spiracular tentacle (44[1]). The Potamotrygonidae include *Styracura* as sister to *Heliotrygon*, †*Lessiniabatis*, *Paratrygon*, *Plesiotrygon* and *Potamotrygon*, whose monophyletic relationship is supported (Bremer value 1) by the presence of a well-developed anterior ventro-lateral process of Meckel's cartilage(Stepanek & Kriwet 2015). Dasyatidae (excluding *Pastinachus*) is supported by the presence of a ventral terminal cartilage that is free of an axial cartilage (96[1]), sexual dental heterodonty (98[1]), and joint

staggering (123[1]). The relationships of the clade formed by dasyatids and remaining stingrays is explained in detail in the main manuscript.

The Parsimony analysis performed on the reduced matrix (including living and holomorphic fossil stingrays only; 124 traits coded for 40 taxa) to detect traits that characterized the shift toward oscillatory swimming and pelagic lifestyle, produced three equally parsimonious trees that were used to build the 50% Majority Rule tree (Fig. S2). Compared to the tree detected from the one based on the whole dataset, the 50% MR tree has an identical tree topology, similar statistics (286 steps, CI of 0.570, and RI of 0.831), and similar family-level relationships with most of nodes supported by the same characters. Dasyatids plus remaining stingrays show positive FRD suggesting that dasyatids already face toward reduction of the number of propterygial radials, that will be furtherly reduced in aquilopelagic taxa. The node including †*Dasyomyliobatis* as sister to all aquilopelagic stingrays is supported by the appearance of cephalic lobes (45[1]) and increase in AR, with intermediate value between those of rajobenthic and aquilopelagic stingrays (115[1>2]). Although the head already extends anterior to pectoral disc in †*Dasyomyliobatis*, this state (116[0]) is not detected on node, likely because it is not present in †*Promyliobatis* in which the heard rests inside the pectoral disc, possibly representing a reversal. The following step (node including †*Promyliobatis* sister to remaining stingrays) defines the achievement of the crustal calcification (110[1]) coupled with cross-bracing (30[1]), and loss of caudal fin (36[1]). All remaining stingrays achieve the final and definitive crescent pectoral-disc shape, highlighted by the increase in AR (>3.0; 115[2>3]).

Bayesian analysis. The Bayesian topology obtained using the whole dataset matrix (124 traits coded for 52 taxa) has the following statistics: Arithmetic mean -1345.85, Harmonic mean -1376.61 CI 0.502, RI 0.826 (Fig. S3). *Plesiobatis*, urolophids, and the sister groups (*Hexatrygon*+*Gymnura*) and (*Urotrygon*+*Urobatis*) fall in a polytomous relationship with remaining stingrays. †*Asterotrygon*+†*Heliobatis*, †*Lessiniabatis*, and living potamotrygonids are successive sisters of a paraphyletic Dasyatidae. However, the evolutionary steps giving rise to aquilopelagic stingrays closely resemble those recovered in the Parsimony analysis, since *Pastinachus,* †*Hypolophites,*

†*Dasyomyliobatis,* †*Brachyrhizodus,* †*Apocopodon,* †*Igdabatis,* †*Myliobatis wurnoensis,* †*M. dixoni,* †*Promyliobatis*, and †*Weissobatis* are successive sister taxa to all remaining aquilopelagic stingrays, therefore suggesting that the characters employed in our analysis are quite robust, that the resulting systematic arrangement is very stable, and that the hypothesis of gradual achievement of traits supporting the evolutionary origin of the durophagous pelagic ecomorph appears reliable. Finally, *Aetomylaeus*, living *Myliobatis* species, †*M. toliapicus* and †*M. striatus* form a polytomy along with the clade formed by †*Sulcidens*+(*Aetobatus*+(*Rhinoptera*+(†*Burhnamia*+*Mobula*))).

Maximum Likelihood analysis. The Maximum Likelihood topology obtained using the whole dataset (124 traits coded for 52 taxa) (Maximum likelihood score 1293.99, CI 0.516, RI 0.836; Fig. S3) closely resembles that recovered in the Bayesian analysis, but the tree appears better resolved. *Hexatrygon* and *Gymnura* are successive sisters to the remaining stingrays. Although the clade consisting of *Urotrygon* and *Urobatis* is still recovered, it falls into a polytomy with †*Arechia* and [*Urolophus*+*Trygonoptera*], so that Urolophidae including †*Arechia* is not recovered any more. Although Dasyatidae and Myliobatidae were recovered paraphyletic, *Pastinachus,* †*Hypolophites,* †*Dasyomyliobatis,* †*Brachyrhizodus,* †*Apocopodon,* †*Igdabatis,* †*Myliobatis wurnoensis,* and †*M. dixoni* are still successive sister to all remaining stingrays, therefore corroborating the reliability of the evolutionary steps giving rise to the aquilopelagic ecomorph.

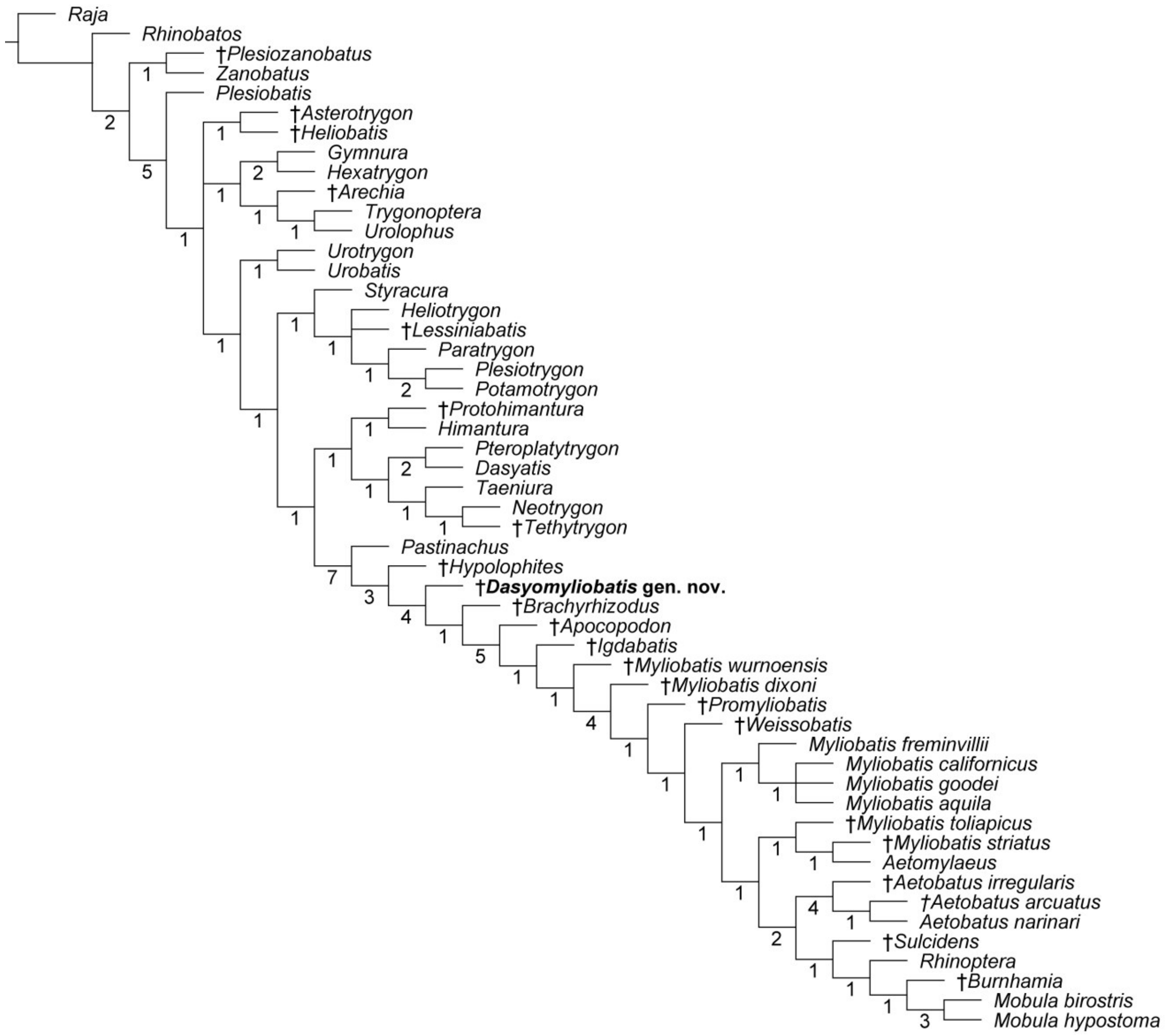

FIG. S1. The single parsimonious tree retrieved in TNT v.1.5 based on 124 morphological characters and 52 taxa showing the hypothetical relationships of †*Dasyomyliobatis thomyorkei* gen. et sp. nov. within the Myliobatiformes. Numbers on nodes indicate the Bremer support. Extinct taxa are marked with a dagger.

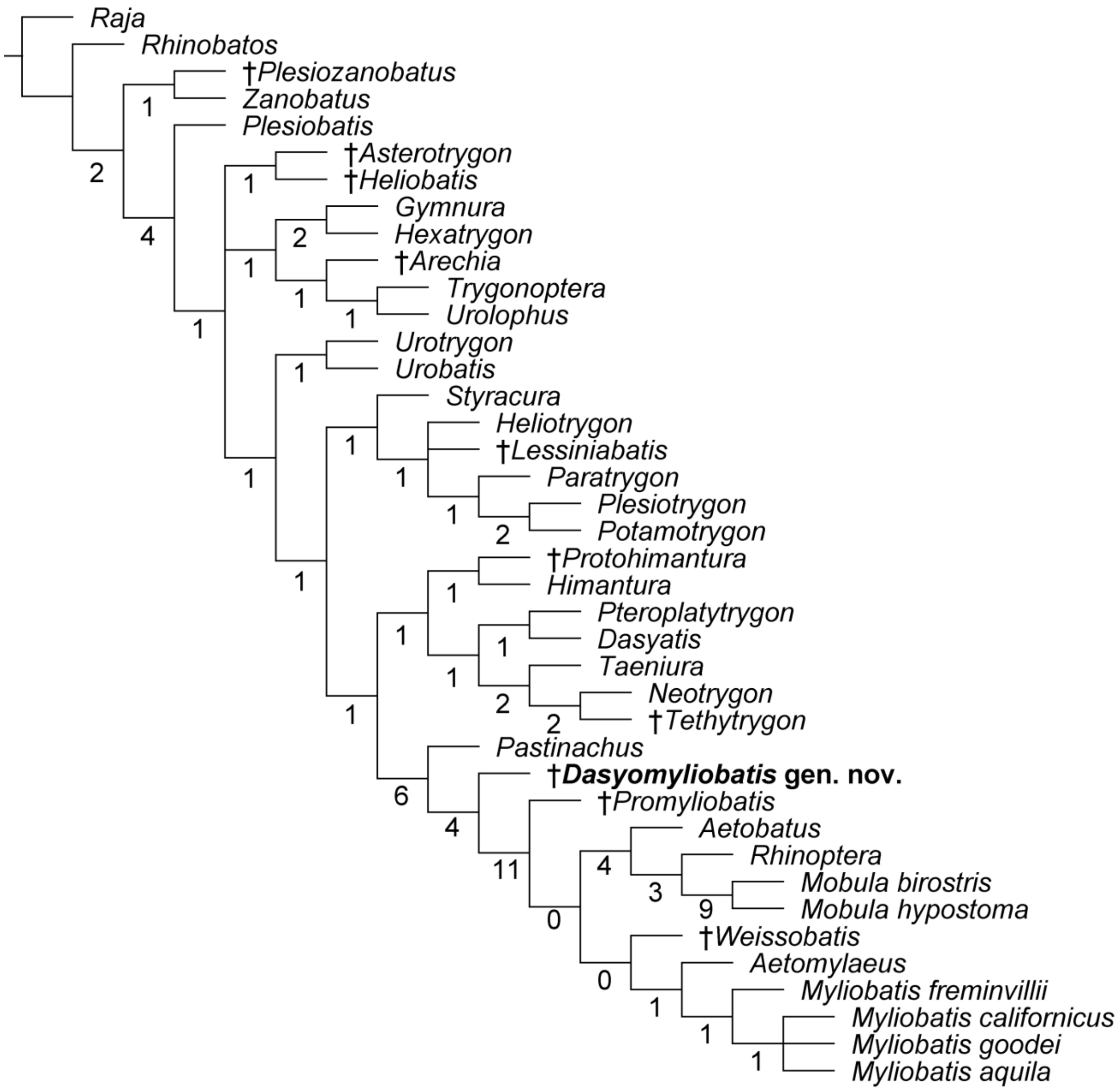

FIG. S2. The 50% Majority Rule tree retrieved in TNT v.1.5, based on 124 morphological characters and 40 holomorphic living and fossil taxa, and showing the hypothetical relationships of †*Dasyomyliobatis thomyorkei* gen. et sp. nov. within the Myliobatiformes. Numbers on nodes indicate the Bremer support. Extinct taxa are marked with a dagger.

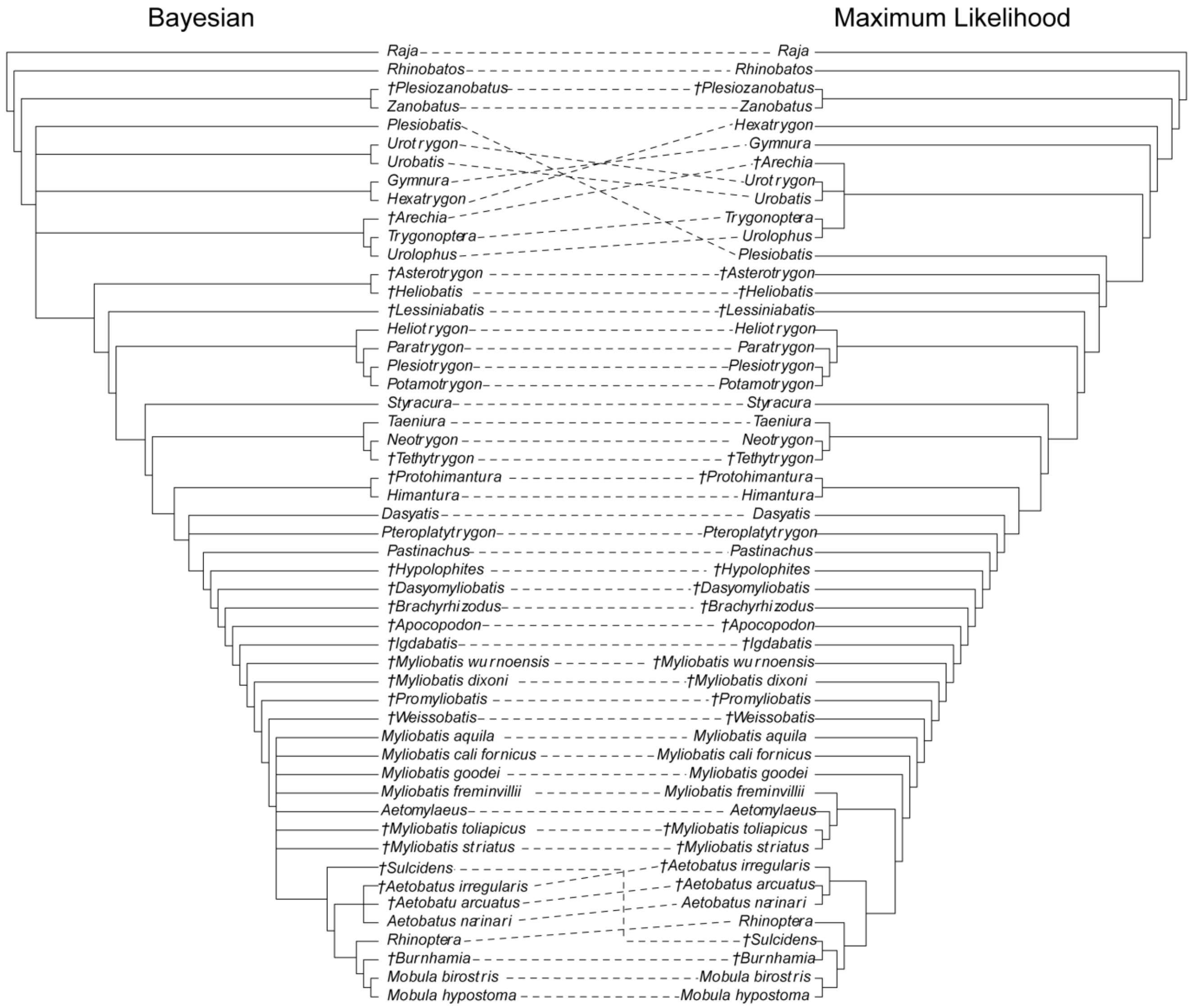

FIG. S3. Comparison between Bayesian and Maximum Likelihood tree topologies based on the whole dataset. Extinct taxa are marked with daggers.

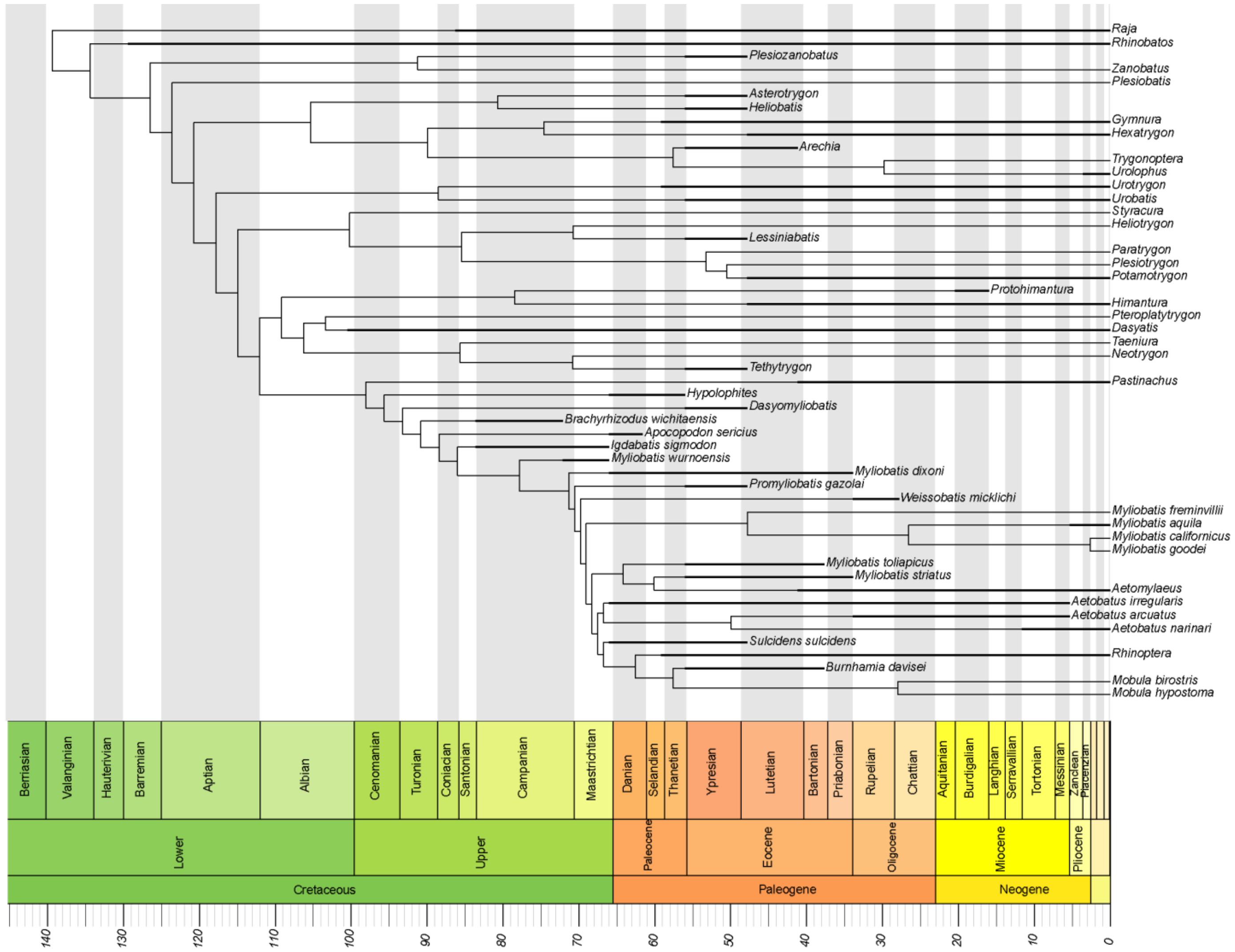

FIG. S4. Time-calibrated tree based on 124 morphological characters and 52 taxa.

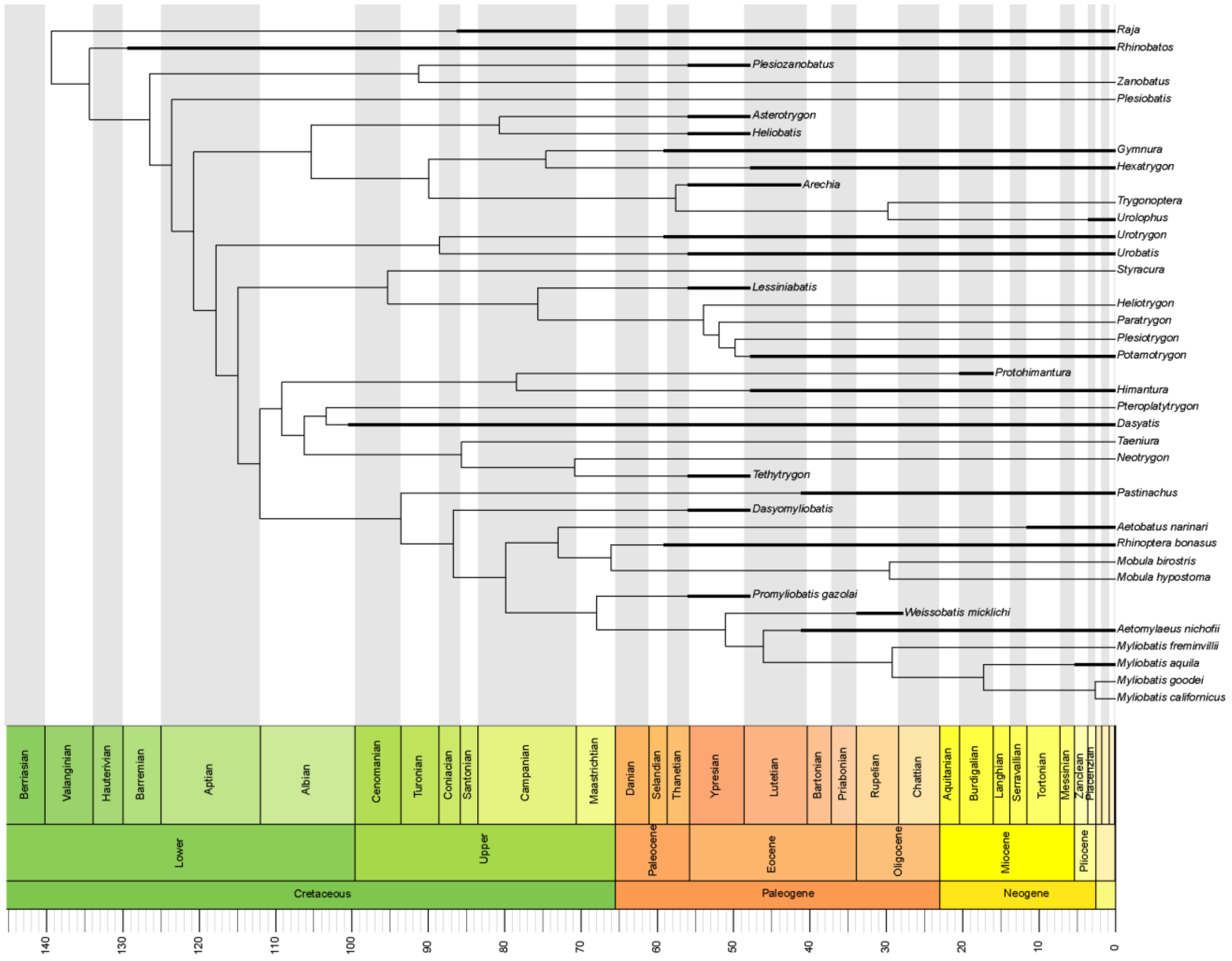

FIG. S5. Time-calibrated tree based on 124 morphological characters and 40 holomorphic living and fossil taxa.

TABLE S1. Age estimates for the individual MCSNV VR.21.107/8 of †*Dasyomyliobatis thomyorkei* gen. et sp. nov. using the von Bertalanffy functions of the closest living stingray relative species. *k* = growth coefficient (rate of change in length increment); maxDW = mean maximum disc width for the population (in cm); DW_{50} = disc width at maturity (in cm); t_0: hypothetical postnatal length; C: male and female combined; F: female. Age is in years.

Taxon	Sex	k	t_0	max DW (cm)	DW_{50} (cm)	Age at maturity	Age of MCSNV VR.21.107/8 (von Bertalanffi)	Uncertainty as standard errors (SE)	Sources
Dasyatis pastinaca	F	0.086	-1.24	188.5	62.5	7.0	7.54	±0.3	A
Dasyatis pastinaca	F	0.041	-2.61	219.9	49	7.0	12.16	±2.6	B
Myliobatis californica	F	0.0995	-2.06	158.7	88.1	5.0	7.92	±1.5	C
Rhinoptera bonasus	F	0.119	-3.76	125.0	92	7.5	9.73	±1.1	D
Aetomylaeus bovinus	F	0.056	-1.90	242.6	95	6.95	7.57	±0.3	E
Aetobatus narinari	F	0.0313	-7.04	245.9	124	15.4	9.61	±2.9	F, G
Pastinachus ater	C	0.12	-1.25	167.0	100.2	6.0	6.35	±0.2	H,
Pastinachus sephen	C	0.12	-1.25	183.0	98	6.0	5.33	±0.3	G, H

Sources: A, Yigin & Ismen (2012); B, Yeldan *et al.* (2009); C, Martin & Cailliet (1988); D, Smith & Merriner (1987); E, Başusta & Aslan (2018); F, Dubick (2000); G, Froese & Pauly (2022); H, O'Shea *et al.* (2013).

TABLE S2. Taxon first appearance datum (FAD) and last appearance datum (LAD) used for time-calibrations of phylogenetic analyses.

Taxon	FAD	LAD	Reference
Aetobatus arcuatus	33.9	5.3	Claeson *et al*. 2010
Aetobatus irregularis	66	5.3	Claeson *et al*. 2010
Aetobatus narinari	11.6	0	Claeson *et al*. 2010
Aetomylaeus	41.2	0	Villafaña *et al*. 2020
Apocopodon sericius	66	61.6	Cappetta 2012
Arechia	56	41.2	Cappetta 2012
Asterotrygon	56	47.8	De Carvalho *et al*. 2004
Brachyrhizodus	83.6	72.1	Cappetta 2012
Burnhamia davisei	56	37.7	Underwood *et al*. 2017
Dasyatis	100.5	0	Cappetta 2012
Dasyomyliobatis	56	47.8	this paper
Gymnura	59.2	0	Cappetta 2012
Heliobatis	56	47.8	De Carvalho *et al*. 2004
Heliotrygon	0	0	
Hexatrygon	47.8	0	Cappetta 2012
Himantura	47.8	0	Marramà *et al*. 2018
Hypolophites	66	56	Cappetta 2012
Igdabatis sigmodon	83.6	66	Cappetta 2012
Lessiniabatis	56	47.8	Marramà *et al*. 2019*c*
Mobula birostris	0	0	Adnet *et al*. 2012; Underwood *et al*. 2017
Mobula hypostoma	0	0	
Myliobatis aquila	5.3	0	Claeson *et al*. 2010
Myliobatis californicus	0	0	
Myliobatis dixoni	66	33.9	Claeson *et al*. 2010
Myliobatis freminvillii	0	0	
Myliobatis goodei	0	0	
Myliobatis striatus	56	33.9	Claeson *et al*. 2010
Myliobatis toliapicus	56	37.7	Claeson *et al*. 2010
Myliobatis wurnoensis	72.1	66	Claeson *et al*. 2010
Neotrygon	0	0	
Paratrygon	0	0	
Pastinachus	41.2	0	Adnet *et al*. 2019
Plesiobatis	0	0	
Plesiotrygon	0	0	
Plesiozanobatus	56	47.8	Marramà *et al*. 2020
Potamotrygon	47.8	0	Cappetta 2012
Promyliobatis gazolai	56	47.8	Marramà *et al*. 2019*b*
Protohimantura	20.4	16	Marramà *et al*. 2018
Pteroplatytrygon	0	0	
Raja	86.3	0	Cappetta 2012
Rhinobatos	129.4	0	Cappetta 2012
Rhinoptera	59.2	0	Adnet *et al*. 2012; Underwood *et al*. 2017
Styracura	0	0	
Sulcidens sulcidens	66	47.8	Underwood *et al*. 2017
Taeniura	0	0	
Tethytrygon	56	47.8	Marramà *et al*. 2019*a*
Trygonoptera	0	0	
Urobatis	56	0	Cappetta 2012
Urolophus	3.6	0	Cappetta 2012
Urotrygon	59.2	0	Cappetta 2012
Weissobatis micklichi	33.9	27.8	Hovestadt & Hovestadt 1999
Zanobatus	0	0	

TABLE S3. The Aspect Ratio (AR) calculated for 35 taxa in order to investigate its relationship with the pectoral-fin shape detected through geometric morphometrics. These data were also used for phylogenetic and geometric morphometrics analyses (see *Character list*). Extinct taxa are marked with dagger symbol.

Taxon	Aspect Ratio (AR)
Aetobatus narinari	4.03
Aetomylaeus nicholfi	3.60
†*Arechia crassicaudata*	1.01
†*Asterotrygon maloneyi*	0.95
Dasyatis pastinaca	1.74
†*Dasyomyliobatis thomyorkei*	2.69
Gymnura marmorata	2.82
†*Heliobatis radians*	0.78
Heliotrygon gomesi	0.72
Hexatrygon bickelli	1.14
Himantura uarnak	1.41
†*Lessiniabatis aenigmatica*	0.58
Mobula birostris	5.86
Mobula hypostoma	3.93
Myliobatis aquila	3.69
Myliobatis californicus	3.77
Myliobatis freminvillei	3.44
Myliobatis goodei	3.44

Taxon	Aspect Ratio (AR)
Neotrygon kuhlii	1.99
Paratrygon aiereba	0.66
Pastinachus sephen	1.65
Plesiobatis daviesi	1.10
Plesiotrygon nana	0.98
Potamotrygon humboldti	0.94
†*Promyliobatis gazolai*	2.68
Pteroplatytrygon violacea	1.73
Rhinoptera bonasus	4.81
Styracura schmardae	1.15
Taeniura lymma	1.06
†*Tethytrygon muricatus*	1.27
Trygonoptera testacea	1.41
Urobatis halleri	1.09
Urolophus bucculentus	1.52
Urotrygon rogersi	1.58
†*Weissobatis micklichi*	5.39

TABLE S4. Results of PERMANOVA and ANOSIM shown as post-hoc tests. The significance is computed by permutation of group membership with 9,999 replicates. Asterisk (*) indicates significant comparisons (p<0.05) between ecomorphotypes.

**PERMANOVA**				**ANOSIM**			
Permutation N:	9,999			Permutation N:	9,999		
Total sum of squares:	2.414			Mean rank within:	146.2		
Within-group sum of squares:	0.499			Mean rank between:	432		
df	2			df	2		
F:	61.41			R:	0.9609		
p (same):	0.0001*			p (same):	0.0001*		
	**Aquilopelagic**	**Rajobenthic**	**Aquilobenthic**		**Aquilopelagic**	**Rajobenthic**	**Aquilobenthic**
**Aquilopelagic**		0.0001*	0.032*	**Aquilopelagic**		0.0001*	0.051
**Rajobenthic**	0.0001*		0.0004*	**Rajobenthic**	0.0001*		0.0003*
**Aquilobenthic**	0.032*	0.0004*		**Aquilobenthic**	0.051	0.0003*	

SUPPLEMENTARY REFERENCES

ADNET, S., CAPPETTA, H, GUINOT, G. and NOTARBARTOLO DI SCIARA, G. 2012. Evolutionary history of the devilrays (Chondrichthyes: Myliobatiformes) from fossil and morphological inference. *Zoological Journal of the Linnean Society*, **166**, 132–159.

ADNET, S., MOUANA, M., CHARRUAULT, A. L., ESSID, E. M., AMMAR, H. K., MARZOUGUI, W., MERZERAUD, G., TABUCE, R., VIANEY-LIAUD, M. and MARIVAUX, L. 2019. Teeth, fossil record and evolutionary history of the cowtail stingray Pastinachus Rüppell, 1829. *Historical Biology*, **31**, 1213–1222.

ASCHLIMAN, N. C. C., CLAESON, K. M. and MCEACHRAN, J. D. 2012. Phylogeny of Batoidea. *In* CARRIER, J. C., MUSICK, J. A. and HEITHAUS, M. R. (eds.) *Biology of Sharks and Their Relatives*, CRC Press, Boca Raton, 57–95 pp.

BAŞUSTA, N. and ASLAN, E. 2018. Age and growth of bull ray *Aetomylaeus bovinus* (Chondrichthyes: Myliobatidae) from the northeastern Mediterranean coast of Turkey. *Cahiers de Biologie Marine*, **59**, 107–114.

BERTOZZI, T., LEE, M. S. Y. and DONNELLAN, S. C. 2016. Stingray diversification across the end-Cretaceous extinctions. *Memoirs of Museum Victoria*, **74**, 379–390.

BLANCO, A. 2019. *Igdabatis marmii* sp. nov. (Myliobatiformes) from the lower Maastrichtian (Upper Cretaceous) of north-eastern Spain: an Ibero-Armorican origin for a Gondwanan batoid. *Journal of Systematic Palaeontology*, **17**, 865–879.

CAPPETTA, H. 2012. *Handbook of paleoichthyology – Chondrichthyes – Mesozoic and Cenozoic elasmobranchii: teeth*. Verlag Dr. Friedrich Pfeil, Munich.

CLAESON, K. M., O'LEARY, M. A., ROBERTS, E. M., SISSOKO, F., BOUARÉ, M., TAPANILA, L., GOODWIN, D. and GOTTFRIED, M. D. 2010. First Mesozoic record of the stingray *Myliobatis wurnoensis* from Mali and a phylogenetic analysis of myliobatidae incorporating dental characters. *Acta Palaeontologica Polonica*, **55**, 655–674.

DE CARVALHO, M. R. and LOVEJOY, N. R. 2011. Morphology and phylogenetic relationships of a remarkable new genus and two new species of neotropical freshwater stingrays from the amazon basin

(Chondrichthyes: Potamotrygonidae). *Zootaxa*, **48**, 13–48.

DE CARVALHO, M. R. and RAGNO, M. P. 2011. An unusual, dwarf new species of Neotropical freshwater stingray, *Plesiotrygon nana* sp. nov., from the upper and mid Amazon Basin: The second species of *Plesiotrygon* (Chondrichthyes: Potamotrygonidae). *Papeis Avulsos de Zoologia*, **51**, 101–138.

DE CARVALHO, M. R, MAISEY, J. G. and GRANDE, L. 2004. Freshwater stingrays of the Green River formation of Wyoming (early Eocene), with the description of a new genus and species and an analysis of its phylogenetic relationships (Chondrichthyes: Myliobatiformes). *Bulletin of the American Museum of Natural History*, **284**, 1–136.

DE CARVALHO, M. R, LOBODA, T. S. and DA SILVA, J. P. C. B. 2016. A new subfamily, Styracurinae, and new genus, *Styracura*, for *Himantura schmardae* (Werner, 1904) and *Himantura pacifica* (Beebe & Tee-Van, 1941) (Chondrichthyes: Myliobatiformes). *Zootaxa*, **4175**, 201–221.

DE SANTANA, F. R., CICIMURRI, D. J. and BARBOSA, J. A. 2011. New material of *Apocopodon sericeus* Cope, 1886 (Myliobatiformes, Myliobatidae) from the Paraíba Basin (Northeastern Brazil), and South Carolina (USA) with a reanalysis of the species. *PalArch's Journal of Vertebrate Palaeontology*, **8**, 1–20.

DUBICK, J. D. 2000. Age and growth of the spotted eagle ray, *Aetobatus narinari* (Euphrasen, 1970), from southwest Puerto Rico with notes on its biology and life history. University of Puerto Rico, 1–79 pp.

FROESE, R. and PAULY, D. 2022. *FishBase*. World Wide Web Electronic Publication.

HALL, K. C., HUNDT, P. J., SWENSON, J. D., SUMMERS, A. P. and CROW, K. D. 2018. The evolution of underwater flight: The redistribution of pectoral fin rays in manta rays and their relatives (Myliobatidae). *Journal of Morphology*, **279**, 1155–1170.

HOVESTADT, D. C. and HOVESTADT-EULER, M. 1999. *Weissobatis micklichi* n. gen., n. sp., an eagle ray (Myliobatiformes, Myliobatidae) from the Oligocene of Frauenweiler (Baden-Württemberg, Germany). *Paläontologische Zeitschrift*, **73**, 337–349.

HOVESTADT, D. C. and HOVESTADT-EULER, M. 2013. Generic assessment and reallocation of Cenozoic myliobatins based on new information of tooth, tooth plate and caudal spine morphology of extant taxa. *Palaeontos*, **24**, 1–66.

JABADO, R. W., EBERT, D. A. and AL DHAHERI, S. S. 2022. Resolution of the *Aetomylaeus nichofii*

species complex, with the description of a new eagle ray species from the northwest Indian Ocean and a key to the genus *Aetomylaeus* (Myliobatiformes: Myliobatidae). *Marine Biodiversity*, **52**, 1–15.

KOLMANN, M. A., MARQUES, F. P. L., WEAVER, J. C., DEAN, M. N., FONTENELLE, J. P. and LOVEJOY, N. R. 2022. Ecological and phenotypic diversification after a continental invasion in Neotropical freshwater stingrays. *Integrative and Comparative Biology*, **25**, 424–440.

LAST, P., WHITE, W., DE CARVALHO, M., SÉRET, B., STEHMANN, M. and NAYLOR, G. J. P. 2016. *Rays of the world*. CSIRO Publishing, Clayton North.

LIM, K. C., LIM, P. E., CHONG, V. C. and LOH, K. H. 2015. Molecular and morphological analyses reveal phylogenetic relationships of stingrays focusing on the family dasyatidae (myliobatiformes). *PLoS ONE*, **10**, 1–21.

MARRAMÀ, G., KLUG, S., DE VOS, J. and KRIWET, J. 2018. Anatomy, relationships and palaeobiogeographic implications of the first Neogene holomorphic stingray (Myliobatiformes: Dasyatidae) from the early Miocene of Sulawesi, Indonesia, SE Asia. *Zoological Journal of the Linnean Society*, **184**, 1142–1168 .

MARRAMÀ, G., CARNEVALE, G., NAYLOR, G. J. P. and KRIWET, J. 2019*a*. Reappraisal of the Eocene whiptail stingrays (Myliobatiformes, Dasyatidae) of the Bolca Lagerstätte, Italy. *Zoologica Scripta*, **48**, 168–184.

MARRAMÀ, G., CARNEVALE, G., NAYLOR, G. J. P. and KRIWET, J. 2019*b*. Mosaic of plesiomorphic and derived characters in an Eocene myliobatiform batomorph (Chondrichthyes, Elasmobranchii) from Italy defines a new, basal body plan in pelagic stingrays. *Zoological Letters*, **5**, 1–18.

MARRAMÀ, G., CARNEVALE, G., NAYLOR, G. J. P. and KRIWET, J. 2020. Skeletal anatomy, phylogenetic relationships, and paleoecology of the Eocene urolophid stingray *Arechia crassicaudata* (Blainville, 1818) from Monte Postale (Bolca Lagerstätte, Italy). *Journal of Vertebrate Paleontology*, **40**, e1803339.

MARRAMÀ, G., CARNEVALE, G., GIUSBERTI, L., NAYLOR, G. J. P. and KRIWET, J. 2019*c*. A bizarre Eocene dasyatoid batomorph (Elasmobranchii, Myliobatiformes) from the Bolca Lagerstätte (Italy) reveals a new, extinct body plan for stingrays. *Scientific Reports*, **9**, 1–17.

MARTIN, L. K. and CAILLIET, G. M. 1988. Aspects of the reproduction of the bat ray, *Myliobatis californica*, in Central California. *Copeia*, **1988**, 754.

MARTINEZ, C. M., ROHLF, F. J. and FRISK, M. G. 2016. Re-evaluation of batoid pectoral morphology reveals novel patterns of diversity among major lineages. *Journal of Morphology*, **277**, 482–493.

MULVANY, S. and MOTTA, P. J. 2013. The morphology of the cephalic lobes and anterior pectoral fins in six species of batoids. *Journal of Morphology*, **274**, 1070–1083.

NAYLOR, G. J. P., CAIRA, J. N., JENSEN, K., ROSANA, K. A. M., STRAU, N. and LAKNER, C. 2012. Elasmobranch Phylogeny: A Mitochondrial Estimate Based on 595 Species. *Biology of Sharks and Their Relatives*, 47–72 pp.

NISHIDA, K. 1990. Phylogeny of the suborder Myliobatidoidei. *Memoirs of the Faculty of Fisheries Hokkaido University*, **37**, 1–108.

O'SHEA, O. R., BRACCINI, M., MCAULEY, R., SPEED, C. W. and MEEKAN, M. G. 2013. Growth of tropical dasyatid rays estimated using a multi-analytical approach. *PLoS ONE*, **8**, e77194.

SCHAEFER, J. T. and SUMMERS, A. P. 2005. Batoid wing skeletal structure: Novel morphologies, mechanical implications, and phylogenetic patterns. *Journal of Morphology*, **264**, 298–313.

SERENO, P. C. 2008. Logical basis for morphological characters in phylogenetics. *Cladistics*, **23**, 565–587.

SMITH, J. W. and MERRINER, J. V. 1987. Age and growth, movements and distribution of the cownose ray, *Rhinoptera bonasus*, in Chesapeake Bay. *Estuaries*, **10**, 153–164.

STEPANEK, R. and KRIWET, J. 2015. Comparative morphology of the juvenile skeleton in freshwater stingrays with special focus on *Paratrygon aiereba* (Myliobatiformes: Potamotrygonidae). *Zoologischer Anzeiger*, **255**, 7–24.

SWENSON, J. D., KLOMP, J., FISHER, R. A. and CROW, K. D. 2018. How the devil ray got its horns: The evolution and development of cephalic lobes in myliobatid stingrays (Batoidea: Myliobatidae). *Frontiers in Ecology and Evolution*, **6**, 181.

UNDERWOOD, C. J., KOLMANN, M. A. and WARD, D. J. 2017. Paleogene origin of planktivory in the Batoidea. *Journal of Vertebrate Paleontology*, **37**, e1293068.

VILLAFAÑA, J. A., MARRAMÀ, G., HERNANDEZ, S., CARRILLO-BRICENO, J. D., HOVESTADT, D., KINDLIMANN, R. and KRIWET, J. 2019. The Neogene fossil record of *Aetomylaeus* (Elasmobranchii, Myliobatidae) from the south-eastern Pacific. *Journal of Vertebrate Paleontology*, **39**, e1577251.

VILLALOBOS-SEGURA, E., MARRAMÀ, G., CARNEVALE, G., CLAESON, K. M., UNDERWOOD, C.

J., NAYLOR, G. J. P. and KRIWET, J. 2022. The phylogeny of rays and skates (Chondrichthyes: Elasmobranchii) based on morphological characters revisited. *Diversity*, **14**, 456.

YELDAN, H., AVSAR, D. and MANAŞIRLI, M. 2009. Age, growth and feeding of the common stingray (*Dasyatis pastinaca*, L., 1758) in the Cilician coastal basin, northeastern Mediterranean Sea. *Journal of Applied Ichthyology*, **25**, 98–102.

YIGIN, C. C. and ISMEN, A. 2012. Age, growth and reproduction of the common stingray, *Dasyatis pastinaca* from the North Aegean Sea. *Marine Biology Research*, **8**, 644–653.
